# Comparative analysis of the phytocyanin gene family in 10 plant species: a focus on *Zea mays*

**DOI:** 10.3389/fpls.2015.00515

**Published:** 2015-07-13

**Authors:** Jun Cao, Xiang Li, Yueqing Lv, Lina Ding

**Affiliations:** Institute of Life Sciences, Jiangsu UniversityZhenjiang, China

**Keywords:** phytocyanins, expansion, evolution, expression profile, maize

## Abstract

Phytocyanins (PCs) are plant-specific blue copper proteins, which play essential roles in electron transport. While the origin and expansion of this gene family is not well-investigated in plants. Here, we investigated their evolution by undertaking a genome-wide identification and comparison in 10 plants: *Arabidopsis*, rice, poplar, tomato, soybean, grape, maize, *Selaginella moellendorffii, Physcomitrella patens*, and *Chlamydomonas reinhardtii*. We found an expansion process of this gene family in evolution. Except PCs in *Arabidopsis* and rice, which have described in previous researches, a structural analysis of PCs in other eight plants indicated that 292 PCs contained N-terminal secretion signals and 217 PCs were expected to have glycosylphosphatidylinositol-anchor signals. Moreover, 281 PCs had putative arabinogalactan glycomodules and might be AGPs. Chromosomal distribution and duplication patterns indicated that tandem and segmental duplication played dominant roles for the expansion of *PC* genes. In addition, gene organization and motif compositions are highly conserved in each clade. Furthermore, expression profiles of maize *PC* genes revealed diversity in various stages of development. Moreover, all nine detected maize *PC* genes (*ZmUC10, ZmUC16, ZmUC19, ZmSC2, ZmUC21, ZmENODL10, ZmUC22, ZmENODL13*, and *ZmENODL15*) were down-regulated under salt treatment, and five *PCs* (*ZmUC19, ZmSC2, ZmENODL10, ZmUC22*, and *ZmENODL13*) were down-regulated under drought treatment. *ZmUC16* was strongly expressed after drought treatment. This study will provide a basis for future understanding the characterization of this family.

## Introduction

Blue copper proteins are ancient, type-I copper-containing proteins, which function as electron transporters in bacteria and plants (Giri et al., 2004). Blue copper proteins in plant are defined as phytocyanins (PCs), which include plastocyanins and some phytocyanin-related proteins (De Rienzo et al., 2000). Structurally, PCs consist of two conserved disulfide bridged Cys residues, four copper ligands, and an eight-stranded β-sandwich fold (Hart et al., 1996). According to the glycosylation state, copper ligand residues, domain organization, and spectroscopic properties of proteins, PCs can be divided into four groups: plantacyanins (PLCs), uclacyanins (UCs), stellacyanins (SCs), and early nodulin-like proteins (ENODLs; Nersissian et al., 1998; Ma et al., 2011; Li et al., 2013). PLCs contain a copper binding site consisting of one Met, one Cys, and two His ligands (Guss et al., 1996). And the N-terminal leader sequences in PLCs usually contain the endoplasmic reticulum target signal peptides (Nersissian et al., 1998). Although UCs also include the same four residues as described above in their copper-binding sites, they contain another domain resembling a cell-wall structural proteins (glycoproteins; Nersissian et al., 1998). SCs use a Gln residue as a copper ligand, while PLCs and UCs have a Met residue in this position (Nersissian et al., 1998). Like UCs, SCs consist of a copper-binding domain and a glycoprotein-like domain. The structure of ENODLs is similar to that of UCs and SCs, but ENODLs cannot bind copper, which might be involved in process without copper-binding (Greene et al., 1998; Mashiguchi et al., 2009; Ma et al., 2011).

Previous studies have indicated that PCs are involved in various plant activities, including cell differentiation and reorganization (Fedorova et al., 2002; Kato et al., 2002), pollen tube germinating and anther pollination (Kim et al., 2003; Dong et al., 2005), reproductive potential determining (Khan et al., 2007), apical buds organ development (Mashiguchi et al., 2009), and somatic embryogenesis (Poon et al., 2012), etc. In addition, PCs may also function in stress responses, including enhancing osmotic tolerance (Wu et al., 2011), inhibiting aluminum absorption and protecting cell from aluminum toxicity (Ezaki et al., 2001, 2005). Several researches have indicated that salt and drought stresses can induce the expression of some *PC* genes, suggesting the potential response to abiotic stresses (Ozturk et al., 2002; Ma et al., 2011).

To date, through a comprehensive bioinformatics analysis, only 38, 62, and 84 *PC* genes have been identified in *Arabidopsis*, rice and *Brassica rapa*, respectively (Mashiguchi et al., 2009; Showalter et al., 2010; Ma et al., 2011; Li et al., 2013). In the present study, including *Arabidopsis* and rice, we identified the *PC* gene family of 10 species in plants, and each species contains 1–89 *PC* genes. Considering the important roles associated with developmental functions and stress responses, and the number of the *PC* genes varied largely among plant species, it’s of considerable interest to us to research how the *PC* genes have evolved in Plantae, and how and why different plant species have obtained such different *PC* genes. Here, our results indicate that the *PC* gene family has an expansion process in plant evolution, and that tandem and segmental duplications and retrotransposition play dominant roles for their expansion. Our studies also reveal diverse expression patterns of the *PC* genes in maize.

## Materials and Methods

### Identification of the *PC* Genes Plants and Bioinformatics Analysis

We first used *Arabidopsis*, rice and *B. rapa PC* sequences (Mashiguchi et al., 2009; Showalter et al., 2010; Ma et al., 2011; Li et al., 2013) as queries in basic local alignment search tool (BLAST) searches against the phytozome^1^ (Goodstein et al., 2012) with -1 expect (E) threshold to identify potential members of the *PC* gene family in plants. The sequences were then confirmed as encoding PC for the presence of a plastocyanin-like domain (PCLD) signature by the Pfam (Punta et al., 2012) searches. Subsequently, SignalP 4.1 Server (Petersen et al., 2011) was used to check the signal peptide (SP) of all proteins. Big-PI Plant Predictor (Eisenhaber et al., 2003) was used to predict the glycosylphosphatidylinositol (GPI)-anchor signal. In addition, we also used NetNGlyc 1.0 Server^2^ to predict the N-glycosylation sites in PC proteins. Putative arabinogalactan (AG) glycomodules were predicted mainly following the previously described criteria (Schultz et al., 2002; Showalter et al., 2010; Ma et al., 2011). The structure characteristics of PCs are shown in Supplementary Table S1.

### Phylogenetic Analyses of the *PC* Gene Family in Plants

We used MUSCLE 3.52 (Edgar, 2004) to perform multiple sequence alignments of full-length protein sequences. And neighbor-joining (NJ) method in MEGA v5 (Tamura et al., 2011) was used to carry out phylogenetic analyses of the PC proteins with Dayhoff methods and default assumptions. Bootstrap analyses with 1,000 replicates were used to test support.

### Estimation of the Maximum Number of Gained and Lost *PCS*

Next, we divided the phylogeny into different clades to determine the expansion extent of *PC* gene family in different plant lineages. Nodes among lineages denoted the most recent common ancestor (MRCA) and were labeled as V: Viridiplantae; E: Embryophyte; T: Tracheophyte; A: Angiosperm; G: Grass; Eu: eudicots; R: Rosid. Notung v2.6 (Chen et al., 2000) was used to infer gene loss and duplication events.

### Conserved Motifs Analyses

MEME program^3^ (Bailey et al., 2006) was used to identify motifs in the plant PC proteins. This program was run with the following parameters: maximum number of motifs = 8, number of repetitions = any, and with optimum motif widths between 6 and 50 residues.

### Chromosomal Location and Exon–Intron Structure Analysis

We used the annotation information of the *PC* genes on phytozome^1^ (Goodstein et al., 2012) to determine their chromosomal locations. The segmental duplication (or syntenic) regions of the different chromosomes in maize and *Arabidopsis* genomes were calculated with the Synteny Mapping and Analysis Program (SyMap; Soderlund et al., 2011). Genomicus^4^ online tool (Louis et al., 2013) was used to explore the *PC* gene organization information within and between genomes. The exon–intron structure of *PC* genes was also collected from genome annotations.

### Estimating the Age of Duplicated Paralog Gene Pairs

We first determined paralogous gene pairs by the protein phylogeny, and used them as references for a multiple alignment of DNA coding sequences using embedded ClustalW (codons) software in MEGA v5 (Tamura et al., 2011). And we used K-Estimator 6.0 program (Comeron, 1999) to estimate the *K_a_* and *K_s_* values of paralogous genes. The approximate data of the duplication event for each of gene pair was calculated using the formula (T = *K_s_*/2λ), assuming the clock-like rate (λ) is 1.5 × 10^-8^ and 6.5 × 10^-9^ synonymous/substitution site/year for *Arabidopsis* (Koch et al., 2000) and for maize (Gaut et al., 1996), respectively.

### Microarray-Based Expression Analysis

We used the Plant Expression Database (PLEXdb; Dash et al., 2012) for expression analyses of maize *PC* genes. One experiment (ZM37) contributed by Kaeppler group in Sekhon et al. (2011) was selected in this study. Expression data in 34 selected tissues were gene-wise normalized in the Genesis (v 1.7.6) program (Sturn et al., 2002).

### Plant Materials and Treatment

We used 1-week-old maize (*Zea mays* L. inbred line B73) seedlings to examine the expression patterns of *PC* genes under salt and drought stresses. Plants were grown in a plant growth chamber at 23 ± 1°C with a 14 h light/10 h dark photoperiod. Control (CK) seedlings were grown with normal irrigation. For salt treatment, the maize seedlings were kept in 150 mM NaCl for 24 h. For drought treatment, the seedlings were dried between folds of tissue paper at 23 ± 1°C for 3 h. Each sample was conducted three replicates.

### RNA Isolation and Quantitative Real-Time PCR (QRT-PCR) Analysis

Trizol total RNA extraction kit (Sangon, Shanghai, China) was used to extract total RNA. Next, moloney murine leukemia virus (M-MLV) reverse transcriptase (TakaRa, Dalian, China) was used to perform reverse transcription. Triplicate quantitative assays were performed using SYBR Green Master Mix (TakaRa) with an ABI 7500 sequence detection system. Nine maize *PC* genes were randomly selected for real-time quantitative reverse transcription polymerase chain reaction (qRT-PCR) analysis. The gene-specific primers (**Table 3**) were synthesized in Sangon. The expression level of *Actin 1* (*GRMZM2G126010*) gene was used as a reference. 2^-ΔΔCT^ method (Livak and Schmittgen, 2001) was used to calculate the relative expression level of the *PC* genes.

## Results and Discussion

### Identification of *PC* Multigene Family in Plants

Phytocyanins are plant-specific ancient blue copper proteins which function as electron transporter. Though some researches (Mashiguchi et al., 2009; Showalter et al., 2010; Ma et al., 2011; Li et al., 2013) have been made in the characterization of plant PCs during the past decade, studies on this gene family are still scarce. In order to identify *PC* multigene families in other plant species, we used *Arabidopsis*, rice and *B. rapa* PC proteins as queries to perform a genome-wide search in eight genomes in Viridiplantae. The returned sequences were further confirmed as encoding PC by the Pfam (Punta et al., 2012) searches for the presence of a plastocyanin-like domain (PCLD) signature conserved in other PC proteins. As we know, the *Arabidopsis* and rice PCs have been bioinformatically and systematically studied in previous study (Ma et al., 2011; Li et al., 2013), so, the previous published data were also used to carry out deeper analysis. As a result, a total of 465 *PC* genes were identified from 10 plants in the phytozome database (**Table 1**). Our analysis shows that the number of *PC* genes ranged from 1 to 89 across the different plant species (**Table 1**). The soybean genome contains a maximum of 89 *PC* genes, while, chlamydomonas has only one. About 60 and 77 putative *PC* genes were identified from maize and poplar, respectively. Poplar has about two times *PC* genes than *Arabidopsis*, whereas rice and maize have a similar number of the *PC* genes when compared with that of poplar. By searching the Genome database of NCBI^5^, we found that the poplar, *Arabidopsis* and maize genomes contain 42,577, 33,583, and 39,454 genes, respectively, which are 39.4, 9.9, and 29.2% larger than that of rice (30,534), respectively. This implied that the number of *PCs* is not proportional to the size of the genomes. Obviously, there will be some forces to prompt the number change of this gene family in different plant species.

**Table 1:** *PC* genes identified in 10 sequenced plants.

Lineage	Organism	Genome size (Mb)^∗^	No. of predicted genes^∗^	No. of PC genes
Algae	*Chlamydomonas reinhardtii*	120.41	14488	1
Moss	*Physcomitrella patens*	477.95	35936	28
Lycophytes	*Selaginella moellendorffii*	212.5	34782	20
Dicots	*Arabidopsis thaliana*	119.67	33583	38
	*Populus trichocarpa*	485.67	42577	77
	*Vitis vinifera*	486.26	28268	41
	*Solanum lycopersicum*	781.51	27466	49
	*Glycine max*	973.49	50202	89
Monocots	*Oryza sativa*	382.78	30534	62
	*Zea mays*	2065.7	39454	60
Total				465

*^∗^The data come from www.ncbi.nih.gov/genome/.*

### Structural Analysis of the Putative PC Proteins

**FIGURE 1:**
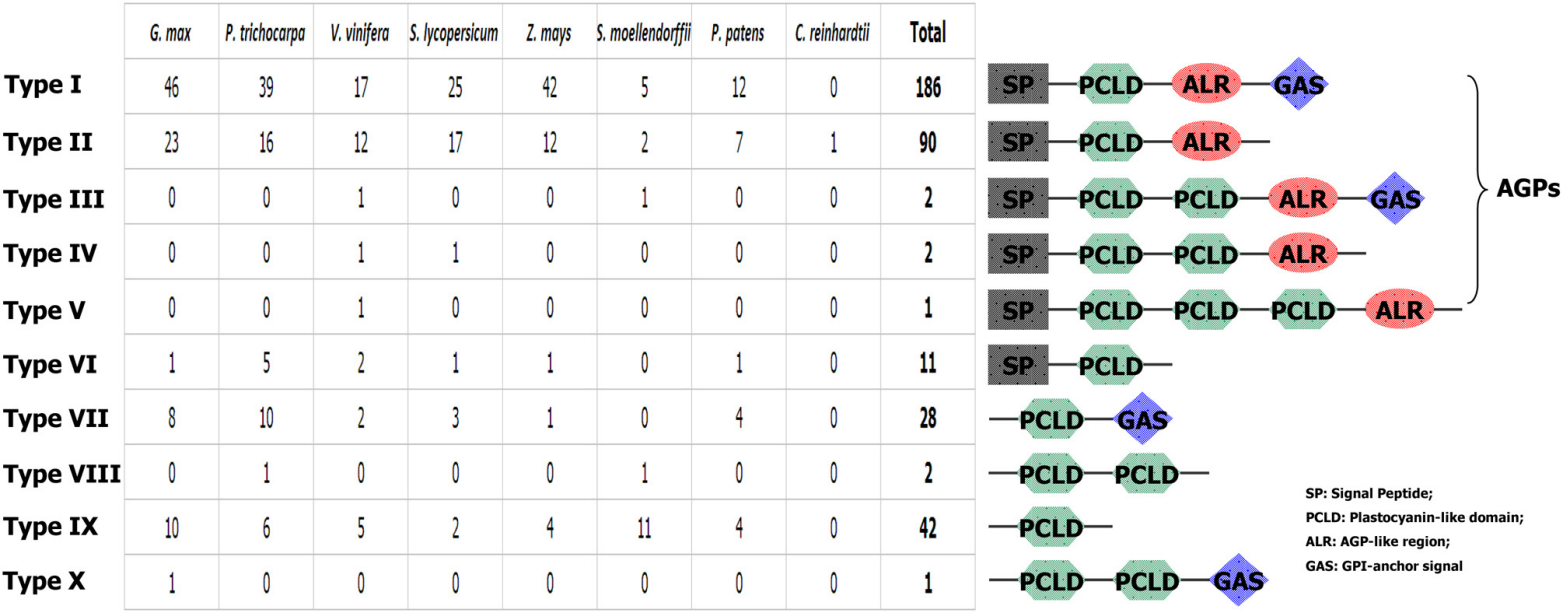
**Graphical representation of 10 types of PCs and their comparative analysis among the eight plant species**.

To further investigate the structural characteristics of PC proteins, we used several bioinformatics websites as described in the materials and methods section to predict the AG glycomodules, SPs, GPI-anchor signals (GASs), and N-glycosylation sites of PCs. Our results (Supplementary Table S1) indicated that 292 PCs were predicted to contain an N-terminal SP required for targeting to the endoplasmic reticulum. In addition, 217 PCs were expected to have GASs responsible for plasma membrane localization. The subcellular localizations of plant PCs have been found to correlate with their specific functions. For example, AtSC3/AtBCB, an *Arabidopsis* blue copper binding protein, was strongly localized in the plasma membrane and induced by aluminum stress and oxidative stress, suggesting that the plant PCs may participate in some abiotic stress responses (Ezaki et al., 2001, 2005). Additionally, PC proteins accumulated in the sieve element plasma membrane may be involved in determining reproductive potential (Khan et al., 2007). Moreover, 281 PCs had putative AG glycomodules in the (Pro, Ala, Ser, Thr)-rich region. These 281 PCs might be AGPs for the existence of AG glycomodules and SPs. According to the distribution of the SP, PCLD, AGP-like region (ALR) and GAS, these PCs were separated into ten types (**Figure 1**). Type I PCs had typical properties, including an N-terminal SP, a PCLD, an ALR, and a C-terminal GAS. Type II PCs were short of GAS, while other features were similar to type I. Both GAS and ALR were absent from type VI, VIII, and IX PCs. Interestingly, we also found that type III, IV, VIII, and X PCs possessed two PCLDs, and type V had three PCLDs. The domain repeats are usually thought to evolve through recombination events and intragenic duplication (Björklund et al., 2006). The creation of new multi-domain architectures is an important mechanism that provides opportunities for the organism to expand its repertoire of cellular functions, such as transcriptional regulation, protein transport and assembly (Andrade et al., 2001; D’Andrea and Regan, 2003; Weiner et al., 2006). Furthermore, protein domain repeats may constitute a source of variability. In human genome, duplications are more common in genes containing repeated domains than in non-repeated ones (Björklund et al., 2010). The domain repetition is quite important in evolution, since it provides a path where proteins can evolve through removing or adding functionally similar or distinct blocks (Light et al., 2012). In this study, we identified some multi-PCLD domains in PCs. This presence of PCLD domain repeats contribute to the complexity of this gene family. Its effect on the function of PC proteins remains to be examined. However, our findings suggest that the PCLD repeats may play an important role in PC protein evolution.

### Origin and Contrasting Changes in the Numbers of Plant *PC* Genes

**FIGURE 2:**
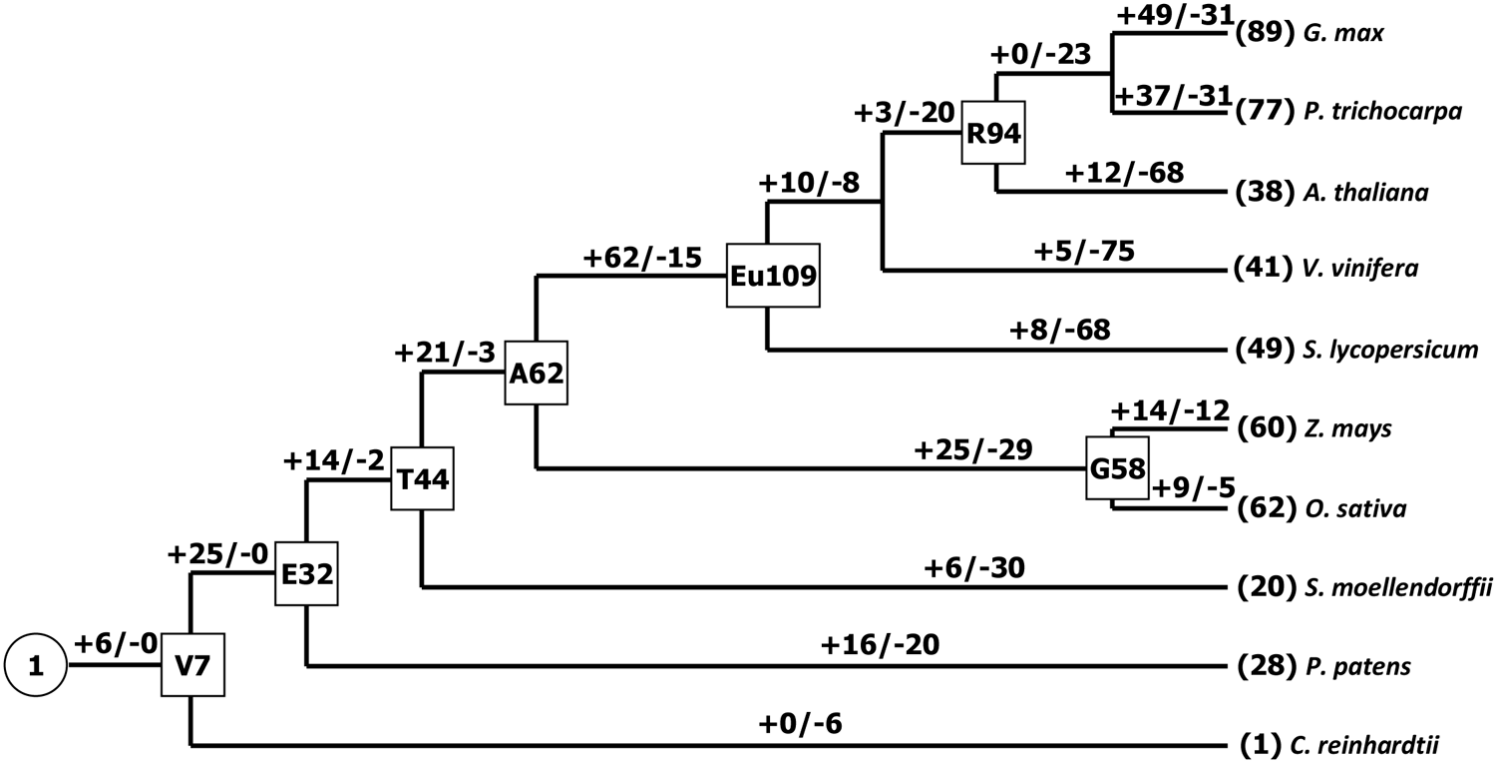
**Gain and loss of the *PC* genes in plant evolution.** Seven internal nodes (V, Viridiplantae; E, Embryophyte; T, Tracheophyte; A, Angiosperm; G, Grass; Eu, eudicots; R, Rosid) are shown in the rectangles. Plus and minus signs indicate gene gain and loss events, respectively.

It has been suggested that the Chlorophycean is the primitive species in Viridiplantae from which all land plants have evolved (Misumi et al., 2008). The earliest *PCs* possibly originated about 1 billion years ago in algae (Merchant et al., 2007; Misumi et al., 2008). Our search for *PCs* in *Chlamydomonas reinhardtii* found only one member. Therefore, the origin of the plant *PC* genes could be traced to the ancient algae. The *PC* gene family appeared to expand by duplication events. For example, *Physcomitrella patens* has 28 *PC* genes, which soybean exhibites 89 paralogous gene sequences representing about 19% of total 465 identified *PCs*, which might be due to at least three whole genome duplications (Schmutz et al., 2010). As we know, expansion and conservation of a gene family in evolution imply important roles during organism adaptation to environment (Cao et al., 2011; Cao and Shi, 2012). Next, we also estimated the number of *PC* genes in the MRCA to better understand how this family gene has evolved in Viridiplantae. Reconciliation of the species phylogeny with the gene trees suggested that one ancestral *PC* gene exist in the MRCA of Viridiplantae. Furthermore, we identified 32 orthologous genes in the Embryophyte MRCA and 44 in the MRCA of Tracheophyte (**Figure 2**). We also found that the number of *PCs* remained relatively increased from the land plants (*P. patens*) to the angiosperms. Eudicot ancestral *PCs* once more expanded significantly after the separation from monocot species about 145 million years ago (Xu et al., 2009). We identified about 109 ancestral *PC* genes in the MRCA of eudicots. After that, many *PC* genes have lost in the eudicots. It appeared that the *PC* family had been reduced in all the analyzed eudicot species compared with the number of MRCA in eudicots. For example, the number of *PCs* decreased approximately 65.1 and 55 percent for *Arabidopsis* and tomato, respectively. Whereas when compared the number of ancestral *PC* genes, it appeared that this family had expanded in all the extant species. In addition, this expansion was uneven among these plant species. For instance, there are 77, 60, 41, and 28 genes in poplar, maize, grape, moss, respectively, while the estimated numbers of genes in the MACA of Viridiplantae are seven. Therefore, poplar, maize, grape and moss have gained 70, 53, 34 and 21 genes, respectively, since their splits. The numbers of genes gained in the soybean lineage are much greater than that in other lineages.

### Chromosomal Distribution and Duplication Patterns of *PC* Genes in Plants

**FIGURE 3:**
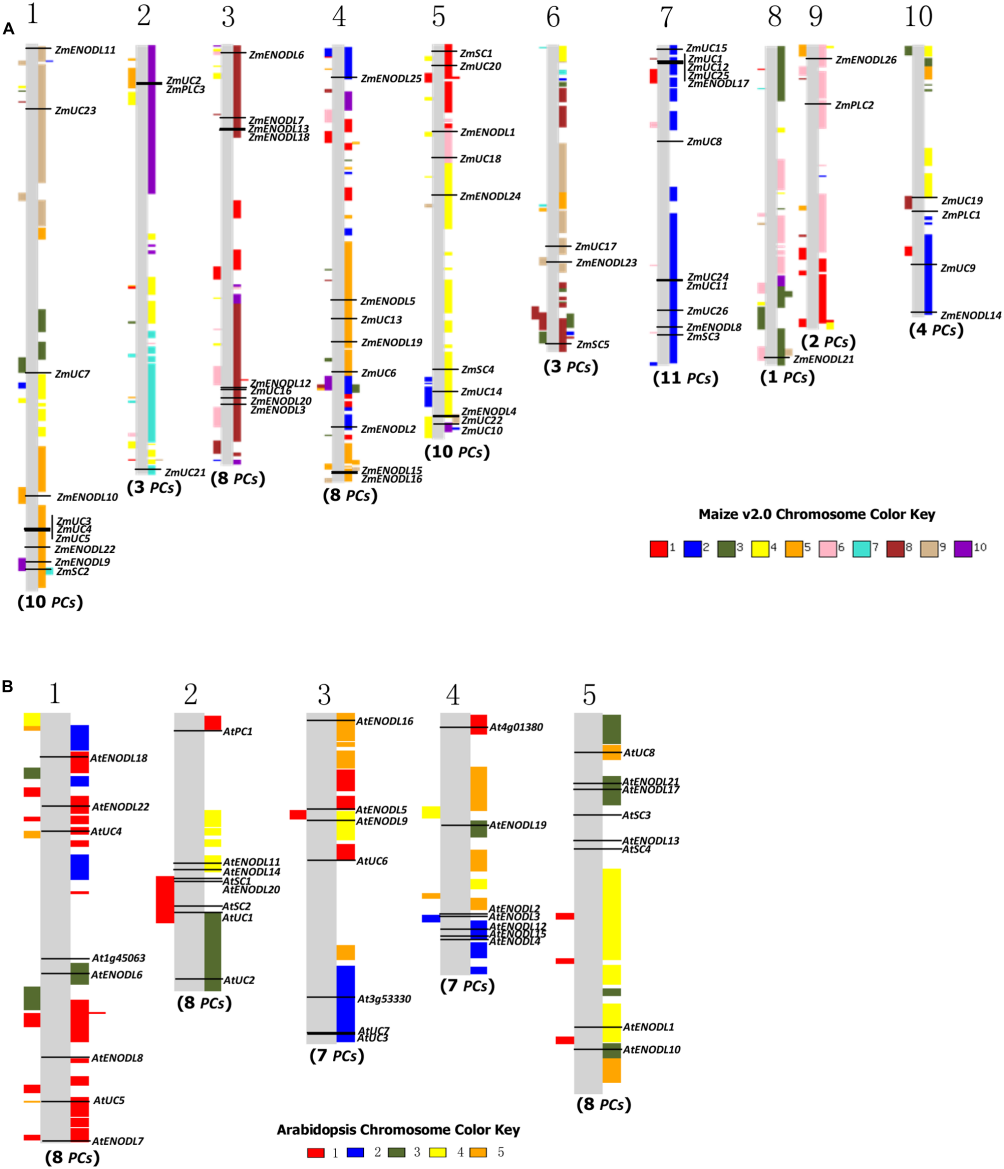
**Gene locations and genomic duplication in maize **(A)** and *Arabidopsis* (B).** SyMAP v3.4 (Soderlund et al., 2011) was used to depict the paralogous regions in the putative ancestral constituents of the maize **(A)** and *Arabidopsis* **(B)** genomes. Moreover, some relationship of orthologs or paralogs was confirmed with the Genomicus (http://www.dyogen.ens.fr/genomicus/) online tool (Louis et al., 2013). The segmental duplication regions are supposed to be colored with the same way to the relevant chromosome color. For example, the color key of maize chromosome 1 is red, which means that all red regions on the chromosomes are segmental duplicated from the chromosome 1. Using this method, we can get all of the paralogous regions among different chromosomes in the genome.

Gene duplication, which usually occurs via segmental duplication, tandem duplication and retrotransposition, plays important roles in organismal evolution (Chen et al., 2014; Cao and Li, 2015). To search for duplication mechanisms for *PC* genes, as examples, we examined their genomic distribution in *Arabidopsis* and maize. The results showed that *PC* genes are dispersed throughout *Arabidopsis* and maize genomes (**Figure 3**). We also found that about 79.5 and 96.7% of *PC* genes locate on the duplicated segments of chromosomes in *Arabidopsis* and maize, respectively. Within identified duplication events, 5 of 11 pairs (*AtENDOL14/AtENODL15, AtENODL5/AtENODL6, AtUC4/AtUC5, AtENODL1/AtENODL2*, and *AtENODL11/AtENODL12*) in *Arabidopsis* and 7 of 20 pairs (*ZmUC6/ZmUC10, ZmUC13/ZmUC14, ZmSC1/ZmSC2, ZmUC22/ZmPLC2, ZmENODL4/ZmENODL19, ZmENODL12/ZmENODL21, ZmENODL16/ZmENODL24*) in maize are retained (**Figure 3**). In addition, evolutionary dates of these duplicated *PC* genes were also estimated (**Table 2**). The result indicated that duplication events for *Arabidopsis* six pairs and maize seven pairs occurred within the past 19.73–28.58 million years and 11.72–21.16 million years, respectively (**Table 2**). These periods coincide with the time of the secondary large-scale genome duplication in *Arabidopsis* and maize (Gaut et al., 1996; Koch et al., 2000). In addition, we also observed some earlier segmental duplication events occurred around from 41.63 to 58.33 MYA in the *PCs* of *Arabidopsis* (*AtENODL1/AtENODL2* and *AtENODL11/AtENODL12*) and maize (*ZmUC3/ZmUC23* and *ZmUC22/ZmPLC2*), nearly within or following grasses origination (Kellogg, 2001). Interestingly, we also found that about 31.67% of *PC* genes were tandemly clustered in maize, and only one clustered *PCs* (*AtUC7*-*AtUC3*) were also identified in *Arabidopsis* (**Figure 3**), suggesting that tandem duplication may be another factor generating the family genes. In a word, segmental duplication and tandem duplication contribute to the expansion of the *PC* gene family.

**Table 2:** Inference of duplication time of *PC* paralogous pairs in *Arabidopsis* and maize.

Paralogous pairs	*K_a_*	*K_s_*	*K_a/_K_s_*	Duplication types	Data (million years ago)
*AtENODL17/AtENODL19*	0.18395	0.70102	0.26243	Retrotransposition	23.37
*AtENODL3/AtENODL4*	0.13741	0.31929	0.43036	Retrotransposition	10.64
*AtENODL14/AtENODL15*	0.18437	0.70152	0.26282	Segmental duplication	23.38
*AtENODL5/AtENODL6*	0.22558	0.77783	0.29001	Segmental duplication	25.93
*AtUC4/AtUC5*	0.31272	0.85669	0.36503	Segmental duplication	28.56
*AtENODL1/AtENODL2*	0.46645	1.45391	0.32083	Segmental duplication	48.46
*AtENODL11/AtENODL12*	0.38216	1.36091	0.28081	Segmental duplication	45.36
*AtSC1/AtSC2*	0.19307	0.34871	0.55367	Retrotransposition	11.63
*At1g45063/At3g53330*	0.37509	0.71432	0.5251	Retrotransposition	23.81
*AtENODL22/AtPC1*	0.72483	3.00941	0.24085	Retrotransposition	100.31
*AtUC3/AtUC7*	0.22650	0.59184	0.3827	Tandem duplication	19.73
*ZmUC6/ZmUC10*	0.05401	0.18988	0.28444	Segmental duplication	17.26
*ZmUC11/ZmUC24*	0.32393	0.50672	0.63927	Tandem duplication	46.07
*ZmUC13/ZmUC14*	0.10750	0.18573	0.57879	Segmental duplication	16.88
*ZmUC16/ZmPLC3*	0.67658	0.88356	0.76574	Retrotransposition	80.32
*ZmUC8/ZmUC19*	0.49874	0.74853	0.66629	Retrotransposition	68.05
*ZmSC4/ZmSC5*	0.02184	0.01847	1.18246	Retrotransposition	1.68
*ZmSC1/ZmSC2*	0.11703	0.23277	0.50277	Segmental duplication	21.16
*ZmUC1/ZmUC12*	0.09926	0.15513	0.63985	Tandem duplication	14.10
*ZmUC3/ZmUC23*	0.40586	0.54119	0.74994	Retrotransposition	41.63
*ZmUC18/ZmUC26*	0.80249	0.91956	0.87269	Retrotransposition	70.74
*ZmENODL20/ZmENODL22*	0.64824	0.94528	0.68577	Retrotransposition	85.93
*ZmUC22/ZmPLC2*	0.43753	0.64158	0.68196	Retrotransposition	58.33
*ZmPLC1/ZmUC9*	0.05469	0.14448	0.37853	Retrotransposition	13.13
*ZmUC4/ZmUC5*	0.06773	0.12898	0.52512	Tandem duplication	11.72
*ZmENODL2/ZmENODL25*	0.56183	0.92832	0.60521	Retrotransposition	84.39
*ZmENODL4/ZmENODL19*	0.17464	0.33073	0.52804	Segmental duplication	30.07
*ZmENODL7/ZmENODL13*	0.0335	0.07531	0.44483	Retrotransposition	6.85
*ZmENODL3/ZmENODL6*	0.58564	0.95152	0.61548	Segmental duplication	86.50
*ZmENODL12/ZmENODL21*	0.07988	0.15477	0.51612	Segmental duplication	14.07
*ZmENODL16/ZmENODL24*	0.11364	0.31901	0.35623	Segmental duplication	29.00

Similar expansion patterns were also found in *Oryza sativa* and *B. rapa PC* genes (Ma et al., 2011; Li et al., 2013). In the rice genome, 20 of 62 *OsPC* genes were segmental duplications; while, 63 of 84 *BrPC* genes were attributed to segmental duplications in the *B. rapa*. This indicated that this type of duplication event contributes to the expansion of the *PC* genes in these plants. Tandem duplication is an important factor dramatically expanding new copies in clusters by unequal recombination or replication slippage (Anderson and Roth, 1977; Blanc and Wolfe, 2004; Cannon et al., 2004; Thomas, 2005). Initially, tandem duplicated genes have similar sequences and functions; but, in the subsequent evolution, they tend to divergence in structure and expression patterns during too many changes in the *cis*- and *trans*-acting effects, DNA sequences, regulatory networks, and chromatin modifications (Charon et al., 2012). Several previous studies have investigated these divergences between duplicate genes (Makova and Li, 2003; Li et al., 2005; Ganko et al., 2007). During the process of evolution, some duplicated genes were maintained the similarity of functions, while others either gained new functions (neofunctionalization) or subdivided their functions (subfunctionalization), or lost them (pseudogenization; Pinyopich et al., 2003; Franzke et al., 2010; Wang and Paterson, 2011). Plants cannot freely escape the changing environment. Therefore, some genes associated with stress defense are required to expand to resist these environmental stimulations. Previous studies have indicated that tandem duplicated genes are often involved in responses to environmental stimuli or stress in plants (Leister, 2004; Maere et al., 2005; Fang et al., 2012). Our results also indicated that about 31.67 and 29.03% of *PC* genes were tandemly clustered in monocots maize and rice, respectively. And some stress responses were often associated with the PC proteins (Ezaki et al., 2001, 2005; Ozturk et al., 2002; Ma et al., 2011; Wu et al., 2011). Amplification of the *PC* genes by tandem duplication in monocots maize and rice is regarded as a mechanism for protecting plants from harmful stresses, which may be crucial for organismal adaptation to different environments. Only one clustered *PCs* (*AtUC7*-*AtUC3*) were identified in *Arabidopsis*, and no duplicated *PC* genes were identified from tandem duplications in another eudicot *B. rapa* (Li et al., 2013), implying different expansion types of this gene family between monocots maize and rice and eudicots *Arabidopsis* and *B. rapa*.

We also found that the *K_a_/K_s_* values of the sequences among *PC* pairs were significantly different (**Table 2**). Moreover, except for the *ZmSC4/ZmSC5* gene pairs, all other’s estimated *K_a_/K_s_* values were less than 1, implying that most of the duplicated *PC* sequences within these pairs are under purifying selection pressure in evolution. The *K_a_/K_s_* value of *ZmSC4/ZmSC5* pairs is 1.18246, indicating that positive selection might be occurred between this gene pairs after duplication about 1.68 Mya. Gene or protein evolution is an outcome of the interplay between mutation and selection. During evolution, some functional regions have reached the optimal state. Therefore, most of the mutations that altered the function will be abandoned by purifying selection. With changes in environment, subsequent selective pressure spurs such regions to change to improve the fitness of the organism in a new environment accordingly. From this point, detecting positive selection seems especially necessary, because it can indicate selective advantages in changing the gene or protein sequences. These selective advantages are essential for understanding of functional regions of the gene or protein and functional shift (Morgan et al., 2010). In this study, one duplicated gene pairs (*ZmSC4/ZmSC5*) were identified to undergo positive selection after separated by duplication, implying that functional divergence of duplicated genes might have accelerated by positive selection during long periods of evolution. Thus, this might facilitate an adaption to different environments for the organism.

### Motif Distribution and Intron Loss in Some Clades

We used Pfam (Punta et al., 2012) to identify the major domains of PC proteins in plants. Results showed that all PC proteins possessed PCLD signature that is essential for electron transport activity. To recognize some smaller individual motifs, we used the MEME^6^ (Bailey et al., 2006) to study the diversification of PC proteins in plants. As a result, we identified eight distinct motifs in these members (**Supplementary Figure S1**). Obviously, most members in each clade have similar motif compositions, suggesting functional conservation of the PC proteins in the same clade (**Supplementary Figure S1**). Therefore, motif compositions of the PC proteins in each clade may provide additional support for the phylogenetic analyses (Cao, 2012).

**FIGURE 4:**
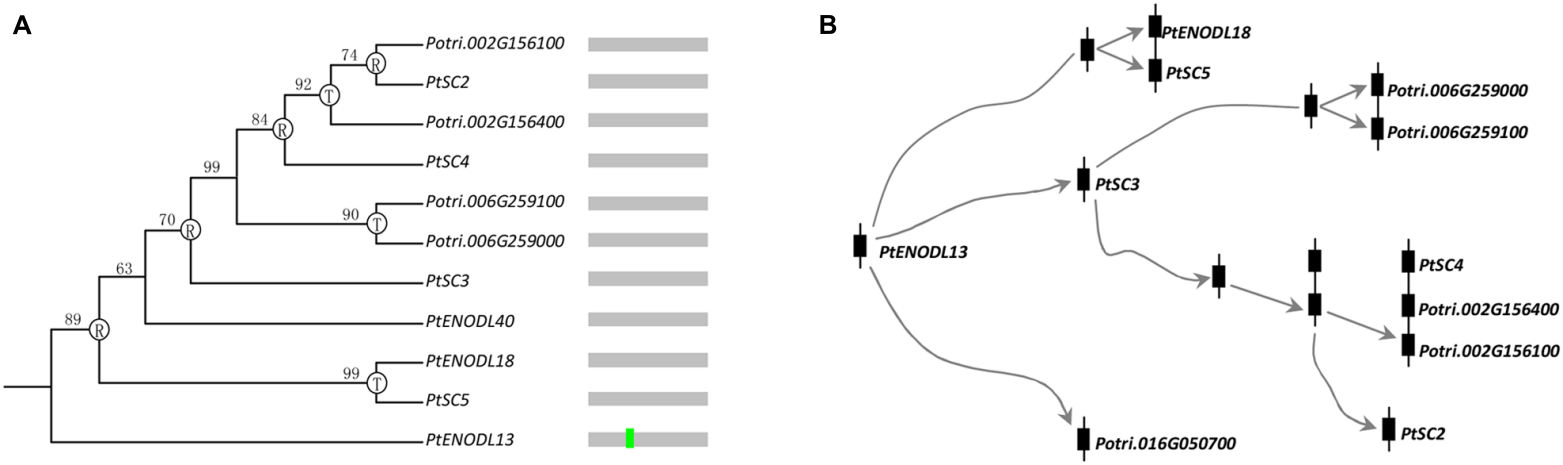
**Evolution of one *PC* clade in poplar. (A)** Phylogenetic relationships and intron insertion; **(B)** Hypothetical origins of eleven poplar *PC* genes by retroposition and tandem duplication. The letters “R” and “T” indicate the positions where retroposition and tandem duplication have occurred, respectively. Bright green vertical line represents conserved 1 phase intron insertion position in PCLD as shown in **Supplementary Figure S1**.

Exon–intron structure has been used to explain the evolutionary relationships (Cao et al., 2010; Koralewski and Krutovsky, 2011; Chen and Cao, 2014). Next, we compared the exon–intron organization of the *PCs* in 10 plants. **Supplementary Figure S1** provided a detailed illustration of the position of introns of each PCLD domain. Our results indicated a conserved 1 phase intron insertion in PCLD of most *PC* paralogs. Interestingly, we also found that this intron insertion has been lost in some poplar PCLD (**Supplementary Figure S1**). Moreover, these intronless genes in PCLD tended to form species-specific clusters on the poplar chromosomes 2, 6, and 15 (**Supplementary Figure S1**). It may be the consequences of retroposition and tandem duplications. The loss of intron in these *PCs* was likely associated with recent evolutionary expansion, like, retroposition and tandem duplication. To test this hypothesis, we identified the candidate donor gene based on the following two criteria. The first criterion is that the retrogene will have identical sequences to the donor gene after retroposition, so they will cluster together in a phylogenetic tree (Kong et al., 2007). Since retrogene comes from retroposition, it usually lacks specific introns compared with the donor gene. Therefore, the second criterion is that the donor gene can be judged from the presence/absence of the specific intron (Kong et al., 2007). **Figure 4** shows an example of intron loss caused by gene expansion. Genes with the conserved intron (such as, *PtENODL13*) usually locate basal positions of the phylogenetic tree, while genes without the intron (such as, others 10 *PCs* in the clade as shown in **Figure 4**) often form terminal clades. It is likely that *PtENODL13* contains the conserved intron and is their ancestor (donor gene), from which the intronless retrogenes were generated by retroposition and tandem duplication.

### Expression Profiles of the *PC* Genes in Maize

**FIGURE 5:**
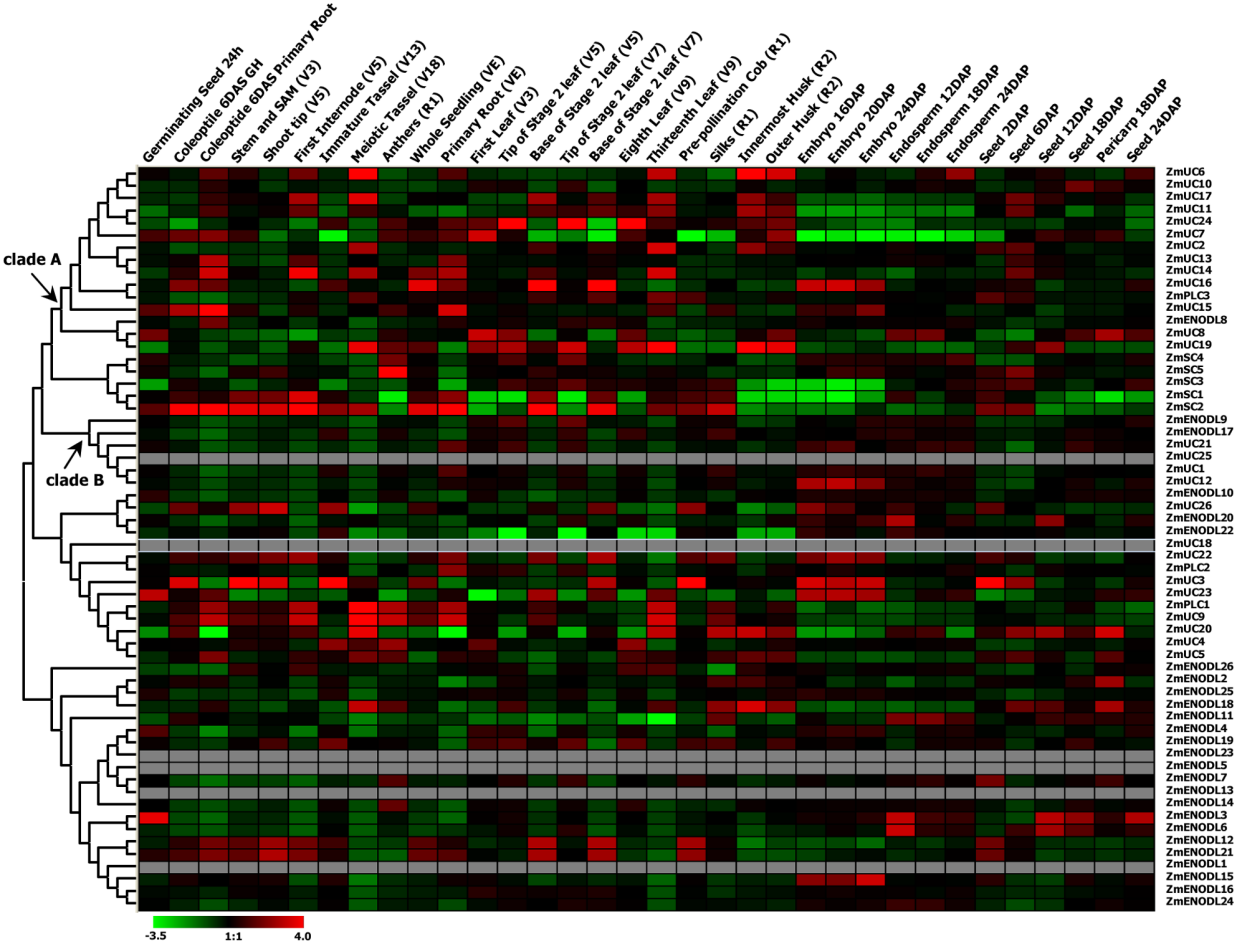
**Expression profiles of the maize *PC* genes.** Dynamic expression profiles of the maize *PC* genes in different development tissues.

**FIGURE 6:**
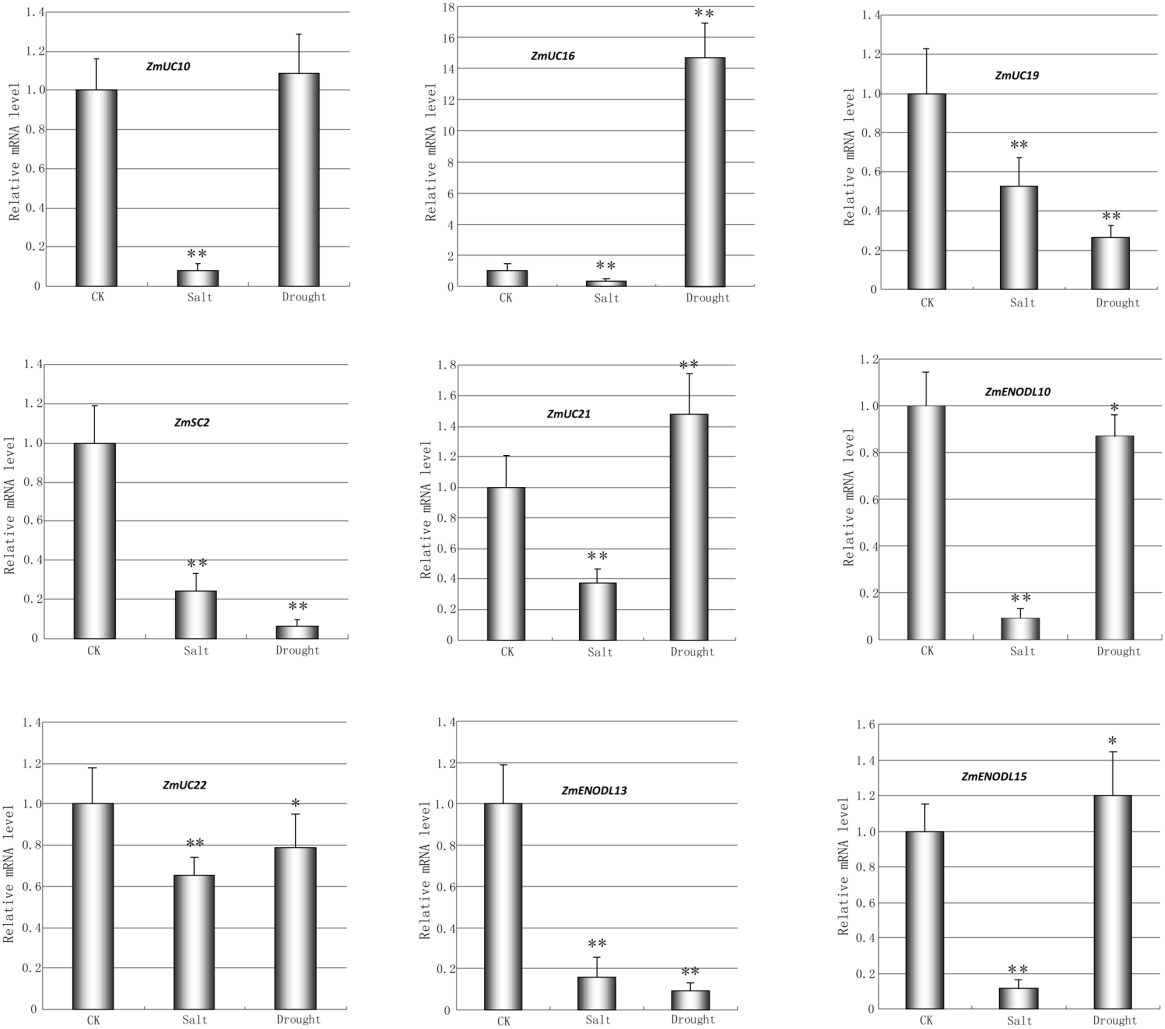
**Quantitative RT-PCR analysis of nine selected *ZmPC* genes under the salt and drought treatments.** The relative expression level of each transcript was shown here. Error bars indicate standard deviation (SD) of independent biological replicates. *Asterisk* indicates a significant difference from the control (^∗^*p* < 0.05; ^∗∗^*p* < 0.01).

We first used publicly available microarray data to detect the spatiotemporal expression patterns of the maize *PC* genes. Expression profiles of the *PC* genes were mined at 34 different tissues. Only 54 probes were detected standing for the 54 *ZmPC* transcripts. The remaining six transcripts with no detectable expression signal are *GRMZM2G463441, GRMZM2G136879, GRMZM2G148624, GRMZM2G047208, GRMZM2G085504*, and *AC209987.4_FGT010*. The results indicated that these genes are expressed variously in different tissues, implying that they may be involved in many growth and developmental processes (**Figure 5**). Such as, most Zm*PC* genes of clade A showed high expression levels in the root, leaf and internodes, but low expression levels in the endosperm and embryo. In contrast, *ZmPC* genes in clade B presented the oppositive results compared with clade A. That is, most members of clade B displayed high expression levels in the embryo and endosperm, but showed low level expression in the leaf, root and internodes. This suggested that *ZmPC* genes in different clades may be involved in various biological processes. Some *ZmPCs* were also found to be highly expressed in some specific organs, such as, *ZmSC5* in anthers, *ZmUC3* and *ZmUC23* in embryo, suggesting that they might be involved in the growth and development of these organs in maize. Similar results have also been observed in their homologs in *Arabidopsis* (*AtENODL1/5/6/7/11/12/16, AtAGP6/11*, and *FLA3*; Yu et al., 2005; Levitin et al., 2008; Li et al., 2010; Ma et al., 2011), rice (*OsENODL9/14/16/17*; Ma et al., 2011), and *B. rapa* (*BrENODL22/27* and *BrSCL8/9*; Li et al., 2013), which were highly expressed in reproductive organs. The functions of some *PC* genes have been investigated in several studies. For example, a sieve element-specific expressed gene (*AtENODL9*) may be involved in determining reproductive potential in *Arabidopsis* (Khan et al., 2007); *AtAGP6* and *AtAGP11* are involved in pollen tube growth (Levitin et al., 2008); Over expression of the FLA3 led to short siliques with low seed set due to the reduced stamen filament, suggesting that the *FLA3* gene is involved in microspore development and pollen intine formation (Li et al., 2010). Next, we also investigated the expression patterns of nine *ZmPCs* detected in maize seedlings subjected to salt and drought treatments by qRT-PCR. The primers were listed in **Table 3**. The analysis revealed that these genes are differently expressed under salt and drought conditions (**Figure 6**). Among the nine detected *ZmPC* genes, all members were down-regulated under salt treatment. And five members (*ZmUC19, ZmSC2, ZmENODL10, ZmUC22*, and *ZmENODL13*) were down-regulated under drought treatment. Some rice *PC* genes (*OsENODL19, OsENODL12, OsUCL17, OsUCL20*, OsUCL7, *OsUCL8*, and *OsUCL18*) have been investigated to be down-regulated by drought and/or salt stresses (Ma et al., 2011). Interestingly, we also found that *ZmUC16/21* were significantly up-regulated after drought treatment, suggesting that these *ZmPCs* are more likely to play key roles in maize drought response. An increasing number of evidence has suggested that PCs may also function in stress responses. Previous studies reported that some PCs, such as, *OsUCL23/26/27* (Ma et al., 2011), *BrUCL6/16* (Li et al., 2013), were up-regulated under drought or salt stresses. Moreover, over-expression of AtBCB/AtSC3 could confer aluminum resistance in *Arabidopsis* (Ezaki et al., 2001, 2005). And BcBCP1 can enhance tolerance to osmotic stress when over-expressed in tobacco (Wu et al., 2011). The differential expression profiles of different *PC* family genes may imply diverse roles of plant response to stress. On the other hand, *PC* genes which are up-regulated during several abiotic stresses are likely to be required for enhancing resistance to stress. Therefore, PCs can function in developmental processes and stress responses.

**Table 3:** Primers used in this study.

Primer names	Primer sequences (5′–3′)
ZmUC10-F	GACCACCACAACACCGTACA
ZmUC10-R	GCTAGCTGGACGATGACACA
ZmUC16-F	TGAAGATGCAGGTGCAAGTC
ZmUC16-R	AACGGAAAGTCTGCTTCGAC
ZmUC19-F	AACAACATCTCCGCCTTCC
ZmUC19-R	GTGCAGCAGAAGCAGCAGTA
ZmSC2-F	AAGAACTTCCGTGTCGGAGA
ZmSC2-R	GAGTTGGTGCAGCTGTCGTA
ZmUC21-F	GTTCGTGTACCCCAAGGAGA
ZmUC21-R	GCTTGTTGCAGATGAACCAC
ZmENODL10-F	CGACGACCCCTACAACAACT
ZmENODL10-R	CTTGTTGGATCGTGACATGG
ZmUC22-F	GACGTGCTCGTGTTCAGCTA
ZmUC22-R	GAAGTAGTGCGTGCCTCTGC
ZmENODL13-F	GCGTCGTCTTCTTCCTTGTC
ZmENODL13-R	GGTCGAGAACGAACTTGGTG
ZmENODL15-F	GAAGACCAGCTTCCAGATCG
ZmENODL15-R	GCTTGTCGTAGGAGGAGGTG
Actin1-F	GCTGAGCGGGAGATTGTCA
Actin1-R	CTTCCTGATATCAACATCA

## Summary

A comparative genomic analysis of the *PC* gene family in plants was provided in this study. This gene family had an expansion process in the course of plant evolution. A structural analysis of PCs indicated that 292 PCs contained N-terminal secretion signals and 217 PCs were expected to have GPI-anchor signals. Moreover, 281 PCs had putative arabinogalactan glycomodules and might be AGPs. The gene organization and motif composition are highly conserved in each clade, indicative of functional conservation. Most *PC* genes may be originated from the tandem and segmental duplications. In addition, expression profiles of the maize *PC* genes also provided better understanding in possible functional divergence. The results provide a base for further functional and evolutionary study of the *PC* gene family in plants.

## Author Contributions

JC designed, supervised, and carried out parts of the experiments and wrote the manuscript. XL, YV, and LD performed the experiments. XL, YV, and LD provided material, and helped in data analysis and writing. All authors read and approved the manuscript.

## Conflict of Interest Statement

The authors declare that the research was conducted in the absence of any commercial or financial relationships that could be construed as a potential conflict of interest.

## Footnotes

1 http://www.phytozome.net

2 http://www.cbs.dtu.dk/services/NetNGlyc/

3 http://meme.sdsc.edu

4 http://www.dyogen.ens.fr/genomicus/

5 http://www.ncbi.nlm.nih.gov/genome

6 http://meme.sdsc.edu

## Supplementary Material

Figure S1 **Motif composition of PC proteins and exon–intron organization of PCLD in plants.** Conserved motif distribution of the PC proteins is displayed. Positions of the 0, 1, and 2 phase intron were shown with blue, bright green, and red vertical lines, respectively.
