# Supplementary figures and images for: Comparative analysis of the phytocyanin gene family in 10 plant species: a focus on Zea mays
Source: Front Plant Sci. 2015 Jul 13;6:515. doi: 10.3389/fpls.2015.00515 (PMC4499708; doi:10.3389/fpls.2015.00515)

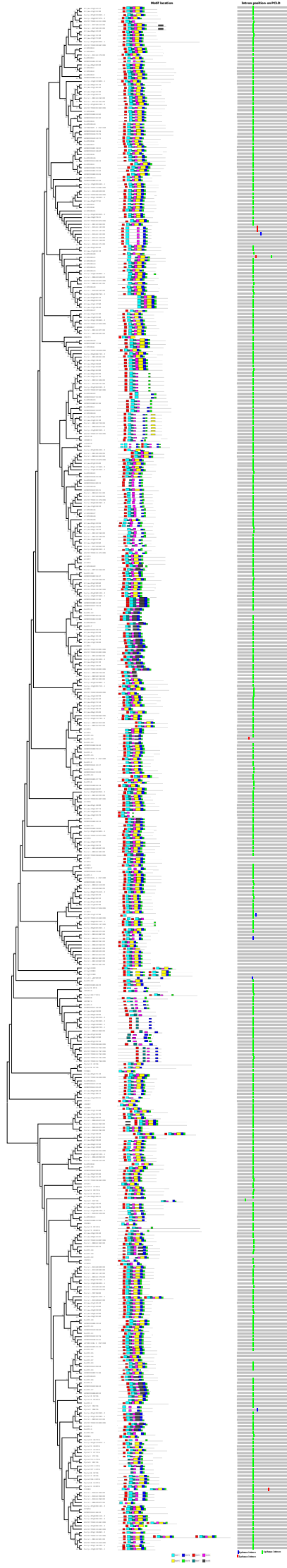

Supplement: Figure S1 — Motif composition of PC proteins and exon–intron organization of PCLD in plants. Conserved motif distribution of the PC proteins is displayed. Positions of the 0, 1, and 2 phase intron were shown with blue, bright green, and red vertical lines, respectively. [file Data_Sheet_1.PDF]
