# Supplementary material for: Comparative analysis of the phytocyanin gene family in 10 plant species: a focus on Zea mays
Source: Front Plant Sci. 2015 Jul 13;6:515. doi: 10.3389/fpls.2015.00515 (PMC4499708; doi:10.3389/fpls.2015.00515)
Supplement: Supplementary file 2 [file Table_1.DOC]

**Table S1. Protein backbones of PCs in eight plant species except for *Arabidopsis* and rice.**

| Organism | Name | ID | Type | Protein backbones^a^ |
| --- | --- | --- | --- | --- |
| *Chlamydomonas reinhardtii* | CrENODL1 | Cre14.g629050 | II | MRAFIAFVLALAVFAAVQVQGAQYNVTWADDGSPIDLSVNCSDVVQFSWLPRHELEQSRGYGCGKPTIRNFGEGVNIVAPLVFANPGTYTFACNIEDHCTVGKQLAKVHVGACSTPKNTINWAYRRDNASYAALQLTCGETLTFSWTSGRHDVAEVEKPDCTSPTIAYYGNGTRFDNGTYFPPASTGSVSFTYTKAGRHHYKCDVPSHCSVGRMLLTVDVTCPTKSASPPPRRRSPPPRKWVHWG |
| *Physcomitrella patens* | PpUC1 | Pp1s10_52V6 | II | MAKVVLSVLLFVVVLAMASAAYADNQPAGREYQVKWDFPTGDKYYSAVQGKTYYVGDSLKFTYMQEMHNVVKVGSFEDFNQCTMTKPLSPEFADGATSMPLDKPGVHYFICSIPGHCSDGMKIKVLAINRPRKLM |
| *Physcomitrella patens* | PpENODL1 | Pp1s13_43V6 | II | MAPESQLGGPSRSFTLLLLVSVIAVSYGEASTHIVGGDRKWDFPPNSSSSSWYDDWANNNTFRVGDQLVFNYTSGLHNVAELATGNDYDMCNLSAIITTFGSGVDVVTLDRPGWHYYVCAVLGHCATRYLKVKIYVNASDAGSASISSSVGAPGGGSNLSAIVGAIIGTVVVMLVAAGAVWYNRRRKRFSSLHGSRRSGLTARKSPLIPFESLGADGPRVFTFKELAAATKNFSRTELLGRGGFGSVYKGVLRDKSMVAVKSIAKDSRQGEIEFLAEVSIIGKIRHRNLVRLRGWCAEKEKLLVVYDYMPNGSLDKWIMPAEEGETNPVLAWNARYNILSGLSGALAYLHEEWQQCILHRDVKPSNILLDDKFNAYLGDFGMARLIDHNKVAYSTVVAGTMGYLAPELPHTRKATPKTDVFSFGVLALEVTCGRRAFDPNRPHAEVYLLDWVWTMHQNNQLRKCVDPRLGEDVDVMQSRLVLHIALLACHPDPASRPSMRFVRQVLYGDLSLPTIPPSRPVISYSWSSTIQPTQECISIEVQHPENTDESVRKPDNSPAA |
| *Physcomitrella patens* | PpUC2 | Pp1s13_48V6 | I | MRQGRERGLFGAVILVLAMLTFLGSAYAAQYIVGGDVPKWNYLHATSKPSFYQDWADTIAFKTGDTLLFSYDNSTHSVYRLGTASDFTSCNLGTVIPGGSFHSGQDIVFLSTPDTYYFVCGAPSHCKQGMKFALSATGSPISAPPPPPVGSDAAAPGSQASLTVTRPGSVSLSICISVAAFVSIFL |
| *Physcomitrella patens* | PpENODL2 | Pp1s15_221V6 | IX | MLMSVNLIADRASSVHKGLETEAGYWDWHGIYDGLSHAHAKSVKVGGHLGWTNFNTATGLVQDYAGWAASQSLVRDDIIEFNFRPGARVLFQVDSQAALDACNFTGVVPIFDQNSSSPVHVTLTASGVSFFGYGGMTSKGVSNCEPGQRFAVSVHDYAMTTGRRYLANAPAQEKNNNTAIIAVVIGVVVVLGAIGACYCYKQRKGTGRKLLRKRASNSNLQEVISSIAQGHGSVTVFTLRELEKATENFGEHLVLGLGGFGTVYKGTLRNGMVHVAIKMSNAISKTGKKQLLNEIAILSKTNHPNLVKLFGCCIETEVPILVYEYIPNGNLFEHLHRLRFGVNLNWKKRLQIATEAAEAIAYLHFAAQPPIYHRDVKSGNILLDNTFSVKVADFGISRLTNPEKTHVSTAVQGTPGYLDPEYFHSYHLTDKSDVYSFGVVLLELITSQKPLDYTRGDEHSLAAYALPIIREGNLDLIVDPQLKESMEEFQESIPTIQRVAQVAINCLADKRKDRPTMKTVAAALQDINKTYLNHNNGGGTEFDD |
| *Physcomitrella patens* | PpENODL3 | Pp1s189_75V6 | IX | MRAMHPDLTWGAMKVSPEQWADCPTPQLMMPHIPHPLQIYHHNNHHCNQTSSRLVAGLLNAAPQLVAAAASKFVPHRAASLTTLQGKPVLLGALASVGLLRVLYFCSGGDTSVIALECMMVCDPRCQWNQCKCVRWGKYHMEASGRYCETRDCSEPPGISSAYSCRRLLKLETEAITLEQIINLSMALTKGFEVDFAIAAVIVFMVCTCHAKSVDVGGTAGWASYDSSQTTAPNYEAWASSQKFYVGDSLVFKFAAGVHNVWQMKSQATYQNCDFDGATLLDEGNSGYYSWKATQPGVYYFSCDKGAEGVGTHCNFNQKLAIMVSKSASAPTPLPPPSPLPSTSPPPSIAPPPHPNITASSPSQPPPPSSRPPSVSPEPHSFTTPAPATPPNSVIPTPSPTPSYSSPPSKAPTPWHHAAPVPVPAPVPVPVPMSTPPPASTTTPAPSPSIPPSTSPISTPQASTPSDSSTPDPGLPEVPGGEPTALVTPPQNAAGALRRAQLGCSVLLQAITLLSVLSK |
| *Physcomitrella patens* | Unknown | Pp1s2_394V6 | VII | MSSFLLKNWYWAFPRLHSTANIDYDQWLSNYKVKIGDTVEFGNNDSTDHTVVVVDKEGYDTCGKSGVKYDTTTVSLGYLLQRTYKESGDYYIICDISAHCLQGQKVYIQVLNEDGTINTTDTPKDPERPSAAPHQMPGFTWTSKLVIVVVSAFSLMMAAAASM |
| *Physcomitrella patens* | Unknown | Pp1s2_396V6 | VII | FGNNDSTDHTVVVVDKEGYDTCGKSGVKYDTTTVSLGYLLQRTYKESGDYYIICDISAHCLQGQKVYIQVLNEDGTINTTDTPKDPERPSAAPHQMPGFTWTSKLVIVVVSAFSLMMAAAASM |
| *Physcomitrella patens* | Unknown | Pp1s2_537V6 | VII | MSPKQRHGAAATGFEIARSIQVNADQRHRDGPGDPHKALPAVFASHFSFRYLDARVGVAHSTANERTPTCSGFWHSPEAQINKAGIAAFSLRDLLSTFAYKTGSSGKASDGYASWISAASVTIPVFPVILDCESMQIQFLSVAFYLFQHTFHVVRFRIPKASSPSPRVRLLRSDLSEHETVWRAARALTGYCGGSNSASICFSRLLRSEDTSGSLLESYIKIYSQTETSRVQMASQLRRKSSIPIIVQVGLALAVCVNMGLLHYVEGANFTVGAGYVESSTGSTYWTNTIGGLLRPEILLSSYQRWTNSVLVSVGDTLIFEYERGTHTVFLARNKTAFTKCDFDGAVQVPDGSPTTYTIKPTDSTLFFVCTLPGHCDSGQKVEIVPLGTTPQPPPQPTPTSNALRQSRMKPATVVTLLLTSYLVFFAHCV |
| *Physcomitrella patens* | PpSC1 | Pp1s227_44V6 | I | MAQGRCSAMLAVALLVVSAAVVAQAVTYDVPRWTVPAAANADVYTTWAANVSNFLKPGDVLVFQYSAAAHNVLTLATKANYDNCVKTSPLNTTSTGNDALVVKAGGNYFICGIPTHCESGQKVAVNVSAATGTPETPGTPAAPGTPAPQGPSSATSLTVRQTFAAVSVALSASLIIW |
| *Physcomitrella patens* | PpUC3 | Pp1s23_198V6 | I | MAQGRGNAVGVLFVASLLAFAHTVVGVDHVIGGTNKWDYPPGTDTNYYATWSAKHNFVVGDSAVFNYVATQHNVQVVTANEYRSCAQSNGQTYMTGKDSIPLTTAGKYYFICSVISHCEMGMKIMIDVKAAAAAPPPATTTPPTMSPLPPTEAPAAAPVLPPPPSTATSVPACLSLVAVMAASAFGVLFLC |
| *Physcomitrella patens* | PpENODL4 | Pp1s256_48V6 | I | MAAMRQGRGSAFSHIAVLVVSLSMFLKGTLAAQYVVGGELSKWSFLHSNSKLSFYNDWAKNITLKTGDSLQYIACTLPKTPVGKWVSGRDSVFISNAGTYYFICGTPAHCAQGMKFTIVATGAFVAAPPPPPPVAGAPGPANHTAPTTTFSSAVSLLAALSLSATIAVAM |
| *Physcomitrella patens* | PpENODL5 | Pp1s274_57V6 | II | MAKGKGIDSAGHEIMLTISLILGLLAVSPAAQAVDHVARGQTVFNYTVGGHDVVRYSRKTEFDNCARVTLQRWTSGADRIVLNSTGFFYCICSIPGHCETGGGMKLSIHVTTTRAVAPTPPHNGTLVAPSASPTTPARSAANPSPNQSISGLVAAAILPGAAFVV |
| *Physcomitrella patens* | PpENODL6 | Pp1s31_207V6 | IX | MGTQKRMAKTVKRSNANMNSAAAAVAVLMLVASVTDTASAKEYTVGGTTGWDYAPTTSFYSEWSNKLRIVPGDKIVFKYMPTAHNVQEVTEADYAACNSMNPITEYQSGNDIVTLPKQGTHYYICGVLGHCTEGGMRMKVTVVADDSLNSAAPAGSLPLPQASTPQTGASSTSGLARRGGDHANSPAIPPISTAGHSAAQISAKPKSLSGMAVLWSVGAAALEFIVGVGINEVNVHGHKRLHNVS |
| *Physcomitrella patens* | PpSC2 | Pp1s333_41V6 | I | MAQGRCSAVLVVSLLLVSSAAVVHGVDHNVGGTTKWIKPDGSTVPENFYQKWASEEKFTAGDNLVFVYNPAGHSVLEVSATDYAACSTTTPIKRYATGNDIISVPAGPSYWICGIPSHCPAGQKFNITAAAGSSGPSSSVGTGNAVSILHVVAALAVALIASMSM |
| *Physcomitrella patens* | PpUC4 | Pp1s4_171V6 | I | MAQERSSARIFIVAVGLLAAVHAVAAKDINVGGTRGWDYAPPSDAAYYDTWASKETFTAGDNLVFSYTPGAHDVQVVSATEYNACSMSTGKKYLSGGDSVSLPTPGTYYFVCSFPSHCDMGMKMKITVKAAGGVPPVVAPVTPPVTPPVRAPTRAPLVAPVPAPVVKAPAPAPVIKAPTPGPALAPVPSPTDAPTPSENPSTAPTPGSSIAPGPELPPPPPSAAVSIHQCLSFVGAVAVVAFCVLRF |
| *Physcomitrella patens* | PpPLC1 | Pp1s41_159V6 | VI | MTSYSMVVTVMLAVVLLAARDTFAVEHKVGGALGWNYPPNQGAEYFANWAAQNAFHVGDSLSFEYIAGTHNVVQVDRSDYDACTVTRPMQLYNANKVIVNLPRPGTYYYICGIKGHCDYGMKVALTVT |
| *Physcomitrella patens* | PpENODL7 | Pp1s42_204V6 | IX | MVYDGACTEAHVSLQFPSKKTHSCEEMEVAAAAAAATAVPVCVAVVKTLQWAITDTSTAADNSRDARRIAMMGKPYGTMSKVASVVFMLIASMACAVTAKEFTVGDTTGWDFAPNSSFYNDWANGLKFVPGDKIVFKYIPSDHNVQEVTESDYVSCSSLNPLAEYESGNDIVTLPKPGTHYYICGFLGHCDQGGMRMKITVRGAYAPQSVHGGATSPTGDLPSPMTGMDPGLPSADTATVLPQGNHERALTPAAVPVTVPGHSAACSSRELAIFKRRRPFFILMGGAAAIFPLLWK |
| *Physcomitrella patens* | PpUC5 | Pp1s44_258V6 | II | MAMAVWVVILVALSVASLADAGAQPVGKEYEVKWDFPTGDKFFSAIQGKTYLVGDTLKFKYMQELHNVVKVGTFDDFNQCTTTKVLSPEFADGDTSMLLDKPGVHYFICSIPGHCSDGMKIKVLAVNRPRKLM |
| *Physcomitrella patens* | PpSC3 | Pp1s46_32V6 | I | MTMGRGSAMLAVAIFLVSAAMGVRGATYTLPGSWVVPTENADIYNVWAQNVSSYLVEGDVIIFQYVAAAHDVVTLGTQAEYDGCDSTTPLNRTQTGNDAIIVKAGINLYICGRNGHCQAGQKVSVTASAANIITPTISPVASIPALSPPLSAPTISPASGAALPAIYTITPAAAPHSAGSVTVPQAVAAATVAAAACFALF |
| *Physcomitrella patens* | PpSC4 | Pp1s49_6V6 | II | MRGNSVRLLLAVLLLAVSVSECVEIVVGGTKGWTTGFDYDAWAASQNFRPRVGDSLVFLNPDSEYHTVSLLDSLDAYQRCTLGGIQPNATHPARPGENYTMIIPESLSGKMLYAVCTVSGHCLEGQKISATVLPAAVGIPLSSPPASPPELSDADMNQYICWTTAILLIVACFWNSG |
| *Physcomitrella patens* | PpUC6 | Pp1s56_142V6 | I | MAAMRQGSGDVLFCAAVLLVAVSSFLEGAVAVQHVVGGEVSKWSFLHANNKASFYNDWAQNVTLKTGDSLCLLTRLVSRLTVFQYNNATHSVLQLATEAEFTACTVPKTPVDKWVTGNDAVFISKAGTYYFICGTPVHCNQGMKFTIAATGDFVAAPPRPVGAPGPGQTANHAAPTAVFSGAPSLLLSLLLIATTFVTL |
| *Physcomitrella patens* | PpENODL8 | Pp1s56_57V6 | II | MAQRLRVLQTLMLHLFLDFITVHHGAATMHTVGGETQRWDYPPRDFWYEEVWAKSIIFRVGDQLVFKYKQGIHNVAVVASQAGYEMCNLSNVINTYNSGTETMDLNRTGWYYYFCAVQEHCSNRNLKMKVYVNESLADGTPSGGGSSKSIVIIVVAVIIAIVVILVAVGAVCYYRRRKGLDDFPCSHRSGNSGPRSSTSIPIEILGKDGPREFTFRELAAATKNFSRTELLGRGGFGSVYRGTLRDKSLVAVKCIAKDSQQGESEFLAEVLIIGKIRHRNLVPLRGWCAQREKLLVVYDHMSNGSLDKWIIPSQDGKVKPALQWNTRYSILSGVSAALSYLHEEWQQCILHRDVKPSNILLDDKFNAYLGDFGMARLVDHNKIAHSTIVAGTMGYLAPELPHTHKATTKTDVFSFGVLALEVVCGRRAFDPKLPHDEVYLLDWVWSMHQCDQLRSCVDPRLDDDFDDMQTRVVLHIGLLACHPDPGSRPSMRFIRQVLDGDLSIPQIPGNRPVISYSWGSGSQSTQVSNSMDQSTDGISQSDKAPLTYPR |
| *Physcomitrella patens* | PpPLC2 | Pp1s57_155V6 | VII | MSQGSSGVFSPRSLHYHMAQGRSSAMAVLVIVAVLAFSQAVTAKDYNVGGTLNWDFPPGTDVGYYDTWSSQQKFVAGDSLTFTFDPRAHDVQIVTESEYTNCAMSSGKKYTSGKDAIPLTKPGKYYFICSFMGHCAMGMKMKVVVATGSSTPVTPPTSPATPPVTPPTASTPPPAVSSPPPAVSTPPLAVPTPPPAVPTPPPATVPAPVVAPVTPPVSAPVPPPVTSSPASTVAPVPAPAKAPTPPKEPSASPSPASSTPEPASAPVPNQSAAFLIRACLSSAGAAAIVSIGALLF |
| *Physcomitrella patens* | PpENODL9 | Pp1s57_170V6 | I | MGLHRSILELVVVLACSALLLPVAMAVEYVVGGPGGWTSVPTASHYTDWATEKHFVTGDKLNFRYDPTEYNLQQVSSNDYSTCNTLHPIRQYQSGNDVVKLRTAGTYYYISGFAGDCNEGGMLMKVVVAQSLGPASAGESPPPLNHSPPPQNHPPPAASNSPPPANIPPPPHHSPPPPPPPPHSSPPPTPASSPPPPPPTDISPPSPPNQLTNPPPPPPPAPANKTSHAHPAKTAGAPSASTLLPPTPSTDTAPSPSESTPDAANPRRVGTAMVLAVSVLVTAALL |
| *Physcomitrella patens* | PpENODL10 | Pp1s72_106V6 | II | MGAMKFTAARCVRVTLNQLIIVLSLQFSSSQAQAKSVRVGGEQGWTSIDSATGLVRDYADWAASQVLFVQDSLEFHFTPGACSLFRFQSQVALDGCDFTNGVFLFDLNSTSPVKVTLTESVVHYLGCGRKTASGVSHCQLGQSFAVVVHSSRARRQLADLSPGPGPDEASTKSSSNNTTTIAAVICVLLIVIGGILVCYCMRNRRIVQNTTLRKRVSNTDLQEVISNITHGNAAVTVFSLKELEKATENFGEHLVLGLGGFGTVYKGTLRNGMVHVAIKVSNSASKSGKKQLMNEISILSQTSHPNLVKLFGCCVETEVPILVYEYIPNGNLFEHLHRLRFGVNLNWAKRLQIASETADALAYLHFAAQPPIYHRDVKSANILLSNTFSVKVADFGISRLTSPEKTHVSTAVQGTPGYLDPEYFHSYHLTDKSDVYSFGVVLMELITSQKPLDYHRGDEHSLAAYAIPIIKEGNIDMIIDPQLKEPRDEYEKSLPIIQCVAEVAMDCLAEKRKDRPTMRMVADDLQSIKSFARRNVSGRNV |
| *Physcomitrella patens* | PpUC7 | Pp1s77_277V6 | I | MAQGRGSAMVIVVVSALLVLAHTVVAKDFTVGGTQGWGFPPGTQTDYYDTWSSQQTFEAGDKLIFTYSPVQHDVQTVTVSEYSGCTPSQGLKYTTGKDTIALSAPGTYYFYCSIVGHCDQGMKMKVVVKAATAAAPAPLATPPTGSTGSTGSTATALSISHRSIAAAAAVASLIAAFLIQV |
| *Physcomitrella patens* | PpENODL11 | Pp1s8_201V6 | I | MVGCKGRGSEIHSASIAIVLASTLVAIVSVAEAVDHVVGGTRQWDFAPQTDKSYYQKWADNSTFNVGDVLVFNYAAGSHDVAQYDTKAKFDRCNGTTVNIWTTGSDRITLTSAGTFYYVCSFLTHCSTAAGGMKLAVTTASAVGSPPAPSNAPISPTPPPPSGASLSSSLPMAGLVAAAFIVGCVILV |
| *Physcomitrella patens* | PpENODL12 | Pp1s83_167V6 | I | MGSGARSSFLLVAPVLCAAMLLGLFVDHCACQTPQIYTLGGAAGWTDVANKKYTAEMAAVPFKAGDTILFNNNDTKVHDVLQVYTQEDYDTCNTNGQMGWTLNPGDTHGVQLIDPAYRVWFFDTVYCLTGQKVEFTVRNSDGTVVPLSTGADPPSLNAPVPEPVDPNVPPPPVGIAGGPAPNAAGTNGRVTWISLVAIASVSYFATFVL |
| *Selaginella moellendorffii* | unknown | 19117 | IX | ATRYIVGDEVGWSDPSMSNVSYADWALKHRFHVGDSLVFKYPSDAHTVLKVNRQDFEACHNSNSMASYKDGESIVHLSSAGPHWFICGETSHCNQGQKFGIMV |
| *Selaginella moellendorffii* | SmPLC1 | 19213 | IX | AALYKVGDNLGWNLNVNYTQWAAKYPFALGDSVVFVFSGSHSVLMVNEIDYVLCNIHNPVQSLLSGRAITLAARKNFFICGIPGHCITGMKVAIYASVASSS |
| *Selaginella moellendorffii* | SmENODL1 | 19307 | IX | VGESAGWMIPSAAVNYSAWALKHNYHPGDTLLFNYQQQGDSVLEVNRADFMNCIKTNPINHHSDGKTLIRISRPGPHWFISGVPGHCEQGQKFGIMVTPAS |
| *Selaginella moellendorffii* | SmPLC2 | 24493 | VIII | LAMAVDYQVAGAAPGWSIQNGYTEWAATNQFRVGDTLTFTYTGNHNVLEVSRAAYDSCDASQPIQSYLTPSPIQVTLTTSGEHWFICGVPGHCGGGMRVPINVLEATSGAPGGAPAVPSTPTGVAFPPPRPNSAAGNSPVLSAGLAAAAAGALAIFTPWNLSMKKEDYKAWAATQTFLTGQTLQFKYESGHSLLALATQEAYNNCDLSNPVKTFTEPNPIVTLGAPGKKFYVCGVGNHCNAGMKVIINVVSSADAAAP |
| *Selaginella moellendorffii* | unknown | 27471 | IX | VVGGSAGWTLPSFGHVNYTQWTLGNRYHLGDTLVFNYSKDFHNVLAVSKADFIACSTANPIATFQDGHTIINLDTTGPHFYVCGVPGHCGQGQKLLVVV |
| *Selaginella moellendorffii* | SmSC1 | 27978 | II | FSFLVALLLATLGAVQATEFVVGGATQWIMPPNGDDDVYENWSKQQNVRVNDTLRFKYNSQRHDVLEVSEDDYDRCSSASPIQSFNNGDTSIAMTRPGSWYFLCGFPNHCQGGQKLSIDV |
| *Selaginella moellendorffii* | SmPLC3 | 29265 | IX | FTVGDSNGWTFQVNYTQWASSQTFRVGDILVFPYTSIHDVREVSQADYDSCDGSNAVTTYATASPIRVTLSRPGAHWFLCGIPGHCAAGMRVPINV |
| *Selaginella moellendorffii* | SmENODL2 | 37976 | IX | AIASSLLLSDRANARAYLVGDDRHWDLGVDYAQWASKYSFVMGQDSLVFIYTPPRHSVLQVTQGDFDGCNINNPIATIPPNSSFAIASPKAYFICGVPGHCVSNLKLAITATTTAPSRNITATAPEFSSSPPPPS |
| *Selaginella moellendorffii* | SmENODL3 | 39173 | IX | SQAARYTVGDSDGWKPDVNYTSWALKQKFYPGDYLVFNYPEGQDTVLEVNRAGYESCASSNPINHHNDGKSVLRLTRPGTHYYI |
| *Selaginella moellendorffii* | SmPLC4 | 405136 | IX | MGASKQERGNAAVWAIAMAILLARAAIASAYMIYRVGDDDGWTANAPGIDYTKWASQKAFQVGDMLVFAYSGANHTVLQTSSQDAFDACNTGVEDAKIWSADGSSSSNVMLTTPGRTYFLCTADDGGHCRAGMKFGIDVTGTAPSAGSDGLVAPTGSPPVDSSWNSPGPVTDFSPPFLTPDPSSSSSTGSAIAPPGSITTPSENSNSPLYSRATPTRMIGDGFEVAILVFAAMVL |
| *Selaginella moellendorffii* | unknown | 405234 | I | MGRSTNSLTVCFCLLLVLFLHRRAMCVVITVGDSLGWTNFDLSTQRVPDYAAWAASQPVASGDSVVFRYAPGFHNVAMLPSKADFDNCNFAKATMLDTGSSGNFTWIAPEKAGAYYFACGFSVEGQGTHCDGGQKVTISVGVLAAAPPLALSPTPAGLVLAPGLPPSSPSSSPSPGGGPTSPAASPGMGLVPAIAPATSDSPSSSMAPSGSSGASAAVPIEGSEQDNTSGGDSLATWWRLIVVSTVVALRLL |
| *Selaginella moellendorffii* | SmUC1 | 407674 | II | MARFLVSGFAIALLLAAVANVCSAKSVMVGGRNQWSLGTNYASWAAGAGPFRIGDTLVFSYGGGRAGKAAAPHNVFLMKDQAHYRNCDFSGAVLLADPSKGTPGYKFTLKQKKAHYFACGVGNGFHCQSGMKFAVSPR |
| *Selaginella moellendorffii* | SmENODL4 | 408831 | I | MRNSSPLLLLLVPLWIQIASQLVGAYTTYIVGGDTGWTIPTASNTIVNYTAWASSLTASLGDSLVFRYDPSHTVVQTNNLTTYQSCDATADDETLKIWSSSGSSTVMLTTTGTTYFFCSADDGSHCRDSGMRFAIQVSFGQGLPATPKAAPSPQDGSDDGDDLNFPNFDKFAPPPPPPSPPADSSATAPASLRGFQLAWVLAAALVLGR |
| *Selaginella moellendorffii* | SmSC2 | 409386 | I | MNALFLPGIVLMWSLLPPCSSKQVLKVGGEIGWTQFNIFSGQTPDYAAWASKTTLVPGDDIDLAVFLFFPQLHNIHMFRNHSEYVNCNFTESTLLDEGLSGVYTWTAPAPGTYYFGCSKDYYSHCQLGQKFAITVVEQSSLIHLPPAGSPAAAAASPSDDSSPADNGCATASSDDDDDDTSHDSDSSCSPWVDSPPDDTPVLSPVSARHHPDASVNPRRVPDFGTNTKRRPPPRSSSAREFHARAYAIRGLIVFVLNLVLLA |
| *Selaginella moellendorffii* | SmUC2 | 422957 | I | MAATAQGSGSAALLLTVLAALYCSVLSATIRIDWRQGVDYSSWSTTNTVRTGDTVTFSWVGSHTADVVSEADWRSCTPNSLQSVANGGGLTVGTGTTYVICTVAGHCAGGMKVAITAASTPSTPTTPAPPTTPTTPTTPTTPTTPTTPTTPTTPTNEPSAASGLQVGTALVVASVALLL |
| *Selaginella moellendorffii* | SmENODL5 | 443452 | III | MKFLVITCCCIAIQILGTAAEGTDYYVGDWTTGVNFTQWSQGRVFHAGDILIFTVSASDTILRVPKSVYDDCKWDLRFPKIFPHPGNTTWNETVVPWVGENYYVSSVQDNCNAGKKFMVSVESPPVYTPTEYTVGDDRGWAPGVDYSQWTANKNFYFGDSFRFLFNASQHSVVEVWEPGYQLCNESYFVPVLGLASRQSDGRTLLKVVPPLGMRYYTSANGNDCQSGLKMELEIKPQYEAFAPSPSPEEAFSPTGGVAGQETSAGYRSMTSLGSGAAIVVLLLLAIA |
| *Selaginella moellendorffii* | SmPLC5 | 69095 | IX | LLLVDFACAATYIVGDSQGWDLNVNYAAWAGKKKFRAGDVLIFTYTQMHSVVEVSQADFATCTITPISTYMSGNDSVTLSSTKSKQFFICGTGGHCGSGMALQVDI |
| *Selaginella moellendorffii* | SmENODL6 | 69294 | IX | FMLSVQSPPVYTPREYTVGDDQGWAPGVNYSQWTANKNFYFGDSFRFLFNASEHSVVEVWEPGYQLCNESYFLPVLGLPTRQDDGRTLFKVVPPQGMHYYTSGNGNDCQSGLKMALEIK |
| *Selaginella moellendorffii* | SmPLC6 | 72991 | IX | YTEWAARNQFRVGDTLKFKYESGHSLLALATQEAYNNCDLSNPVKTFTEPNPIVTLGAPGKKFYVCGVGNHCNAGMKVIINVVSSADA |
| *Selaginella moellendorffii* | SmENODL7 | 76299 | I | MFFVASTGSLLASITVLAVFAAIVSAGIQHNVGDKAGWKLPSLAKINYTDWASQYSFQVEDTLHFRYDQGTESVLQVSLADYVSCSNSKPLATYDDGDTVVYLLRDGWYWFISGVPSHCNLGQKFSIRVQPLSHGSYQDHAPSAAEPSTATAQGFSGGSRRENPVAIPVSALPSSSAGFATPVFPALVYVLPIVLVA |
| *Populus trichocarpa* | PtENODL1 | Potri.001G043600 | VII | MHSMVIKPGLRSNNLLIYSLAQKVAENQTSHCEMVSSSARLIFFCFFILFSAIATTATDHIVGANKGWNPGINYTHWANNHTFYVGDLISFRYQKTQYNVFEVNQTGYDNCTTEGAVGNWTSGKDFIPLNKAKRYYFIGGNGQCFNGMKVTILVHPLPPPPTSATMAANVKSSDSAAPLVFHKGLVGLRALVLAVASIWFGSGWI |
| *Populus trichocarpa* | PtUC1 | Potri.001G080700 | I | MAKLVLVYSLVVLGLALTCNAATYMVGDNSGWDISTDLDTWAQSKTFVVGDLLSFQYSSSHSLEEVKKEDFDSCNTTNVARTFTNGNTTVPLTEPGTRYFVCGNQLHCLGGMKLQVNVEDNQANPPIGAPQAQPAGGTLTQPSSKSNNPASVIPTSAGSVYGGRDCIVMAFLGFVATMFWVVRV |
| *Populus trichocarpa* | PtENODL2 | Potri.001G085100 | I | MVNLRSPRFLVLYAFQFLVLVQIQVSCYQYKVGDLDAWGIPTSANPQVYTYWSKYHTLKIGDSLLFLYPPSQDSVIQVTRENYNSCNLTDPILYMNNGNSLFNITAYGDFYFTSGVQGHCQKKQKLHISVPGNGSASAYSPSYGPSALPDSAPSYPTVFGSIPLPPSSSPLNRFSILLSFIIGAGIWAIIM |
| *Populus trichocarpa* | PtENODL3 | Potri.001G114200 | VI | MASSRMFMIIAIVAVFLPSILATEHVGKTFYVGDTLVFKYTAGAHNVLRVDGTGFQECKAADDIVPLTTGNDVIPLSTPGNKWLHLWCGQTL |
| *Populus trichocarpa* | PtENODL4 | Potri.001G187700 | I | MKITKMASLAVLFCTCFVIATGLGNAEKVFKVGDEFGWQEPGQNSSAVYTQWATRNRFQVGDSLSFEYNNDSVIEVDKWGYYHCDGSKPIVAFNNGHGVFKLDRPGPFYFISGTPNHCMGGQRLLIEVMGLHHHSPLTATPPAGQLAPSPQPSSGVFVSVTLGSLSTLLMGTLIALLWCLP |
| *Populus trichocarpa* | PtSC1 | Potri.001G192100 | I | MFNIGVTFGFAMMVLFQRSVAQTVYVVGDNDGWTVPQAGAQAYITWASGKNFMVGDTLTFNFTTNNHDVLRVQKESFDACTSSNSIGDVISTGPVNITLDSTGEHYYICTIGRHCQFGQKLAITVSSRTTGASPPSTTPRPPPPRSPTATPSPSSNNTSDGCAPTPAPSPTSSMIPESLPTIPSPPGSSSSNVLASFMMTMLAAIVGLVF |
| *Populus trichocarpa* | PtUC2 | Potri.001G209300 | II | MVQGRGSAMVATVAVMLCMLLLHFDMAHAATYTVGGPGGWTFNVSGWPKGKSFKAGDILVFNYSTAAHNVVAVNKAGYSSCTSPRGAKVYTSGKDQIKLVKGQNFFICSFAGHCQSGMKIAVNAA |
| *Populus trichocarpa* | PtENODL5 | Potri.001G219800 | II | MEVFSLKKMLLWLITVVNILGSTAECREPVLHRVGGGKYTWAPNMNFTAWAMHEEFYVGDWLYFGFDKTRYSVLEVNKINYNNCNDKNCIANITRGGRDVFNLTEARPYYFLSGRGYCFKGMKVAVHAQYPPPDPAPLVVRNVCPSKSASHGLAMLLALFTSYAVMG |
| *Populus trichocarpa* | PtENODL6 | Potri.001G219900 | I | MESLRKMLVVLMTMVVTVRMVNASLVYVGGGKETWRSNVNFSEWSARQNIYVGDWLYFGFDKKLYNVLEVNKTGYEGCHDVGFIKNITRGGRDVFQVNEAKTYYFINGGGSCFGGMKVAVNVENPQPAPSPSQLTGVKSIKNGSPSRFGGHVVTLMAVLANVLLAWAIL |
| *Populus trichocarpa* | PtSC2 | Potri.001G268700 | I | MANFRKTILVVSFLTTALCGVSMATVYQVGDSAGWTSMGQVDYQDWAASKNFHGGDTLVFNYNNQFHNVKQVTHQGFESCNATSPLATYTNGSDTVTLGKQLGHFYFICGYPGHCQAGQKIDILVAPATSNLSPAASPSSASSPYFSNLSWTLGVLGFCLLGFAY |
| *Populus trichocarpa* | PtENODL7 | Potri.001G273000 | II | MQQRRYSRLCLLSLSFSCLLLLLPSFLGPVDAYKNYTVGDSLGWYDATVESNVNYQKWADGKNFSLGDFLIFNTDSNHSVVQTYNFTTFKSCDYDNSEGNETVEWSSTNPSNTLTQAVTVAVPLVKEGPTFFFSGYYDGEQCQNGQHFKITVSHGKGLPDSLKDPSDQAPAPNAADYGSTPDTVVPFDFNNPHDQDTDVKKDSGSISLHVKNLDMKLNGILLSLGILYMF |
| *Populus trichocarpa* | PtUC3 | Potri.001G332200 | I | MEYRVCLVLVFVALITKEAMAAQHVVGGSQGWEESTDFSSWASGQKFKVGDQLVFKYTSGLHSVVELGGESAYKSCGLGTALNSMNTGNDVVKLNKPGTRYFACGTLGHCGQGMKVKITVESGTAPSTPESPSSSSSPAASSASAMHSYFATFVLLTALVATSLLYMF |
| *Populus trichocarpa* | PtENODL8 | Potri.001G338800 | II | MSLLLMTILSSFLYLSVSSFEYEIGANEGWVVPPANDTRIYNDWASENRFQVDDTIRFKYRKDSVMEVSVEDYKKCNSSHPNFFSNTGNTVYHLNHSGYFYFMSGVSGHCERGQRMIIKVISSDQETNSGGEKSSASPSAPVLSSGVFKALLSQLAMSYVASYVFY |
| *Populus trichocarpa* | PtENODL9 | Potri.001G419200 | I | MANISYQNKVFHVLGLLCFLLLIQKNNAFQYQVGGGSKGWTVPDNTSSSSKSYYNDWAERTRFRIGDSLLFAYDPSQDSVLQVSKGDYENCTTKNPIAAFSDPKTVFTFNHSGHHYFISGNKDNCLKNEKLVVVVLADRSSNHSANTNQTTAAPSPSLGYSDMVPAPTPSGVETPPAPAGIADINPTPAPAGVSPNSASSLFVSFIGSMGAFFASSLILSF |
| *Populus trichocarpa* | PtSC3 | Potri.002G052500 | I | MGSMKKTLAISCFMMALHGVSMASTVYQVGDSVGWTSMGGVDYQDWAADKNFHAGDTLVFNYNIQFHNVKQVTSQDFETCNATFPIATYTSGSDAINLERLGHVYFICGFRGHCLAGQKIDILISPVTSGPSPAHWPLSSRSSASSDLYFNKLYWTLSVLVLCLSQFAY |
| *Populus trichocarpa* | PtENODL10 | Potri.002G073800 | II | MVNVAALLCLMVLTGHVHAATYTVGGSGGWTLNMDSWPKGKRFKAGDTLVFTYDPTIHNVVAVNRGGYSSCITPAGAKVYKSGKDQIKLSKGQNFFICNVAGH |
| *Populus trichocarpa* | PtPLC1 | Potri.002G074000 | II | MVQGRGSADLVMVNVAALLCLMVLTGHVHAATYTVGGSGGWTLNMDSWPKGKRFKAGDTLVFTYDPTIHNVVAVNRGGYSSCITPAGAKVYKSGKDQIKLSKGQNFFICNVAGHCESGMKIAINAA |
| *Populus trichocarpa* | unknown | Potri.002G101200 | VII | MEYGLSHLHEISPFHILRALWNQVDLIILTPYLLIPLYNTPNDDPKSNPNTFFSSLLFSRKMAGLISRSVPCAILVVLCTVVPILAKDHTVGDSSGWAIGMDYSTWTSGKTFSVGDSLVFNYGGGHTVDEVRASDYSTCTTGNAITSDSSGATTIALKTAGTHYFICGVPGHCGSGMKVAVTVAAAGSSTSPSSGTPSSDGTTTSPAGSNVTNYKPSSNNVPDSSLGINISPFVALAGTFVAVFVVVFS |
| *Populus trichocarpa* | PtUC4 | Potri.002G101300 | I | MAGLISRSVPCAILVVLCTVVPILAKDHTVGDSSGWAIGMDYSTWTSGKTFSVGDSLVFNYGGGHTVDEVSASDYSTCTTGNAITSDSSGATTIALKTAGTHYFICGVPGHCGSGMKVAVTVAAAGSSTSPSSSGTPSSDSTTTSPAGSNVTNYKPSSNNVPDSSLGINISPFVAIAGTCVAVFVMVF |
| *Populus trichocarpa* | PtENODL11 | Potri.002G150600 | I | MRLATILHLAIIAVLITAATSQAPPIRYINHTVGDNAGWFFNSTTNTTAANYSSWAASQTFNLGDYLIFRTSSNQTVIQTYNLTTFKDCSIDHSSDNDTVVYNGGNTVFDQALTIPVPLTIQGPNYFFSDANDGIQCQYGLAFEINVNRGLGLPPSLNQPPPPPYREPPGPDSASPPITIPAGGKGLGNEGFKNGLSVHVIACAVLFALLAVNGGIGVVFS |
| *Populus trichocarpa* | unknown | Potri.002G156100 | VII | MATVYQVGDSAGWTSMGQVDYQDWAASKNFHGGDTLVFNYNNQFHNVKQVTHQGFESCNATSPLATYTNGSDTVTLGKQLGHFYFICGYPGHCQAGQKIDILVVPATSNLSPAASPSSASSLYFSNLSWTLGVLGFCLLGFAY |
| *Populus trichocarpa* | unknown | Potri.002G156400 | VII | MATVYQVGDSAGWTSMGQVDYQDWAASKNFHGGDTLVFNYDNQFHNVKQVTHQGFESCNATSPLATYTNGSDTVTLGKQLGHFYFICGYPGHCQAGQKIDILVVPATSNLSPAASPSSASSLYFSNLSWTLGVLGFCLLGFAY |
| *Populus trichocarpa* | PtSC4 | Potri.002G161300 | I | MANFRKTILVVSFLTTALCGVSMATVYQVGDSAGWTSMGQVDYQDWAANKNFHVGDTLVFNYNNQFHNVKQVTHQGFESCNATSPIATYTNGSDTVTLEKLGHFYFICGYPGHCQAGQKIDILVAPATSNLGPAPLSQISPSSASTLSFSNLSWASGVLLASCLLGFGY |
| *Populus trichocarpa* | PtENODL12 | Potri.002G241500 | IX | MARQGRCSAIGVVLASTLLVILSLQFKIAIAKAATFTVGDTSGWTFNIQSWTDGKKFKAGDSLIFNYDPSLHDVATVDVDGYDGCTLSPSSSTYTSGKDTIKLKEGQNYFICSLPSHCDWGLKIAVNASA |
| *Populus trichocarpa* | PtENODL13 | Potri.003G047300 | I | MAKIAVALFMMMALCGVCFGAGYNVGESDGWTIGVDYNQWASTKKFQVGDTLVFNYNTMFHNVLQVTKQDYESCNVKSPVATFASGRDFITLDKAGHSYFVCGFPGHCQAGLKVAISVRASSSQSPDVPSPPSTPREIPPPPPPQTLSAPGQPNFHPPPLGSPNVPLPPGFPNFGTPSGPGFPYLPPFESGASLHSSNLKAAMLSVIMTNLFAVFAY |
| *Populus trichocarpa* | PtENODL14 | Potri.003G050500 | I | MTSLVVFFCICFIITVASMNGLVIAERVFKVGDVFGWQEPGQNSSSLYAQWATRNRFQVGDSLSFDYKNDSVIEVNKWGYYHCDASKHIVAFNNGNRVFKLDKSGLFYYISGTPSHCKNGQRLLVEVMGLHHHSPPFIAAPPGYLAPSPQLSSGVSVSGTLGSLSMALMATLIALLWSLA |
| *Populus trichocarpa* | PtENODL15 | Potri.003G117900 | I | MASPNKMFMIIAIVAVSVPSILATEHLVGDATGWKPGFDYGAWANGKEFHVGDTLVFKYRAGAHNVLRVNGTGFQECKAADDTVPLSSGNDVISLSTPGKKWYICGFAEHCESGNQKLAITVLAQLGSPSTSPSPSPTGTSPSGATSGSTVSRYYGLIVAIVGMVMF |
| *Populus trichocarpa* | PtUC5 | Potri.003G150300 | I | MGKLVLVFSLVVLGLAVTCKAATYMVGDNSGWDISTDIDTWAQDKTFAVGDVLMFQYSSSHSVDEVKKEDFDSCNTTNVLRTFTTGNTTVSLTNPGTRYFVCGNKLHCLGGMKLQVNVASNQADSPTGAPQTHPGGNISQPSSKSNNPASVIPTSAGSVYGGRDSIVMAFLGFMATLSWAVQV |
| *Populus trichocarpa* | PtENODL16 | Potri.003G183300 | VIII | MPHSIIVQKSVLHHPHSRKKAAATITRDCTMVSSSAQLIFFCFFILFSATATTATDHIVGANKGWNPSINYTLWANNQTFYVGDLISFRYQKTQYNVFEVNQTGYDNCTTEGALGNWTSGKDFIPLNEAKRYYFICGNGQCFNGMKVTILVHPLPPPPSGSIAANSTPSGSAAPVVFHKGLVGLRAFVLAFASIWFGSGWI |
| *Populus trichocarpa* | PtENODL17 | Potri.004G121100 | I | MERSGFGSSMTVAVAVAVLVFAMMVMVPEVSATRWTVGSNMGWTTNVNYTMWAQDKHFYNGDWLYRNQMNVLEVNKTDFESCNSDHPLHNLTRGAGRDVVPLNVTRTYYFISGKGFCYGGMKLAVHVANPLPPPTAAPLNEKSGSSSSILKCQYVLSTVFAIGALWDAFVWFW |
| *Populus trichocarpa* | PtSC5 | Potri.004G169700 | I | MVFKKTLVIFFLTTALCGVSMAARLYQVGGSAGWTSMGDVDYHDWAANKKFHVGDTLVFHHNYRFHDVKQVTRQDFKSCNVASPIASYYNHHGYDSPTLNRLGHFYFISAFPDHCQAGQKIDILVTPETSSPTSPPLSSPISAASATSSAPSLHLSWTLSVLAFCLLGFAC |
| *Populus trichocarpa* | PtENODL18 | Potri.004G171100 | II | MVFKKTLVIFFLTTALCGVSMAARLYQVGGSAGWTSMGDVDYHDWAANKKFHVGDTLQVTRQDFKSCNVASPIASYYNHHGYDSLTLNRLGHFYFISAFPDHCQAGQKIDILVTPETSSPTPPPLSSPISAASATSSAPSLHLSWTLS |
| *Populus trichocarpa* | PtENODL19 | Potri.006G009000 | I | MAVVKKMLMLLVLVSVSLGVGAQVHHIVGGERGWDPYADLGLWSSARTFRVGDKIWFTHSAAQGKIAEVETKEEYLTCDVSNPIRMYTDDIDSISLDGEGIRYFTSSNSGKCKSGLKLHVEVVPEGKTDTTTATPQVVTSESSDKAVAAPPEISGSAHIGASLALLVAGFWLCYMGV |
| *Populus trichocarpa* | unknown | Potri.006G067200 | IX | VPHNKSYYENWVGGKKFEVGDSLEFIWSIVPHNVLEVTTKTEYDSCVKTNGTPEDTSPATFDLTKNGTYYYICTIGSHCDSGQKVTIVVGNGSSSSPPISMPNNHAALSAVLSTITIVAASLVHLCSFLLY |
| *Populus trichocarpa* | unknown | Potri.006G067300 | VII | MAWAFGKKNHKEWLPPISYATIQALYSKHSARSTTRKNRIHLNRKNRLSMARGLNMAFLAAIAIAALVQTSVAQTTHTVGDTTGWAIPTGDPAFYSSWAANQTFNVGEILVFNFMANAHDVAKVTKADYDACTTSSPISLVETSPARINLDASGEHYFICNFTGHCSAGQKMMINVSAASSSPSPAPQTSSPAPQPSTPTPQTSSPAPQPSTPTPQPSPSPQPSTPAPQPSTPAPQPSTPAPQPSTPTPASGPSPPAPTPASGSPPSPPTATPPTPAPASGSPPSPPTATPPSTVAPPNSARSLGFAGFTTFLSIFVVFLCY |
| *Populus trichocarpa* | PtSC6 | Potri.006G067400 | I | MRSLIVFVVLGAVSLLLRGSEAVDHEVGDTTGWKSPSSTSFYSDWASGKTFALGDTLKFTFTTGAHDVATVSKSDYDNCNTGSQNNLLTTGPATITLNVTGDMYFLCTIAGHCSAGQKLAITVAAGNTTSPGTSPPPPSAASSLVATFALMFVSIAISLMYCF |
| *Populus trichocarpa* | PtENODL20 | Potri.006G184100 | I | MASHKVALLSSILVVSLFVTFTEARDIMVGGKNYSWKIPSSESDSLNKWAEASRFRVGDTLVWTYDPKKDSVLQVIKKDYETCNTSSPLVTYKDGNTKVKLDKSGPYYFISGADGHCEQGQKLITVVMSMRSHFMGISPAPSPVEFGGPAVAPTSTGGVNLRGSLGLSFGVLTGLILL |
| *Populus trichocarpa* | unknown | Potri.006G259000 | VII | MAAVYQVGDSAGWTSVGQVDYQEWAASKNFHVGDTLVFNYNSQFHNVKQVTQQAFEACNATSPIATYTNGYDTVTLEKLGHFYFICGYPGHCQVGQQIDILVSSPTSSLSPSPSTDQTTEPSAASSLYFSYNVCWTLGVLLAFCLSGFAY |
| *Populus trichocarpa* | unknown | Potri.006G259100 | IX | MAAVYQVGDSAGWTSMGQVDYQEWAASKNFHVGDTLVFNYNNQFHNVKQATQQGFEACNATSPIATYTNGYDTVTLEKLGHFYFICGYPGHCQAGQKIDILVSSPTSRKVLELPAICRV |
| *Populus trichocarpa* | PtENODL21 | Potri.006G264600 | I | MACFQRAVACALVLMSLFVGLSQAKDLLVGGKTDAWKIPSSESDSLNKWAEKARFLVGDSLAWKYDGQKDSVLQVTKEAYASCNTTSPIEEYKDGNTKVKLDRSGPFYFISGAEGHCEKGQKFVVLVLSQKHRHTGISPAPSPAEFEGGPAVAPTSSAYTLRGGFLVAFGVLVLGLILM |
| *Populus trichocarpa* | PtENODL22 | Potri.007G104600 | VI | MNAIIATIATLLSLIFQQCYIARAKGPVTYIVGDDLGWTLDGYPESWTGGKKFYAGDILEFKYDTEDANVVVVEKKDHDDCSVSDSSVFYRSGDDKIQLQFGANYFICSWPLNQCQMGMKVAINATARPPSLLVH |
| *Populus trichocarpa* | PtUC6 | Potri.007G120200 | I | MVRTFTSLALMAMMLRLAMAANYTVGGPNGGWDATTNLQAWAASNQFLVGDNLIFQYGLVHDVNEVSKADYDSCQITSPLKSYSGGTTVIPLSSPGKRYFTCATPGHCAGGMKLEIDTLATSTPPPASPLTPPPASPLTPPPASPSLPSPPTTSTLPPASTDIPPASSPPPEIFNLSPSQSPEMTPTMSPSAPRTSPLTSPTPSPATAPSIDGFMKTPLASSASKESLQRSLTMGISLVIMMILLAI |
| *Populus trichocarpa* | PtSC7 | Potri.008G151000 | II | MALVKRALALLMSITLAMELIHAAVYKVGDSAGWTTIGNFDYKKWSATKTFQVHDIILFKYNAQFHNVMRVTHAMYKACNTSAPLATYTTGNDSITIKTRGHHFFFCGVPGHCQAGQKVDINVLQSNEMAPTSSVSSSESSPPVPSAKVPGPAPSNAMPLKALKSPSGNIGLAMAVLATFWINFA |
| *Populus trichocarpa* | PtENODL23 | Potri.009G067300 | II | MEQMTGLLFLSFSSLILLSFSGSVDAYKNYTVGDSLGWYDTTVKSNVNYQKWADGKNFSLGDFLIFNTDNNHSVVQTYNFTTYKLCDYDNSVDNVTVEWSSANPSNTLTQGVTVAVPLLKEGPTYFFSGDYDGEQCDNGQHFKLTVSHGKGLPDSLKDPSDQAPAPNAADYDSTPDTTVPFDLNNPHDQDTDVKKDSGSISLYGKFLDMKLHGILLLLGIVYLF |
| *Populus trichocarpa* | PtENODL24 | Potri.009G106000 | II | MARLTHAQVLILLLSASMWAISMASRQYGFNNTDWSYKRRPCRQNSTAAPNKIVVGGSQNWTFGINYADWALKNGPFYFNDTLVFKYDPPSDTNTHPHSVYLLPNLWSFLKCDLSRAKLVASETQGGGDGFEFVLKSWQPHYFACGGGAGFHCNNGTMKFFVMPMFRRRY |
| *Populus trichocarpa* | PtUC7 | Potri.009G136200 | II | MGEAKIFLMILIMVFLKGAVSEVHTVGDELGWNTGANFGSWSRKYNFSVGDTLVFKYVKGQHNVYEVIEATYRSCNGSTGVLATYESGNDQIELNKAKKYWFVCNFAGHCLGGMRFFIDVKEANSTNIRPTTPQSEPIPPPPPANSCAAIYVFDGWSFWVSLVAFGVLLQL |
| *Populus trichocarpa* | PtSC8 | Potri.010G089900 | II | MALVMRAVALLTVMTLMLELIHAAVYKVGDSAGWTASGNIDYKQWSATKTFQVGDVILFEYNAQFHNVMRVTHAMYKACNTSAPMATYTTGNDSITIKTRRHHFFFCGVPGHCQAGQKVDINVLRSDERAQTPASSSMSSPPVPSAKVAGPASSNALSLKALRSPFGSFGLAMAVLATFFYINLA |
| *Populus trichocarpa* | PtENODL25 | Potri.010G243600 | I | MALQRHPCIAILFAQAIIATFDATSGLRYTVGGSIWSIPPHPDFYCNWSSSHTFYIGDVLVFDFEYEFFNVIQVPKLDYESCTALNPIRILTRSPALAILIHEGVNYYICNISNYCDLGLRFSVVVHKFYYSTGHSPAPSPLPSLPPSSPPTLSPYPAPGPSQAGWTDVSQPSVPNNSPIAPNAGRRKGLRANSGVTVVGLACALCLGTLFVLL |
| *Populus trichocarpa* | PtENODL26 | Potri.011G117800 | I | MESQGSLCLLWALLACYLFSFSVAYNSFYVGGNDGWVINPSESYNHWAERNRFQVNDSLVFKYNKGSDSVLRVTKDDYNSCNTKKPLKTMDSGSSVFQFDKSGPFFFISGNEDNCRKGQKLIVAVLAVRTKQTPTPAYPPATSPKAPSPEGHNPAQAPSRSSAPIAKPPTSSHVPSVSPVSPSPIANAPSSNAPTGAPGPSPVTKTPQISPVPSKSPSPSPYAKPPAPAHSPESLTGSPGPSPVPLKSPSPLANTPSPSYHPVASPTPARSPSPSSPTPAKPPSSSTPSPTPESSSGPSLSPRSNEADLAPAPAPAASWAATPSTTMVIVASLLISSAISGWP |
| *Populus trichocarpa* | PtENODL27 | Potri.011G135400 | I | MAYTTCKDNVFHILGLLCFLLLIQKNNAYPFPVGGPKGWTVPDNTSSKSYFNDWAEHHRFQRGDSILFVYDASQDSVVQVTKEGYENCTAEKPLATFNDGHTVFKFNQSGPHYFISGNRDHCQKNEKLAVVVLADRSTNATASPPSPGSSDMVPAPTPSSEESPPAGTVDINPTPPPTGAPPNSASSMFVSFFGSMGAFFASSLILAI |
| *Populus trichocarpa* | PtENODL28 | Potri.013G030000 | I | MASCRIFMIIAIVAVFVPSILATEHMVGDKKGWTLGFNYQTWAQGKAFYVGDTLVFKYTPGAHNVLSVNGTGFEECKAADDIVPLTTGNDVITLSTPGKKWYICSVPGHCESGNQKLFITVLPQLSSPATSPFPGPTDTSPSGAAGNIASTYYGLIAAIVGIFGMIMF |
| *Populus trichocarpa* | PtENODL29 | Potri.013G030200 | I | MASCRIFMIIAIVAVFVPSILATEHMVGDKKGWTLGFNYQTWAQGKAFYVGDTLVFKYTPGAHNVLSVNGTGFEECKAADDIVPLTTGNDVITLSTPGKKWYICSVPGHCESGNQKLFITVLPQLSSPATSPFPGPTDTSPSGAAGNIASTYYGLIAAIVGIFGMIMF |
| *Populus trichocarpa* | PtENODL30 | Potri.013G054500 | II | MASSQFIAFALVTIILPTLTMAAEHIVGDDKGWTVNFNYTTWASGKVFHVGDTLVFKYQPPHNLYKVDGNGFKNCVASGEALTSGNDIITLGSTGKKWYICGFGKHCSELGQKLVINVEAEAPAPTPIPNAAYGLAASGYQIIVAAVAVVAGMIVA |
| *Populus trichocarpa* | PtSC9 | Potri.013G061300 | II | MASRCVLAIFVLIAAIVPMTTLATEYIVGDESGWTLGFDYHAWAAGKNFLVGDELVFKYPVGAHNVFKVNGTEFQNCIIPPADRALTSGDDTIVLASPGKKWYICGVGKHCEFGQKLAITVQSLAPTPSPAPSPLYAKPDEAVKGKRPFFTLRWW |
| *Populus trichocarpa* | PtUC8 | Potri.014G049600 | I | MANIASALLILVLAAPAAYAATTYTVGDSSGWSTTFGDYTTWVSGKTFTVGDSLLFKYSSTHTVAEVSKGDYDSCSTSNLGKTYTDGSSTVPLSTAGPMYFICPTSGHCSGGMKLAITVVAASGTPSTPTTPPVDDGSTTPPTTSGSPPTTPSTTVAPPPPSKSNNGATSILYNMMLGVFLVFGTTVALMGQ |
| *Populus trichocarpa* | PtENODL31 | Potri.014G072700 | II | MTGATILHLITMVVLITAATSQAPPTKYINHTVGDNAGWFFNSTTNTTATNYSSWAASQTFNLGDYLIFKTSSNQTVIQTYNLTTFKNCSIDDTSYTDTFVYNGGNTVFNQAFTIIVPLTIQGPNYFFSDASDGIQCQHGLAFDINVSRGLGLPPSPNQPPPPPYREPPGPDSAYPPIIFRPRERGQEIVDLKTGPACK |
| *Populus trichocarpa* | PtENODL32 | Potri.015G052000 | VII | MASDLFNGSCKVFLSLIIFSSIFQFCFVISTEFLVGGQDGWTIPKKDSQMYIDWASKNRFKVDDTVQFKYNKDSVLVVTEEEYQKCRSAHPLFFSNNGDSVFKLDRPGLFYFISGVAGHCERGQKMIIKVLELETPPQSANDTSPPDHTNKKNGAVQMPPAIIPPIIVLPSLFFLGFLFV |
| *Populus trichocarpa* | PtENODL33 | Potri.015G113300 | VI | MSTPKQKSLFLAVIFTSLRYLSVYSFEYQIGGNENWVVPPAIDTRIYVDWALGNRFQVGDTFSFNFLGDSVMKVRVEDCKKCHSRHPNFFSNTVYHLNYPASSYFISGVSGHCEKGQRMIIIKVISTDQETNSSLQLFLLQEC |
| *Populus trichocarpa* | PtENODL34 | Potri.015G114300 | VI | LFLAVIFTSLRYLSVYSFEYQIGGNENWVVPPAIDTRIYVDWALGNRFQVGDTAREKDSVMKVRVEDYMKCHSRHPNFFSSTVYHLNYPASSYFISGVSGHCEKGQRMIIIKAVEDRFLAIDLAGNGHGVV |
| *Populus trichocarpa* | PtENODL35 | Potri.015G114600 | IX | MAVIFTSLRYLSVYSFEYQIGGNENLVVPPAIDTRIYVDWALENRFQVGDTARDQFKHKEIRDLPLPSISWVFIIFFCEGFKHRKDSVMKVRAEDYKKRNSRHPNFFSNTVHHLNHPASSYFISGVSGHCEKGQRMIIIKVISTDQETNSNLQLFLLQEC |
| *Populus trichocarpa* | PtENODL36 | Potri.015G114700 | VI | LFLAVIFTSLRYLSVYSFEYQIGGNENWVVPPAIDTRIYVDWALGNRFQVGDTAREKDSVMKVGVEDCKKCHSRHPNFFSNTVYHLNYPASSYFISGVSGHCEKGQRMIIIKLFLLQEC |
| *Populus trichocarpa* | PtENODL37 | Potri.015G115600 | IX | MAVIFTSLRYLSVYSFEYQIGGNENWVVPPAIDTRIYVDWALENRFQVGDTARDQFKHKEIRDLPLPSISWVFIIFFCEGFKHRKDSVMKVRVEDYKKCNSRHPNFFSNTVHHLNHPASSYFISGVSGHCEKGQRMIIIKVISTDQETNSNLQLFLLQEC |
| *Populus trichocarpa* | PtENODL38 | Potri.015G117100 | IX | MAVIFTSLRYLSVYSFEYQIGGNENLVVPPAIDTRIYVDWALENRFQVGDTARDQFKHKEIRDLPLPSISWVFIIFFCEGFKHRKDSVMKVRAEDYKKRNSRHPNFFSNTVHHLNHPASSYFISGVSGHCEKGQRMIIIKVISTDQETNSNLQLFLLQEC |
| *Populus trichocarpa* | PtENODL39 | Potri.016G015200 | VII | MSRASLIKEKKVCCAVKMAMTKKILMVLVLVLVLVSLNDGAQVHHVVGGDRGWHPYSDIGSWSSARTFRVGDKIWFTHSAAQGRIAEVETKEEYLTCDVSNPIRMYTDDSDGITLDGEGVRYFTSSSSDKCKNGLKLHVEVVVPEARTDTTTAQVASEGSDKAIAAPPESSAPSHFGASFALLMAGFWLSYMGI |
| *Populus trichocarpa* | PtENODL40 | Potri.016G050700 | II | MALQKTIAISFLMMSLCGVSMAPVYQAGDSAGWTRMGQVDSKDWAANKSFHVGDTVVFNYNSQFHNVKQVTQQGFEPCNATFLIATYTSGSDGVTFSIKFVHVTLSLIDLNGEASLFPCSQVVIRSNT |
| *Populus trichocarpa* | PtENODL41 | Potri.017G011200 | I | MGSKRFSGSLFVMLVLGFLLGVSRGYKFYVGGKDGWATNPSERYSHWAERNRFQVNDTLFFKYKKGSDSVLIVSKDDYNSCNTKNPIKSLTDGDSTFIFDRSGPFFFISGNADDCNKGKKLIIVVMAVRPKPLPPTPYSPITPASSPQPTSSPPAVSPDARSPSDSAGPAQAPSTNSKSGSSGLTAGSLSVGLVLGASIGVSFILGGFLRVV |
| *Populus trichocarpa* | PtENODL42 | Potri.017G012300 | I | MGSKRFSGSLFVMLVLGFLLGVSRGYKFYVGGRDGWATNPSERYSHWAERNRFQVNDTLFFKYKKGSDSVLIVSKDDYYSCNTKNPIKSLTDGDSSFIFDRSGPFFFISGNADDCNKGKKLIIVVMAVRPKPLPPTPYSPITPASSPQPTSSPPAVSPDARSPSDSAGPAQAPSTNSKSGSSGLTAGSLSVGLVLGASIGVSFILGGFLRVV |
| *Populus trichocarpa* | PtENODL43 | Potri.017G088500 | VII | MENSGRTTIGKTVLSMAITAVTVMMIVECAAAEQLYKVGSRGWIPNYNYTDWLNQSHEHFYVGDWLLFVFDKHSYNVLEVNETSYENCNDQGFIKNITRGGRDVVQLTEARRYYFLSSGGYCWNGMKVAINVEDFAPTPAPASSTENGSPSNIVSRQMIILIAFCVALEWMVLFL |
| *Populus trichocarpa* | PtENODL44 | Potri.017G088600 | I | MERGSGFGSPMMVALAVLVFAMVVMVPEVSATRWTVGSNMGWTSNVNYTIWAQGKHFYNGDWLFFVYDRNQMNILEVNKTDYESCNSDHPLHNWTRGAGRDVVPLNVTRNYYFISGKGFCYGGMKLAVHVENPPPPPTASPLDEKSGSPSSIFRSQYVLPTVFAIGALWDAFVRFW |
| *Populus trichocarpa* | PtENODL45 | Potri.018G018200 | I | MASFQRAAVFSLVLMSLLWGSSQAKDLLVGGKTDAWKIPSSESDSLNKWAGKARFLIGDSLVWKYDGQKDSVLQVTKEAYAACNTTNPIEEYKDGNTKVKLDKSGPFYFISGAEGHCEKGQKIVVVVLSQKHKQVGYVGSPAPSPVEFVGPAVARTSSASNLKGGLLVALGVLVLGLF |
| *Populus trichocarpa* | PtSC10 | Potri.018G128800 | I | MGSRLCFNIGFLIVASVGLLHGAYAANTYTVGGDLGWIIPPNSSYYEEWTSQSTFQIGDSFVFNWTTGTHTATEVSTKEEYDNCTKMGLILKDAGVKVTFKDNDTHYFLCSEGTHCEQGQKMIIKIGDGIPPSFAAPSLTAAAALSALFFSTLAIFFLN |
| *Populus trichocarpa* | PtSC11 | Potri.018G128900 | I | MASRLCFNIGFLIVASAGLLHGAYAANTYTVGGDLGWIVPPNSSYYEEWTSQSTFQIGDSFVFNWTTGTHTATEVSTKEEYDNCTKMGIILKDAGVKVTFNANGTHYFLCSEGTHCEQGQKMIIKIGDGIPPSFAAPSLTAAAALSALFFSTLAIFFLN |
| *Populus trichocarpa* | unknown | Potri.018G129000 | VII | MSLLKTFLKDYIYIPSRYFRNQRILKRDCAPTTLIVRGKKMASRLCFNIGFLIVASVGLLHGAYAANTYTVGGDLGWIVPPNNTYYEEWTSQRTFQIGDTFVFNWTTGTHTATEVSTKEEYDNCTKMGMILAFAGVKVTFNENGTHYFLCSEGTHCEQGQKMIIKIGDGIPPSFAAPSLTAAAALSALFFSTLAIFFLN |
| *Populus trichocarpa* | PtSC12 | Potri.018G129200 | I | MARGLDMAFLAAIAVAALIHGSAAQSTHTVGDTTGWAIPPTGSAFYSTWAASQNFSVDDILVFNFAANTHDVAKVTKADYDACTTTSPISLFATPQVRITINASGEHYFLCNFTGHCSGGQKLMINVSAASSSPSPSPAPQTSSPTPQPSTPAPQPSTPAPQPSTPTPQSSPAPQPSTPTPASSPTPASSPSPPTPASSPSPPPTTPPSSSPPSPPTTTPPTSPPPPNSATSLGLAGFTTFLSIFVALCY |
| *Populus trichocarpa* | PtSC13 | Potri.018G129400 | I | MGSTLVAFVVLGAASLLLHSSKAAVYEVGDSTGWQAPSDTSFYSNWASGKTFTVGDTLTFTFSTTVHDVATVSKSDYDNCNTASQSNVLTVGPATITLNATGNQYYFCTLSNHCTRGQKLAITVAASSTPSPPGTPPTTPSSSPPPPSSSTPSPPPPPSASSSLVATFALVFMSIAISFMYYF |
| *Populus trichocarpa* | PtENODL46 | Potri.019G037800 | I | MASYQLIALALVTIFLPTLTMAAEHIVGDEQGWTVNFNYTTWASGKVFHVGDTLVFNYKPPHNLFKVDGAGFKDCAASGEPMASGNDIITLSSPGKKWYICGYGKHCSELGQKLVINVEAETPAPTPEPNAAYGLAASCYQIFAAAVAVVAMIAA |
| *Populus trichocarpa* | PtENODL47 | Potri.T070900 | I | MASYQLIALALVTIFLPTLTMAAEHIVGDEQGWTVNFNYTTWASGKVFHVGDTLVFNYKPPHNLFKVDGAGFKDCAASGEPMASGNDIITLSSPGKKWYICGYGKHCSELGQKLVINVEAETPAPTPEPNAAYGLAASCYQIFAAAVAVVAMIAA |
| *Vitis vinifera* | VvSC1 | GSVIVT01001147001 | I | MALAKILAALLMVMALCEVSIAATVYHVGDSTGWTIGKVNYTLWSQTKDFVVGDTIIFEYSNQYHNVLQVTHDNFKSCNATAPIATFATGNDSITISKYGHFYYLCGIPGHCEAGQKVDIRVPHPPESPSLSPSPSPITSPSPSPITSPSPSPSPSPSPITSPSPSPSPKPWPSSPSPSPSAWPTPSSPPPSWGNSPAPAPYWGIAPAPAPSPASSLHVSMGLLGPSMAVLAFVVTAFAY |
| *Vitis vinifera* | VvUC1 | GSVIVT01001807001 | I | MEGLYRVLSALVVVGLLTNKALATQHVVGGSQGWDESSDYSKWASGQTFEVGDQLVFKYTPGLHSVVELPNESAYKNCDVGSALNSMNSGNNVVKLSKAGTRYFACGTIGHCDQGMKLKVSTVSADASSSPGSTPPSSSSSSSSSPTSTSAASASFPFASMLPMASLLAISMLSMF |
| *Vitis vinifera* | VvENODL1 | GSVIVT01001982001 | I | MADSILTSDHLKKALHVLGLLSLLLLMQKVGATEFKVGGPNGWSVPADAALSYNQWAERNRFQRGDSLLFVYPAGNDSVLYVNKDDHNNCNTATPLELHKDGHTTFKLNQSGAHYFISGVVDNCLKNEKLVVVVLAERSKESLTPASPPSGSTDIVPPSGSTDIVPSPAPAGEESPSPPEGLVQIAPSPPPGGEEPSPSGASSVFISFIGSIGALVGSSLLLA |
| *Vitis vinifera* | VvENODL2 | GSVIVT01003354001 | II | MAEVKLVVALLLLVYVSWAGALVHHVVGGDRGWDTSSDVQAWLSNKVFRVGDKIWFIYSGGQEGVVELKSREEFDSCDVSNPIRTYTEGLDAVLMGSEGIRYFTSSKPKSCKDGLRLLVEVQSNLGWEPRRITSSAATVAGPSPPRSETGCPTKCSESETKSGSYTPQHRRALLSSGARRNSSRAM |
| *Vitis vinifera* | VvENODL3 | GSVIVT01003978001 | I | MASSPLMGSTTVLVLLLTIFSSLQRSTVSSFEFQAGEVKGWVVPHANDSKLYNDWASENRFKVGDSIRFRYKKDSVMVVSEADYKKCNSTHPIFFSNTGNTVYHLDHSGSYYFISGVAEHCQRGQRMIVKVMASEDPSSRGGGTPPSSAPTLSLGPSKLVFFQFLLSSVAAYLF |
| *Vitis vinifera* | VvENODL4 | GSVIVT01008220001 | I | MASISKASLLIFLFFSLHFFPVISVEFLVGDDDGWALPSSKSGEQMYNEWASHNRFKVGDTVHFKYEKDSVMVVTEAEYNKCHSAHPILFSNNGDTIFSLDRPGLFYFISGVAGHCERGQKMIIKVLEPPSPPSVPKQNGTSNSSNSSHSSGAVDMAAAISASTVGLFIISFFGVLLF |
| *Vitis vinifera* | VvPLC1 | GSVIVT01009090001 | IX | MIKVQIILNCYNPHSLTHKMAGVGVVCVVFLVLCAVMPSLATDYTVGDSTGWTMGADYSTWTSGKTFVVGDTLVFNYGGGHTVDEVSASDYSTCTVGNAITSDSTGATTISLKKTGTHYFICGVIGHCGSGMKLAVTVESGKTTAPPTSSSATPSSGAATTSPATPTATTTTTPSSTVCSLSILYLSFSLSYMGEMLVLRFFYCDE |
| *Vitis vinifera* | VvENODL5 | GSVIVT01009568001 | II | MKMGISFVAPGLILLLTACSLAISETDTIVVGGSENWRYGFNYTGWSLQHGPFYINDKLVFKYNPPSKNNSRHSVYLLPNLWSFATCDFSQAKLLANPQQGGGKGFVFELTNWRPHYFASGEEDGSQCEDGQMKFFAVPLPRWGK |
| *Vitis vinifera* | VvENODL6 | GSVIVT01011475001 | I | MSRFKCSRNPGGMLRMWWWWWLMAAAAAMVGGCEANLIKVGGKQGWGPNVNYTEWAKNKHFYVGDWLYFIFDKHYFTVFEVNETNYERCSEQEFITNITKGGRDVFNLTHPRPYYFLSSGGYCWHGMKLAINVTHMPAPAPSPSKSNAPPSASSPTPIILSIALLCPLLFKFFFHA |
| *Vitis vinifera* | VvENODL7 | GSVIVT01011476001 | VII | MCAVVLMLPDVSATRWTVGGNQGWSTNVNYTVWAKDKHFYNGDWLFFVYDRNQMNVLEVNETNYESCNSDHPLHNWTTGAGRDVVPLNVTRKYYFLSGKGFCYSGMKIAINVENPPPPPSASPIKESNDSPSSNYRGQIVVPAVFAIAAVWDSFLRLYW |
| *Vitis vinifera* | VvENODL8 | GSVIVT01013874001 | I | MGKGVVLGCVLVVLGFALTCSAATFYTVGDSSGWDISTDLDTWAKDKKFIVGDVLLFQYSSSNSVNEVTEESFKGCNMTDTLQTSSNGNTSIPLNRPGERYFVCGNKLHCLGGMKLQVNVQKDPAASPAGAPEASEGSLPRPSSKNNNPAAAIPDSTGFINGGMVSLLSAFLGSMATLLWIL |
| *Vitis vinifera* | VvENODL9 | GSVIVT01014311001 | VI | MRWWLVFAGFLCCCEGFKFIVGGKGGWVENPSEEYNQWAGRNRFQVNDTLFFKYQKGVGSVLVVEKDDYFSCNTENHNPTFTFDVYSRHPTFIFDLYSGRRHFTGTDTVIGNCYCTVNGYLVIRGYYNTDYCFRPLVLTFISLISFFINAFYFILFYFSIILILSWDDGEMFD |
| *Vitis vinifera* | VvENODL10 | GSVIVT01014693001 | II | MDSRKLLVFLLFTFFNFSDPPLVVSAQNVDFDFSSFTLRNFTLLGDSYLRNGVIGLTRNLGVPSSSSGTVICNAPIAFFDSESNTTASFSTRFSFSITNVNPSSYGDGLAFFLSPENETLGSPGGYLGLVNSSQLTKNRFVAVEFDTRLDPHFNDPNDNHVGLDIDSLNSMKTADPVLDENIDLKSGKSITAWIEYKNDQMKLKVFLSSSRSKPERPVLIVDIDLSEYLKELKYVGFSASTEGSTELHLIENWSFKTFGLVPAPPRLHFPPHNVSDNTVMIPPPSAVSDSGNKGHKRLGLGFGIAGPVFFCIVLSVFGYVSMKKWRGMRWEKSFKADILAGPREFSYKELKGATKGFHSSRIIGNGAFGTVYKAFFITTGTISAVKRSKHSHEGKSEFLAELSIIACLRHKNLVQLQGWCVEKGDLLLVYEFMPNGSLDKMLYQESEEGTLLKWSHRYNIIVGMASVLTYLHQECEQQVIHRDIKTSNIMLDGNFNARLGDFGLARLMDHDKSPVSTLTAGTMGYLAPEYLQYGKATEKTDVFSYGVVILEVACGRRPIEKDTDSQKMMNLVDWVWGLYSQGNILEAADKRLNREFKEEEMRKLLLVGLSCANPDCNVRPSMRRVLQILNDEAEPLLVPRMKPSLTFSLSLPLSIDDILHCFSAEELVMAFLLATKICNSAFFTSLLVSTAVVSVSSYTFQVGGEGGWTKPTGNETETYNGWAEKNRFHVGDSVYFKYQQDSVLVVNYTDYTNCNTSNPISKFEDGNTLFRFDGHGVFYFISGQPDHCQSGQKLIIRVMAQSEVKPPEPAPSPKTDGSAFSPEAAYVSALPPKAGGGNDRSSFNWVPPPSLNSTTKLSIASYFVTALGGVWVILYLFI |
| *Vitis vinifera* | VvSC2 | GSVIVT01015559001 | I | MAARVEIIGCLIVVAVLLQGAAAADTHHVGGNISWSIPTEGESAYTTWASGEDFKLGDTIVFNWTGTHTVARVSKDVYDNCTTANVLDNDIQATSPVNYTLNSTEPQYFICTIGRHCSLGQKVTISISSATSLTVGAVTTMLLVMAISFLTSI |
| *Vitis vinifera* | VvENODL11 | GSVIVT01015560001 | III | MARRFSTVVFAVMVVAALVQSSKAETHEVGDDLKWTVPSNGSVAYQNWAAGETFLVGDVLEFEFTTGFIVGDSLGWTVPSGGAVTYQNWAANKTFVVGDSLKFNFTTGAHDVAEVTKAAFTACNGTNPISHETEGGPSQSPSGSTTPPSPGSAPSFSVAGLSATLLSVAAALLY |
| *Vitis vinifera* | VvENODL12 | GSVIVT01015561001 | IV | MTTKIRAALVVVALAALLHTAVVVAQTTHVVGDSLGWLVPPGGPIAYATWADTQTFVVGDILVFNFTTGEQDVARVSKEGFDSCNSTNPISLKTTGPANFTLDTVGDYYFIGTMDRHCPLGQKLAIKVIDSSAGPSPPPSPRSPVTYTVGDILGWVVPPLGEVAYSTWAYNKIFIVGDSLVFNFINGTQDVAVVTKEAYDSCNTSSTITVYATSPTTITLTTTGMHYFSSTYELHCGLGQKLAINVIAKSTTPSPSGAATPPSSSVGASPSAGGPTAPPPSSSAPSRIFCIYGDIILIQKG |
| *Vitis vinifera* | VvSC3 | GSVIVT01015562001 | II | MAGRVNMVLVVLVAASVLHRTAAETYEVGNELSWRVPPNTTAYSTWASAYTFRVGDTLVFNFTTGSHDVAKVTKEAFNACNSSSPLTTLYTGPANYTLNSTGENYFFCTVGSHCSQGQKLSISVSASSSESPASPPGTTSTPSPPPPPSSAPSLAAATFSVIFMSIATDQYVEQCSCIILLHFYSPVAIFKHNHNKDHFTKGLFRFNLNHPSPPSQGIKIILKQR |
| *Vitis vinifera* | VvENODL13 | GSVIVT01015979001 | I | MSLILLLLISAAATISSVTATDHIVGANRGWNPGINYTLWANNHTFYVNDLISFRYQKNQYNVFEVNQTGYDNCTTDSATGNWSSGKDFILLDKAKRYYFICGNGGCFSGMKVSVLVHPLPTPPSASTAAAEISKPNSAAARAPRSGSMAFVGLVLWIGWIWLGSGWI |
| *Vitis vinifera* | VvENODL14 | GSVIVT01017030001 | I | MKNTNMVSSLGVLFTALIVLVAMNSSAGAATEFRVGDADGWRKPGVNETAMYEQWAKRNRFQVGDSLSFEYKNDSVLVVDKWDFYHCNSSSPISSFKNGKSVIKLERPGSFYFISGDPEHCKSGQRLVISVMALHPISQSPPAIALPPGNYFPISPSPSPLSSSGVLVSATLVPLLMAFIANLVGLV |
| *Vitis vinifera* | VvSC4 | GSVIVT01017332001 | V | MARLISMAVIVAVLAAMLHYSAAQTVHVVGDNTGWTVPQGGAATYTSWASGKQFVVGDTLVFNFATNVHDVAELSKESFDACDFSSTIGSIITTGPANITLATAGNHYYVCTIGSHCTSGQKLAISVSATPGASPPSSSTATPPPTTQGGDSSSSTVFAIVVLAAMLHYSAAQTVHVVGDNTGWTVPQGGAATYTSWASGRQFVVGDTLVFNFATNVHDVAELSKESFDACDFSSTIGNIITTGPANITLATAGNHYYVCTIGSHCTSGQKLAISVSATPGASPPSSSTAPPPPTTTNTPPSTPSPTAEVCPPTTAPSPKMNTPSPTTATPPSSQGGDSSSSTVFASVFVSLVSLTQMMARFMCMVGVMIVVAATLTVNFAAAQTVHVVGDSLGWTVPPNGAAAYTSWASNKQFMVGDILVFNFATNEHDVVELSKESFDACNYSNPIGSIITTGPANITLNATGNHYYICTIGRHCTSGQKLAITVSANPGSNPPSASPASPPPTTIATPSPTATPDDRTPTPSPSLMTDAPHAATPTQTPAAPPPTATIPPPGAPSPTATPDDCAPTPSPSLMTDAPPDATPTQTPAAPSPTATKLPPRTPSPTATPDDCAPTPSPTPATHL |
| *Vitis vinifera* | VvSC5 | GSVIVT01017936001 | I | MGMVGRRFSLLLLVVVATLQLSFAAVYKVGDSAGWTTIGNVDYKKWASTKTFHVGDIILFQYNAQFHNVMHVTHAAYQACNATNPLATFTTGNDSYTVSTHGHHYFLCGVQGHCQAGQKVDINVAGESSLLAPTPQATPSPVSSATSSTPPTAIPSPSPSDSPPSNALMGLLCKLGFAMSVFVVFVSTYA |
| *Vitis vinifera* | VvUC2 | GSVIVT01018292001 | I | MAMVAALLLLLLAAPVIYAENHTVGGSSGWDTGVDYSTWASGETFTVGDYLVFTYGSTHSVDEVSKSSYDSCATSNPTKSYTGGSNTIALTTAGSLYFLCPTTGHCSQGMKLAITVEANSSSTPPATSSPPSNSSSPSPSPSPTTSPSSPSPSGATSKFCSMSHLMVGLVLGFGMMFALVV |
| *Vitis vinifera* | VvSC6 | GSVIVT01019486001 | I | MHLVSFLMLAAVACFMTAPAAAFSHIVGGSFGWSTPGNLSFYEDWAKPRTFGVGDKLVFPFRTGVHSVVQVSEEEFKNCTQNDAIDMFYSGPTIIELPKTGTFYYYCGVGTHCEAGQKVKVTVVNAEGSAGTPITPNASVPAPADHKSSAKEGCDVGMVSGMLVLLLWVFI |
| *Vitis vinifera* | VvENODL15 | GSVIVT01019636001 | I | MLVQTRVSCYQYKVGDLDAWGIPSSANAHVYTNWSKNHIFKIGDSLLFLYPPSQDSVIQVTGQSFNACNLTDPILYMNNGNSLFNITSLGEFYFTSAVPGHCEKKQKLQISVLSGNGSSAFSPSYGPSALPDSSYPTVFGSIPAANSSSPSQRAPVFLAAVMGFVSWDLIRGRM |
| *Vitis vinifera* | VvENODL16 | GSVIVT01020567001 | I | MEFLKTSLLLLAIFMAFLCSSQGYVFYVGGKQGWSANPSEDYVQWAERNRFQVNDTLVFKYEKGQNSVLVVNREDYYKCNVENPINKYTDGNTEFKLDRSGSFFFIGGNADYCQKGQRLIVVVLAVRNETQTPTPTPSVPGNPPVLSPPSESPEGSPSPASSPAGDENSPAPAPHGSAPGLTRPVVWVLGVGFGVSVVLGNFVGLYI |
| *Vitis vinifera* | VvPLC2 | GSVIVT01023000001 | IX | MIHWPPLCILPLSLPPIFPLVLTKSSFPYMAMARGIAAAIPTATALLLWVVLHLRTAHAATYTVGGSSGWTFNVESWTDGKSFRAGDVLVFNYDPKDHDVVAVDQYSYDTCTVGEGAKVYESGNDSIELVKGENCFICSFLSHCDSGMKIHMIAL |
| *Vitis vinifera* | VvPLC3 | GSVIVT01023001001 | VI | MQWYLSMVRFVMGEGRGSAIVATVLLFCLLLHCDTAHATTYAVGGAKGWTLDVVGWPYGKRFMAGDILVFNYNAAAHDVVSVNKVGYNTCTMPRGASKVYHTGKDQIKLVKGQNFFICSFPGHCQSGMKIAITAM |
| *Vitis vinifera* | VvUC3 | GSVIVT01023002001 | II | MAVGRGSAVVAIVLVLCLVLPCDMVDAATFTVGGASGWAFNAVGWPKGKRFKAGDVLVFNYSPSAHNVVAVNKAGYNGCTTPRGSKVYQTGKDQIKLVKGANYFLCNFPGHCQSGMKIAVTAT |
| *Vitis vinifera* | VvPLC4 | GSVIVT01024007001 | IX | MEETQVLKCCSSFTIILIMISLGFFHGTNSETYTVGDEEEWDTGINYLTWSERYNFSMGDVLVFKYVAVQHNAYEVTEATYKSCDASTGVLAKYESGDDQVPLTEEKQYWFICTIAGHCLGGMRFTIDVKAASTNTTKGGSPPEMEPSPPPTNPDKDFASRPQGMGSYCLVAFGVVVILQIVS |
| *Vitis vinifera* | VvENODL17 | GSVIVT01027562001 | I | MALLIAIAASATPAASYTNHTVGGDAGWFFNTTTNTSIIDYSKWAANQTFSLGDFLIFKTNTNQTVVQTYNETTYQSCNTDDASDDDTFHYDAGSNEFGEAWTIAVALTIEGPNYYLSDADDGVQCQNGLSFAIDVKHGQGLPPSLNQPPPPPYMEPPSADSPPSPTVAVAGGQPSGNGDSRNAANMRAVLGVLLLLLV |
| *Vitis vinifera* | VvENODL18 | GSVIVT01028654001 | II | MALQGRCSANHAIALTTILVFVLLHVKASQATTFMVGDSSGWTFNINNWASGKKFKAGDKLVFKYNPSFHNVVAIDEDGYNGCSTASPSSKIYSTGNDAVKLLKGHNYFICGVPGHCDMGLKIRVNAS |
| *Vitis vinifera* | VvENODL19 | GSVIVT01029365001 | II | MEGPRPVWAVKVIMVIVIASIFFRCVSARNHTVGGPNGWDLASNLQVWSRSSTFYTGDNLVFSYTPNHDVLEVNQLDFARCRTINPLATHRDGETVVPLTNAGTRFFICGRRGHCTRGLRLMVQVLDLPSAAPAFPPAEESAASEPTRRERAPPPGKGDKSSPPPPAAMGAAPAKSPGAEAHCSVGVRIGGWVWCCLALAAVLKKKSMKR |
| *Vitis vinifera* | VvPLC5 | GSVIVT01030639001 | VII | MAANYTVGGPNGGWDTSSNLQTWASAQTFIVGDNLIFQFTPNHDVLEVSKADYDSCSTSNPTQTYSSSPAVIPLSSPGKRCFICGMAGHCSQGMKIELDTLASSSPPSASPSSPPTSSPASPVTPPTSSPASPVTAPTSSPPLETPPPPKSTPTISPSSPSTTPTSSPAPSSDVPSAEPPTSSPRPSPSSAHKGSFEGSLIMGLGLMMTILLAL |
| *Vitis vinifera* | VvUC4 | GSVIVT01031793001 | II | MGFTSARGVLTLLVLTASMLAVGLASRSIAGRPANYWNFGFNHSFWGPRNGHPFHPNNNNTTRPPKKFIVGGSEHWRYGFNYTDWALNNGPFYVNDTLVFKYDPPSKTTFPHSVYLLPNPWSFLTCDLSKAEQVATVAQGGGEGFEFVLKNWWPHYFACGEHDGLHCKEGMMKFFVMPLLGPYHG |
| *Vitis vinifera* | VvPLC6 | GSVIVT01031794001 | IX | MKFNKIEKCKPIKINVILGLMGFTCAQGVLTLLVLTASMLAVGTASRPIAGRPANYSNFGFNHSFWGPRNGHPFHPNNNSTRPPKKFIVGGSDHWRYGFNYTDWVFNNGPFYVNDTLVFKYDPPSKTTFPHSVYLLPNLRSFMTCDLSRAEQVATVAQGGGEGFEFVLKNWWPHYFACGEHDGVHCKEGMMKFFVMPLIGPYHG |
| *Vitis vinifera* | VvUC5 | GSVIVT01031795001 | II | MGFTSGQGIVTLLVLIASLLAVGMANRPIAGKPANHWNSGFNHSIWGPRNNPFHPNNTRPPKKFIVGGSERWRYGFNYTDWALKNGPFYINDTLVFKYDPPNSTTFPHSVYLLPNFGSFLTCDLSRAKQVATVAQGGSKGFEFVLKNLWPHYFACGEHNGLHCKEGMMKFSVMPLFRPCHG |
| *Vitis vinifera* | VvUC6 | GSVIVT01031796001 | II | MGFTCAQGVLTLLVLTASMLAVGTASRPIAVRPANHWNFGFNNSFWGPRNGHPFHPNNNNTRPPKKFIVGGSDHWRYGFNYTDWALNNGPFYVNDTLVFKYDPPSKTTFPHSVYLLPNLRSFLTCDLSRAEQVATVAQGGGEGFEFVLKNWWPHYFACGEHDGLHCKEGMMKFFVMPLLDNNGFQVYSIDACSK |
| *Vitis vinifera* | VvUC7 | GSVIVT01031797001 | II | MGFTSGQGVVTLLVLTASLLAVGMANRPIAGKPANHWNSGFNHSIWGPRNNPFHPNNTRPPKKFIVGGSERWRYGFNYTDWALKNGPFYINDTLVFKYDPPNSTTFPHSVYLLPNFGSFLTCDLSRAKQVATVAQGGSKGFEFVLKNLWPHYFACGEHNGLHCKEGMMKFSVMPLFRPCHG |
| *Vitis vinifera* | VvENODL20 | GSVIVT01035972001 | I | MAGFLRALCYSQVLVFLLSITCSAAKEFLVGGKTNAWKIPSSQSDSLNKWAESSRFLVGDSLVWTYDKEKDSVLKVRREAYISCNTSDAIEEYNGGNTKVTLDKSGPHYFISGADGHCEKGQKVIVVVLSQRHRLVGVSPAPSPSEVEGPAVAPTSDASSFKAGYLVALGVLVGFMWM |
| *Vitis vinifera* | VvENODL21 | GSVIVT01037359001 | II | MLRSGSAFLLLLLVFVSFSGYVEAYKNYTVGDSLGWYDNLHEPSVNYQKWVAGKNFSLGDFLIFNTDNNHSVVQTYNFTTYKHCDYENALENDTYQWSATDPSATNPVAVTVPVPLMKEGMTYFFSSDYDGWQCKHGQHFKLNVSHGQGLPPSLKDPADEAPGPVAPDSGDEGDSAPDTVVPANFNDPKQENDTDNTSGSAPLLPSFLHQLDYKFSGILALLGVVCIF |
| *Vitis vinifera* | VvPLC7 | GSVIVT01010003001 | IX | MGATFLLLAPIQPLKGPPPSSALKYQGIASHSHNFSFPLLAISTLEMSSEGRGSAGRAMLLIMVAVIYLLIQYSAPVHGATYTVGGSAGWTFNSANWPKGKRFRAGDVLAFNYDSKVHNVVAVNEGGYSSCTTPAGAKVYQSGKEQIKLVKGQNFFICNYAGHCESGMKIAVNAV |
| *Solanum lycopersicum* | SlENODL1 | Solyc01g014070.1 | II | MTVFRKQILLVVLVVVIAKLRSTTAVVYEVGDSWGWTFNYNYEQWAASKHFQLGDVFIFNYDPHLHNVRQVDKNYNDCTDHNPLASFNTGSDTLTLKTPGLYYFMCDIPGHCASGLRFQIKIDIPRVLPADPEKPSPPPPDPYESFPNIPGFPAKNPATSSNAGSLFKCTWSCKDMLLTFFLLALCF |
| *Solanum lycopersicum* | SlUC1 | Solyc01g090120.2 | I | MAVSAVATLLFLLVASPVAFAANHVVGGSGGWTQSGDYTTWAAAETFTVGDTLEFNYGGSHGVDVISKDDYENCNTGNAIESYSDGKTTIKLSKSGPMYFTCPTFGHCQSGMKLSINVLDSSTPSTPSTPSTPSTPSDSPATPSTPNVDNTPAKSTPTTPNGAAGVFGSMNKFVIGVSVVLGALFVFMC |
| *Solanum lycopersicum* | SlENODL2 | Solyc01g094040.2 | I | MAMAVVDSMVFLFLLPVALFFNLAASQSTGYTNHTVGGSAGWFFNIKTQKASADYSAWAAKQTFNLGDTLVFNTNTNQTVIQTYNATTYKNCTADYASDDDTFQYQGGSNEFGKAMTITVSLTLEGQQYYFSDADDGSQCLNGMAFEIKVGHGIGLPPSLNQPPPPPYVEPPSTVEDAESPPITVVTSSPNGCVRSSAGLFFAVSVLVMLALHLV |
| *Solanum lycopersicum* | SlENODL3 | Solyc01g104380.2 | II | MFGVSKSTIIVIVMILCILLQSNISNAATYPAGDGKGWGFNMNGWPNGKTFNAGDVIEFKYKVDEHNVVKVSQEEYDSCKTSGGQVFNSGDDQIPLEKGTSYFICTFGPHCSEGVKAAITAN |
| *Solanum lycopersicum* | SlENODL4 | Solyc01g104390.1 | VI | MYRVNKVTIVAFAMMLCIFLQTNISKADTFLAGGANGWGFQLNGWPNGKTFKTGDVIEFKYPAGAHNVVKVDQAGFNSCNGAGGQVFSSGDDKITLTKGTSYFICTIGQHCANGVKAAVIAN |
| *Solanum lycopersicum* | SlUC2 | Solyc01g104400.2 | II | MVFVLCIVVLIQAEITHAAVYNVGGTGGWTFNTVGWPKGKRFRAGDVLTFNYSSGAHNVVAVNKGGYNSCSTPRGAKVYNKGGDKVKLVKGQNYFICNFPGHCQSGMKISVFAM |
| *Solanum lycopersicum* | SlENODL5 | Solyc01g108350.2 | II | MALANAQSFLFLVVFSFMFVLSMANFNYGRGQGKLNKTNCPYGNNHPNATQTSNKFNVGGSENWRYGFDYMDWARKSGPFFVNDTLVFKYDAPNANGGFPHSVYLLPNYWSFIKCDLRRAKRIANPNQGVGEGFEFVLKKSQPYFFACGEHGGIHCNNGTMKFVVMPLKRWTF |
| *Solanum lycopersicum* | SlENODL6 | Solyc01g108360.2 | II | MALANAQSFLFLIVFSFMFVVSMANFNYTWAGGMKWNKTNCPYANNHPNATQTSNKFNVGGSENWHYGFDYMDWARKNGPFFVNDTLVFKYDAPNANGGFPHSVYLLPNYWSFIKCDLRRAKRIANPNQGVGEGFEFVLKKSQPYFFACGEHGGIHCNNGTMKFVVMPLKRWTF |
| *Solanum lycopersicum* | SlENODL7 | Solyc02g088380.2 | I | MRFDLMMIRSMMLLGLMKCAASGLDKVGGKHGWDQNVNYTDWASHQHFYVGDWLYFVFDKHYYSVLEVNQTNYEQCIDSDFISNITRGGRDVFHLTQARPYYFISSGGYCFHGMKLAINVEQPLPASPAPSSSNKTNASPPQIDKHLIISAFASSTLVWMFLFAQAT |
| *Solanum lycopersicum* | SlENODL8 | Solyc02g088390.2 | IX | MEAVRRRSFGWPTMAVVLTCALLVMLPQVYSIRYIVGSRFGWTNSVNYTNWAKDKHFYNGDWLFFVYDRNQMNVLELNKTDYESCNTDHPLHNWTTGAGRDVVPLNVTKTYYFASGKGYCYGGMKVAIHVEKAPPPPKAAPVRSSSTNLLNSFKAQIMIPALFATAAVWDAFLLL |
| *Solanum lycopersicum* | SlENODL9 | Solyc02g093650.2 | II | MALTCFGNVLFISIIVIITSSLELVNASSMEFQVGDTTGWTVPPQNDTIFYNNWASAMRFKIGDTIRFKYKKDSVMEVTDKDYKKCNSTQPHFFSNSGNTMFTLEHSGYYYFISGAAGHCERGQRMILRVMVQDLIPTASHAPFSFPIFQQLQLLFFLFLSLVLF |
| *Solanum lycopersicum* | SlUC3 | Solyc02g094050.2 | I | MVLVRKSLLISVIVMVMIQKNAMAAQHVVGGSQGWDESADFKSWASGQTFKVGDTLVFRYNPGLHSVVELEGDSSYKSCDISSTVNSMSAGNDVVKLNKPGTRYFACGTAGHCDQGMKLKIKTVTGSAPSNQEDSSTPRSSSAASHCFSTAFFTFIVAILTVQMALVFLL |
| *Solanum lycopersicum* | SlSC1 | Solyc03g116690.2 | I | MYKMMCVIVFGVLAVANMMQDATAQTVHVVGDTMSWIVPSNSAAAYTNWAAGKTFSVGDILVFNFMTGRHDVLQVEKTSFDGCNSQNAIGSAIMTGPANMTLNSTGEHYYICTFGMHCINGQKLAITVSNSTATPGANPPVVRQPEACAPTPSAAGPSNSVPGGRAPPPTPSSSTTVLASFVISLTAIALASLL |
| *Solanum lycopersicum* | SlSC2 | Solyc03g116700.2 | I | MDKMLYMIVFSALAITSMVHDTAAQTVHVVGDTMGWIVPSNGAVAYENWADDKTFRVGDTLVFNFTTGRHDVLQVQETSFDGCNSQNAIGTAIMTGPASITLNSTGDHYFICTFGTHCQGGQKLEISVSDDSTRTPGTNPPPRSVDGPTGSVPGGIVPPPPSSSTTLLASFLLSLSAIALVIFH |
| *Solanum lycopersicum* | SlENODL10 | Solyc03g117560.2 | I | MDLSVLFFLLLAAVATTVNATDHIVGANKGWNPGINYTLWANNQTFYTGDYISFRYQKSQFNVFLVNQTGYDNCTIDGALGNWTSGKDFIMLNDTQRYYFICGTGGCVNGMKVSVLVHPLASPPMLAVPAEHSSKKSSAPAARGFVSLLATSFAFFGLTLCSSVWM |
| *Solanum lycopersicum* | SlENODL11 | Solyc03g119880.2 | I | MTSSKAFLYLVFFSSFHFFTVFSTQFTVGGEKGWIIPKDDQLYNEWAAKNRFKVNDTLTFAYKKDSVLAVTQEEYEKCKSVHPIYFSNNGKSEFKLDRPGLFYFISGVSGHCERGLKMIVKVLEPASPPNQVADHTTGPSTSGAAQLINVVGVLVVSLFGAIFI |
| *Solanum lycopersicum* | SlENODL12 | Solyc03g120860.2 | I | MGSAFFYTTLCFIALTIVAASAEEFKVGDAVGWHQPSQNETDLYNHWAANKKFHIGDSLRFEYRNDSVIKVGKWEFYHCNRTHLASAGKDGTRTMNLNRAGTFYFVSGDPEHCKNGQRLAVEVLPLHPISESPPQPFSQAPAPSPLSISASVSSAVPLAFISALLISVIVVAIAGLA |
| *Solanum lycopersicum* | SlUC4 | Solyc04g074740.2 | I | MGKLLAVTFLFMCCVVPSLAKVYTVGDSSGWGLGVDYTTWASGITLNVGDSLVFNYPSGHTVDEVSSSDYSSCTTGNSITSDSSGATTIPLKTAGTHYFICGVMGHCSGGMKLAVTVAAAGGSGGATTPSTGTTTPTTGTTAPKTETTNTTHPSASVTLSPFIPLLISGVVAIFSYFVTI |
| *Solanum lycopersicum* | SlENODL13 | Solyc05g054900.2 | I | MAKPLLNFVVLILISLIICSSSATNYIVGDNSGWDISTDLDTWLLGKKFKIGDVLVFQYSSLHSVSEVTKENFMGCNTSNVLDSSKNGNTTFILTKPGDRYFVCGNRLHCLGGMKLHVNVENADGAAVRSPAAAPSPEGGASFSPSSKSNNPSFVPNFASSNIHVRLDSMILVILGLLLFTIPLV |
| *Solanum lycopersicum* | SlSC3 | Solyc07g008110.2 | II | MARKLSSLVVFGSILFALLQHVAMAQQTHVVGDTLGWTVPNGGAASYSTWAAGKSFVVGDILVFNFRSGSHSVAEVSKGAFDSCNTSSPISISTNGPTNITLSSAGSHYYLCTFPSHCTLGQKLAINVSGSASPAPQP |
| *Solanum lycopersicum* | SlSC4 | Solyc07g008120.2 | II | MARKLSTLVVVGAILFALIQHVSMAQQTHVVGDTLGWTVPNGGAASYSTWAARKTFVVDDILVFNFRSGSHSVAEVSKGAFDSCNTSSPISISTNGPTNITLSSAGSHYYLCTFPSHCTLGQKLAINVSGSASPAPQP |
| *Solanum lycopersicum* | SlSC5 | Solyc07g008130.2 | I | MEIILATKFGSSCSKLLLLVCLLFGCVVDVSLGRTYIVGDNLGWQTPPNGVVTYSNWANQHTFVVGDILEFNFNSGVHTATRVNKNAFDSCNAANPIDNETNGPAKFTLNTTGDYYFICTIHCNQGQKLTVNVTLTGSPSGSPTPGSSPSSPGGETSSPPSPSGSASTRVVASCVMLVPIVLALTLLA |
| *Solanum lycopersicum* | SlSC6 | Solyc07g008140.2 | I | MVTKMNIALVLMTILAALPGNIVAVDHIVGDTMGWTIPSGGPITYANWTSGRTFRVGDTLVFNFASGAHDVAKVTKSAYDSCSSTNPISLITVGPANITLNSTGSEYFICTFGQHCNAGQKLAINVATSSTTSPTPAPSPATSPAPTPVPNPTRAPTPTPSLSPSDGPSGPSPSPSGGAGDSPVSAPPPGPVTPEPTTPPPASPGDGLVPPPAPSSASRSVFVHALIMFMPIAISIMC |
| *Solanum lycopersicum* | SlENODL14 | Solyc07g008420.2 | II | MAISIGKLVFVCIFLILCVEMPTSLANEYVVGDKRGWSPGVDYHPWAYGKSFRVGDVLHFFYAPKVIDVASVDISSYALCDSNVKTFYKDNSGQTSITLDKSGPYYFISTSKKGCFEGLKLELHV |
| *Solanum lycopersicum* | SlENODL15 | Solyc07g052660.1 | I | MAMLRMLMSLAAITMLLGSAMATNYTVGSPNGGWDQSTDVQAWAASKTFFVGDNLIFSYSLSHSVLEVTKAGFETCQITAPIAIYTGGMTVITLASVGKKYFICGTGGHCNVGGMKLEINTLPKATPPPPPPAKPVTPPTPTPKAPPPSTPPPSTPKTPPPTPMTKPPVASPPSLSPSPKSSSAPSPRNIHKISPAMSPSKSSHAHSPAMPPTAVPTSPSVEAPGLPPSAEAPGLPPSAEGPDASAPATSSPSSADKISVVAGSTVGFGFVVMMMFLL |
| *Solanum lycopersicum* | SlENODL16 | Solyc07g053880.1 | I | MDSLSFVGFFFAFFLSFMCLSQGYTFYAGGKEGWVLNPSESYDHWARRNRFQVNDTIVFKYKKGYDSVYMVHDNDDYLKCNKTSPIHHLKDGHSKVIFTRNGPFYFIGGKDDNCEKGQKLLVVVMSPNHHKSSSPASSPVTIPTPPPESVSPAPAPAASAAIAVDVSPSSVLFGVFISLIAAAVFL |
| *Solanum lycopersicum* | SlENODL17 | Solyc07g063020.1 | II | MALQRHVPVLVVLLFFWSSSSNAYQFELGGVDDWIANPLESFDQWSQLMRIQVNDTLLFKNKKGSDLVLEVNEDDYDKCNIEHPIKKMEDENSVFEFDRSGSFYFVSGNKDKCKEGQKFVIVVAGMVLPPSTPVPSLAPVGSHLSPKADAPGKVPIGVSSPVPITVHVPRGTSSDSPQMSHSPGVAPTLASPGPSSSHLSPVSSAPEKDPVFGLSPAPTGSHSSSMGHAPGMAPTLVHVPRGIISNPPQTTHAPGIAPTLESSHGVPSASTHRSPIKAPSPSSFHVPSGSHLAPTALSPTSANVPLSSGSQLSPETHAPGKAPTGASLAPAGSHLSPTTHAPEKAQTPALSHVSPSPSSHLSPAAHAPAKSPTGASLGPSGSHQSPTVHAPGKAPTPASSNVDRARAPGKAPTGVSPVPASSHSSPTAQEPVEAPTPVSYVPRGIGFSPVAHAPGETPSGSSHAPDGSHSSPKAHAPIEAPTPIYNVPRGTSSHPPSTTHAPGKAPSESSHAPDGSHSSPKAHAPVEAPTPISNVPRGTSSHPPSTDHAPGKAPSEPSHAPDGSYSSPKAHAPIEAPTPMYNVTRGTSSQPPSTAHAPGKAPSESSHAPDGSHSSPKAHAPVEAPTPISNVPRGTSSQPPSTAHAPGKAPSKSSHAPDGSHSSPKAHAPVEAPTPISNVPRGTSSHPPSTAHAPVKAPSGSSHAPDGPHSSPKAHAPVEAPTLISNVPRGTSSHPPSTAHAPDSSTNAPIKAPTPVSSNVPRGTSSHPPSTAHAPGKAPSDSSPKAHAPIKAPTPKSSNVPRGTISNPPSITHAPVKAPSEPSPAPYGSHSTPKAHAPAKAGPTPAPSHVPSSPRSHLNPISNTPETAPTPITSKISPNPSSSLSPTSSHDEPSPSVSPTTAKSSNESSPISSSPSSSTPPRSGTDSPPENIAPATDGDNIPADIDSPANSPLNSESAAEKASITPSMSTMSVFFTLILGLIN |
| *Solanum lycopersicum* | SlENODL18 | Solyc07g064240.2 | II | MAQITSSRSNTKVFYFLGLFNILLMLQNANCYEFKVGGSGDWSVPMDANANNYNQWAERSRFQIGDTLSFSYSADKDSVLLVNKADFDNCNTASPIEKYSDGQSVVKFNHSGAYFFISGVHDNCVKNEKLHLVVMADRSHNNQTASSPPPSPSVDEVPPSPAPSGEEAPSPPSDSGETNQTPAPSQESSPPKNGASSTVMSFVGSAAALVGSSILLGF |
| *Solanum lycopersicum* | SlENODL19 | Solyc08g006790.2 | II | MAVFSRNVFVMAILFFSLLSFTEARDHLVGGKTNSWKIPSSESDSLNRWAEKTRFLVGDSLVWKYDGAKDSVLEVSKKDYVTCNTSSPIAMHNDGNTKIVLEHFGAYYFISGVKGSCEQGQKLVVVTLSKRRYADAPVPSPVEFDGPAIAPTSNGFSLKASLVVAFGVLVGFWV |
| *Solanum lycopersicum* | SlENODL20 | Solyc08g066740.2 | VII | MATLRNPRKYQSLIILFQFFLLIQIQLVFCYQYKVGDLNAWNIPSSANKDVYLKWSKNHIFKLGDSILFLYPPSEDSVIQVTKQNYNSCNLKNPILYMNNGNSLFNITRPGEFYFTSGAEGHCEKLQKLHISIGGGNGTSYDEADSPAFAPSPSYTNVFGSIPVQSSNSTSASSSGKVEISVFAAVGLLFLTLFIGSTIL |
| *Solanum lycopersicum* | SlUC5 | Solyc08g079780.1 | I | MASTSVMIAVIVVVVATPAMATDHWVGDDQGWKLDFNYTAWAATKQFHVGDKLIFKYKKDVHNVYKADHEAFKSCTPSSDVTPLTSGNDEISLASPGKKWYICGVGKHCEKGMKLAINVWPAESVSPAPSPSSPGSSSSSSFASSISPDSKFVALLVAAFAMIVMIMT |
| *Solanum lycopersicum* | SlENODL21 | Solyc09g065250.1 | I | MVLSKLTMFLSLMIFVSCGVSMGEVYKVGDSTGWTNNGHIDYKSWSSNKIFSVGDSIVFEYKQQMDNVVRVTHKNFNACNATTTYATYNTGNDTFVIKRQGHFYFISSFPGHCQNGQRVDIRVPKPTQLSPSPSPTPPRAPSSSSSPPPTETITPSSTQAPAPAPSGSSKMLFSINLWLSLSMLLIIVVAIV |
| *Solanum lycopersicum* | SlENODL22 | Solyc09g065260.1 | VII | MGFMKVAKLFLSITVVFSFFMFILASAAVYKIGDDAGWTFGSANVNYGVWAATKKFQIDDILVFVYDKTQNNVLRVSLSDFHNCNAANPIRSYSSGNDSITIMGPGHYYYICGFPGHCQTGQKVDIRVPKVVQPSDLPTGSPSPAPGTTLSTTSVHVTAPAPAKSNAPSFFINNGLGLGLGLTLFMIVIGADYVF |
| *Solanum lycopersicum* | SlSC7 | Solyc09g075810.2 | I | MAFCILENIPKMTLLFLILACFMQLCFGVVYKVGESAGWTTIGNVDYKQWAASKTFQVGDVIVFQYSPQFHNVMQVTHAEYQSCNASAPIATHTTGKDSITITAHGHHFFLCGVPGHCQAGQKVDINVLRVSSSVSPSQSPSSLSHIPAVAVPAPSPSHASYWLPSKTGLVLAVALVLLVSFA |
| *Solanum lycopersicum* | SlENODL23 | Solyc10g005940.1 | IX | MDTYFEFSLYRFNVFVLSLVVTFMIVSNIFVSSFQFRVGDEIGWITPIGNESETYNEWAARNRFHIGDTLYFKYKDDSVLEVTPANYLNCNTTSPISKFENGETVYKISHPGFYYFISGQKNNCKFGQRIIVRVMHPSEISSPASAPEISPSPAVDGGGGGGDGWSSDFLDPVINSTTVLSIFSCFVTALGGIMIFLYLLM |
| *Solanum lycopersicum* | SlENODL24 | Solyc10g007070.2 | I | MALHKHVIVILLACLINSCYAYQFYVGGRAGWVPNPSENYNNWAERMRFQVNDTLVFKYKKGSNSVLVVNKDDYDKCNTNNPIMKMDDGNSIFKFDHSGPFFFISGNKNDCQNGSQKLITVVLAIRPPPPSTPATSPATSPSVSPATSPVSQTPTSSPTISPATSPATSPATSPSTSPTTSPSTSPATTPAPASISPSGAIISPSPSGNSSSSSPSSPSTPGSPVESPPGGSGNSPPADIPAPTGSKNSAVKAYSTTSAVFVSILFTIIFFISV |
| *Solanum lycopersicum* | SlUC6 | Solyc10g037880.1 | I | MAFAAIFLFVLLASPIMCYGKDHIVGGSDGWSQSGDYSTWASAQTFNVGDNLVFNYGGSHGVNIVSKDDYDNCNTGNALLSYTGGQNSIPLSSAGDMYFVCPTLNHCDTGMKLAIKVEGTSSATTPATPYGTKSMPHNAASGTFGTMNKMVFGFSLALFVFMG |
| *Solanum lycopersicum* | SlENODL25 | Solyc10g050690.1 | II | MAFANGQAFLLLAVLFSSMFSIGLANHDFNWGPITWNKTNCPYSHPPNATQTSNKFIIGGSKNWHYGFNYMDWARNNGPFFVNDTLVFKYDPPNVNGTGFPHSVYLFSNYRSFIKCDFRRAKRIADPSEGAGEGFEFVLKKMQTYYFGCGEHKGIHCKTGNMKFAVMPLKHWRF |
| *Solanum lycopersicum* | SlENODL26 | Solyc10g050730.1 | II | MAFAYNAQAFPLLTLLFSSMFAIGLANYDFNWGSTTWNKTNFPYTHPPNATHTSNRFIVGGSENWHYGFNYMEWARNNTPFFVNDTLVFKYDPPNANSTGFPHSVYLFPNYWSFIKCDFRKAKRIADPTEGAGEGFEFVLKKMQTYYFGCGEHKGIHCKTWNMKFAVMPLKHWRF |
| *Solanum lycopersicum* | SlENODL27 | Solyc10g081520.1 | IV | MASKIIFCFMIGFLGFLPEITKATEYWVGGEKGWTLDVDYQAWAKDKTFKVGDTLVFKYNKDNHNVFKVNQTSFQNCIVPPPSQGLTSGHDVITLASPGKHWYICGFPTHCSDHSQKLVITVEAGVPAPAPAPLPATEYWVGGDKGWTIDVDYQAWAKGKNFKVGDTLVFKYTKGHHNVFKVNQTSFKDCIIPSSGGLTSGHDVITLATPGKKWYICGFPSHCSDHNQKLVITVEGEAPAPATPIPAPATPVPTPGPATKDDSNNSYRVTISAYKIFVGGVILIWTILTLV |
| *Solanum lycopersicum* | SlENODL28 | Solyc11g006440.1 | II | MKMQNHQNLLIKFSLLLIIILHHESYAMQHLVGDSIWTIPPTNNFYTNWSSSQVFFPGDTLYFQFDPEFYNVMQVSRREYGYCTSNQAYKVFNDGPLNITLIESGVFYYMCNILNYCELGQKFSVTVLQNSSNNYVPPPSTSS |
| *Solanum lycopersicum* | SlENODL29 | Solyc11g012130.1 | I | MGARIFLVVAIVFTAVATVVVAGAPVHHVVGEDRGWDPSTDITSWYSQRVFRVGDKIWFTYSTTQESLVELRNEEDFTSCDLSNPIKMYTDGLDTISLEGEGIRYFVSGNTESCKNGLKIPVKIQPKEQIMAQNNVGLMAVADGPTVPSASTNLNGLSYILAVGLSICLLGL |
| *Solanum lycopersicum* | SlENODL30 | Solyc11g062350.1 | II | MEQTIFNNRSLSCLVLLLILISFSTSVDAYKNYTVGDSLGWFDNLEKPLVNYDKWIANKKFSLGDFLIFNTDNNHSVTQTYNFTTYKNCDYNNALDNDTMQWVSADPSSTSIFPVTVAVPLLKVGPTYFFSSDYDGEQCENGQHFKINVTYGQGLPRDLKDPSDDDSMAPISPISGDEESAPDTIVPSSFDHPRDVSTDDSPEPSNSISLSMFSKVLGIQLNWVFVMLALVFGIC |
| *Solanum lycopersicum* | SlPLC1 | Solyc12g005710.1 | VII | MAANHTVGAPQGRWDQSTGLTTWAASETFLVGDSLIFVYTPNHDVLEVRKSDYDSCQTTNAISTNGGGMTIISLSSTGKRYFICGTGGHCASGMKLEVNTLATVPPPPVKSPVSAPSPKIAPKISPVSAPSPKNAPKISPVSAPSPKSLAPSAPKSSPPATSPETPSLSPSSTEFVPTSSPSSSPSSADKVSVIASSTVVGFGLVIMMLFFM |
| *Solanum lycopersicum* | SlENODL31 | Solyc12g010000.1 | I | MKNSNYSFLAYFLIISFFTNCFSEAREFIVDGKENSWKIPSSPNEFNKWAEKTRFRIGDYIVLNYDPKSDSVLQVNEEDYNNCNKAQPIKSYEDGVTKILLDRSGPFFFISGANGHCEKGQKLDVIVLSPKHSSSIRVKFLAPTLAPAPAPESGSSGMKVEIIGGFIVMLASIIVLA |
| *Solanum lycopersicum* | SlENODL32 | Solyc12g013900.1 | II | MAQQIHVVVLFMLACLFTTSYSYQFTVGGKDGWVLNPSVDYNTWSQHMRFIINDSVLFKYKQGADSVLEVSKDDYDKCNTGNPIKKMEDGNSIFTLDRSGPFYFISGNKDNCDKGQKLQIVVISARNQGKPPQTPAPAVAPPTPSGGLRTYVINAGSTPAAAPSKGSGTPSAPSANAPAGSSKPGASSPNGAPVSTPAGKSPTSSPTPSGSTASPPSPATVTPAMSPVANGPSTSTPGSSSPVAGGPSSGSGIAPSAGGPSGSAIAPAADGPTVSMSPGPSAGGPLAGGPSEGAPGSSALGPGGSNTPADINTPAGAPENPNSFAVKAFTPSVVLVSAVSLVLTVTLAEFIILP |
| *Solanum lycopersicum* | SlUC7 | Solyc12g042580.1 | I | MALHVFFLIVTTAIVATTISATDHIVGANKGWNPGINYTIWSNNQTFYIGDFISFRYQKTQHNVLQVDKVGYNNCTIEGALRNWSSGKDFILLDKSKRYYFICGIGGCSNGMKVSVLVHPLSPPPRSDAVSAVHSSEKSDAPVTFHGNFGSILVFVGLSYVCFRI |
| *Solanum lycopersicum* | SlUC8 | Solyc12g042780.1 | I | MEKIVCGALIIAILLQITIAQTEHIVGDSFGWSIPINGAAAYAMWADANSIKIGDTLVFKFTQGNHDVQEVSRSSYEECSTQNSIGEAIKKGPAKITVKTLGDHYYICTVGEHCLAGQKMAIKVTSSNSSASNTPAGSIPPPSSSSNIVFASFLLSLFSITFTIFL |
| *Solanum lycopersicum* | SlUC9 | Solyc12g056500.1 | I | MGLKALLVAIIVVNMVIVPTMSTDHFVGDDQGWKLKFDYNAWAESKEFHVGDKLIFKYKEGAHNVYRADLAAFQSCVPGANVEPLTSGNDVIDLKTPGKKWYFCGINNHCEQGMKVSVNVLEAKDGSSSSASRLSTLNSAFVAAFVMFLIVIA |
| *Glycine max* | GmSC1 | Glyma01g44940 | I | MAFIEKAVFFLMMMMTAFQVSHAAVHKVGDSAGWTIIGNIDYKKWAATKNFQVGDTIIFEYNAKFHNVMRVTHAMYKSCNASSPLTTMSTGNDTIKITNYGHHFFLCGIPGHCQAGQKVDINVVKVSAAAAPSPTSAMASPVPPANVPAPSPNNAAPFIVVKGAGIALMTLLALALSSYA |
| *Glycine max* | GmENODL1 | Glyma02g36580 | I | MASCLPNNASPFLFMLSMWLLISISEAAKYVVGGSETWKFPLSKPDSLSHWASSHRFKIGDTLIFKYDERTESVHEVNETDYEQCNTVGKEHVLFNDGNTKVMLTKSGFRHFISGNQSHCQMGLKLMVVVMSNNTKKKLIHSPSPSPSPSPSPSPSPSPSPSPSLSSPSPSPLPNNQGVTRSSGAEFIGVMMWLGVMMLLL |
| *Glycine max* | GmUC1 | Glyma02g37210 | I | MAISVALILGLCLAMNMALPTGAATHTVGDTSGWALGVDYSTWASGLKFKVGDSLVFNYGTGHTVDEVKESDYKSCTMGNSLSTDSSGATTITLKTAGTHYFMCAAPGHCDGGMKLAVKVKAKNASASAAAPSPAKESPSESDDAKDTPTTPTSTTNPKTSTSTTPSTETSTTTTSSYTSSAIGGSPIVAMFVVSWISYLLLMM |
| *Glycine max* | GmENODL2 | Glyma02g44285 | IX | MAKSELHYVGGDKSSWGPNVNLTEWSSHEHFHLEDWLYLMMCLQDFGYDRNEYNVLEVNKTGYENCVDTGFVQNISRGAGRDVFHLTEFKTYYFLSGGGYCWHGMKVAISVTEGVSAPNPATSPKGGAQASSPKSGCASDGIQVNQKLLEFIFVLMWSISSINISY |
| *Glycine max* | GmENODL3 | Glyma02g44300 | I | MEILSFKKIVMMMITMMLVNMAKSELHYVGGNKTTWAANVNFTEWSSSEHFHLMDWIYFGYERHEYSVLEVNKTSYENCIEKGFIQNVSRGAGRDVFQLTEFKTYYFLSGGGHCWDGVKVAITVTEGVASPTPAPSPKTGAPTPSPQSDVPATSPKSGAPAPSPKTSSASDDNRVNQMLLVFIFVLICGISSNNISY |
| *Glycine max* | GmENODL4 | Glyma02g44580 | I | MERGRRSSSFGPMSSTVPCFLLLLFCLSGSAEAYKNYTVGDSLGWFDNTEKSNVNYQKWADSKEFSLGDFLIFNSDTNHSVVQTYNFTTYKECDYDDAQDKDTTQWSASDPSNTQTHPVTVAVPLVKEGMTYFFSGDYDGDQCKSGQHFKINVTYGQGLPKSLKSPEDAPSPASPVAGDDDSAPDTIVPSNFSHPKEENDDDKASDDQKSSSSVSILMHAQLHIVLLGILLFLF |
| *Glycine max* | GmUC2 | Glyma03g26060 | I | MASAIAASFLVLLLAFPTVFGADHEVGDTSGWALGVNYNTWASGKTFTVGDTLVFKYDSTHQVDEVDESGYNSCSSSNSIKNYQDGNSKIELTSPGKRYFLCPISGHCAGGMKLQINVAAASGTPPTTPSGTPPTTPSNPSPSPPSESGSTNTTSPPKPSGAVTVSSGIGLLVGSFFVSAIMFGFMG |
| *Glycine max* | GmENODL5 | Glyma04g03900 | II | MDATRILAQGVFVFLVITYVGAEREPRTILVGDSQGWQAGTNYTQWAIQNSPFHINDTLVFKYPGNSTTLAQSVYLLPNQWSYITCEFRGAKLLGNATEGDGEGFKVELNQLTPYYFASAEGNFYDCIAGLSKFIAVPSTSSGSS |
| *Glycine max* | GmENODL6 | Glyma04g06410 | VII | FLPCSSSHHKCNTPNTPTNANPPLCACVCKKGKSFHTPFSLLKKQKAMAGSSASLLFLFLLFGFSAAKELLVGGKIDAWKIPSSESDTLNQWAERSRFRVGDHLVWKYESGKDSVLEVTREDYANCSTSNPIKEYNDGNTKVKLEHPGPFYFISGSKGHCEKGQKLIVVVMSPRHTFTAIISPAPTPSPAEFEGPAVAPTSSATTFQVGLLTALGVLAIYVGFLM |
| *Glycine max* | GmPLC1 | Glyma04g10670 | IX | MTVFKYGGGGGHTVDEVKESEYKSCTAGNSISTDSSGETTITLKTAGTHYFICSVPGHCSGGMKLVVTVKSGKATDSSSTSTGKASPSDVTPNTTKSNSSSASGVSPIVAMFIVSWISYNVLYDLSEY |
| *Glycine max* | GmUC3 | Glyma04g42120 | II | MSKVAVIMREEAVHLYLLWSLVSLLCLLVLLERADAATYTVGGPGGWTFNTNAWPKGKRFRAGDILIFNYDSTTHNVVAVDRSGYNSCKTPGGAKVFSSGKDQIKLARGQNYFICNYPGHCESGMKVAINAV |
| *Glycine max* | GmENODL7 | Glyma05g07791 | I | MAIPYKASSPLFLCLILSCGDSLVNCSEFEVGGHDGWVVPKPKDDDQMYNQWASQNRFKVNDTLLFKYERDSVMVVTEEEYEKCKASRPLFFSNNGDTVFKFDRPGLFYFISGVSGHCDRGQRMIIKVLDVEPAAPPPQSANEDAQKPPHKKNGVAEMIPMSIITTSTLFVLSTFVLQLYA |
| *Glycine max* | GmUC4 | Glyma05g14800 | I | MGHKNTIFLALIATLIAKEAFAAQHVVGGSQGWDQSTDFKSWTSGQTFKVGDKLVFKYSSFHSVVELGNESAYKNCDISSPVQSLSTGNDVVKLDKPGTRYFTCGTLGHCSQGMKVKITIRKGNAPSPALSPATSPSLSPSLSPLLSSTSLSSPPSSSSPTNTNASSASQCFTSFMFIVALSVTIMVSLF |
| *Glycine max* | GmUC5 | Glyma05g30380 | II | MALGRGSAVVLLLCFLLLHSQMARAATYTVGDSGGWTFNTVAWPKGKLFRAGDTLAFNYSPGTHNVVAVNKAGYDSCKTPRGAKVYKSGTDQIRLAKGQNYFICNYVGHCESGMKIAINAA |
| *Glycine max* | GmENODL8 | Glyma05g37110 | VI | MAVKMHLSFFILATIGCMPLVSVSSTTHVVGHKLGWNLPSYPGFYDDWAKKQTFVVGDVLLFQYHPGQNTVVQVDKNDYDHCTTRNILHTYFRGNSSATLEKPGDYFYFSSVGKHCDFGQKLHVTVSQ |
| *Glycine max* | GmENODL9 | Glyma06g04000 | II | MDATRILAQGVFVFLVITCVGAESEPRTILVGDSQGWQAGTNYTQWAIQNSPFHINDTLLFKYPGNSTTLAQSVYLLPNQWSYITCEFRGAKLLGNATEGDGEGFKVELNQLKPYYFASAEGNFYDCIAGLSKFIAVPSTSSG |
| *Glycine max* | GmENODL10 | Glyma06g06450 | I | MAACSRTSLLLLFFLFGFSAAKELLVGGKIDAWKIPSSESDSLNQWAERSRFRVGDHLVWKYENGKDSVLEVTREDYANCSTSKPIKEYNDGNTKVKLEHAGPFYFISGAKGHCEKGQKLIVVVMSPRHIISPAPSPTEFHFEGPAVAPTSSATTFQVGLLTALGVLAMYVVFLK |
| *Glycine max* | GmUC6 | Glyma06g10500 | I | MAFSSALILWSLLAINMALPTLATVYTVGDTSGWAIGTDYSTWTGDKIFSVGDSLAFNYGAGHTVDEVKESDYKSCTAGNSISTDSSGATTIALKSAGTHYFICSVPGHCSGGMKLAVTVKSGKASDATPTTSTTTATKSNSSSASSVSPIVAMFIVSSISYYVLRML |
| *Glycine max* | GmUC7 | Glyma06g12680 | II | MSQGRGSASLPIVVTVVSLLCLLERANAATYSVGGPGGWTFNTNAWPNGKRFRAGDILIFNYDSTTHNVVAVDRSGYNSCKTPGGAKVFSSGKDQIKLARGQNYFICNYPGHCESGMKVAINAL |
| *Glycine max* | GmSC2 | Glyma06g26610 | I | MARKVGLNFIGCSIVAMVFIIGVAEATDYIVGEGFGWSVPSNESFYTDWASTKRFFVGDNLIFNISGEHSVGIRTEATYYENCNTSLLTGFTFIGVNGSNSMFRHNIIPPTGPRYFLCTVGNHCERGQKFSISVESHPDSAAPTTLSFRILSAFLSSLAVYFFTVTS |
| *Glycine max* | GmSC3 | Glyma06g28650 | I | MGQLHNIMVILIVIAVAATMLKSTKAAEYTVGDNTGWTSAPPGGASFYSDWASNITFREGDILVFTFTASHTVAELTDRASFDGCSVNQNQGVITTSPARITLNRTGDFYFACTIQGHCNSGQKLSIATITSTSSPPTQGPSPPSGTTPTPPSSGDETPPPQSPPTEPGSTTPPPSRGEATSLVATFSILLITLLINSLLF |
| *Glycine max* | GmENODL11 | Glyma06g36590 | I | MEFQKPLWLFLLLLTLLSSSQANKFNVGGSKGWVPNPSESYNNWAGRNRFQINDTIVFKYNKGSDSVLEVKKEDYDKCNKTNPIKKFENGDTEFKFDRSGPFYFISGKDGNCEKGQKLIVVVLTPRTPPTPKTPPFPKTSPSPSPSQSPKANPPTVSPPLPSPSPSVHSKSLSPASSPVPAVGTPAISPAISIPTLAPETGTPPPSLGPSSSSPPSPGPSSSSPPSPGPSSSSPPPAGPTSPPSLAPSSNSTAPSPKNSGFSVAPSSVLMYCVTIVAGAALLRY |
| *Glycine max* | GmENODL12 | Glyma06g42110 | I | MATFILRSNKAVHAFGWLCLLLMVQRGASYEFVVGGQKGWSVPNDPSFNPFNQWAEKSRFQVGDSLVFNYQSGQDSVLYVKSEDYASCNTNSPYAKYSDGHTVIKLNQSGPHFFISGNKDNCNKNEKLTVIVLADRNKNTNQTTTTNASPPSPQSSSSSPSPAPTGQYQGQSPTSDTNQTPSPISEPPPPPNAAASAFVSLAGSVGAFMASVLVLSL |
| *Glycine max* | GmENODL13 | Glyma06g44550 | I | MAIFHRFLGLLILMAPMLLLHVVARQFDVGGKDGWVLKPTEDYDHWAQRNRFQVNDTLHFKYNKGIDSVVVVKKEDFDSCNINNPIQKMDGGDSTFQLSNSGLFYFISGNLNNCKNGQKLIVLVMAVRQPISKAAPPPASILPPQKIPATDLTSPAPTPTTDNSGSGRVGVSVSVGIVFMFIAFVGLV |
| *Glycine max* | GmENODL14 | Glyma07g02500 | I | MGGSWSWQLTCALLLLFSAVVTATDHIVGANRGWNPGFNYTLWANNHTFYVGDLISFRYQKNQYNVFEVNQTGYDNCTTEGAVGNWSSGKDFIPLNKAKRYYFICGNGQCFSGMKVSVVVHPLPPPPTSAVAAQHSSPKSASPVLLKRGFRSLLVSTVSACFGIVWIYHL |
| *Glycine max* | GmUC8 | Glyma07g13840 | I | MARAIAVSFLVLLLAFPTVFGADHEVGDTGGWALGVNYNTWASGKTFRIGDNLVFKYDSTHQVDEVDESGYNSCSSSNIIKNYKDGNTKIELTSTGKRYFLCPISGHCAGGMKLQINVVAGTPPTTPSGTPPTTPSNPSPSPPSDSGSTNTTSPPKPSGAVTVSSGIGLLVGSFFASAIMFGFMG |
| *Glycine max* | GmENODL15 | Glyma07g29400 | II | MALKLGHGVMILVIISASMFSVSMANKDWSFGFNYTDWWSRFGNHPQNKTQQQPKQIVVGGSEHWHYGFNYTDWAFKSAPFYLNDTLVFKYDAPNATSFPHSVYMFKSFGSFLKCDIEKAKMLANPMQGTGEGFKFVLKRWKPHYFACGERNGFHCNNGTMKFAVMPMIRPFWQWP |
| *Glycine max* | GmENODL16 | Glyma07g33210 | IX | EIIVGGSEHWHFGYNYTNWAINNGPFYFNDTLVFKYDAPNATSFPHSVYLLANFWSFLNCDVKKAKMLANPKQGAEEGFKFVLKKWQPYYFACGERNGFHCNNGQMKFAVIPMLRPFWPWP |
| *Glycine max* | GmUC9 | Glyma08g13510 | II | MALGRGSAVVLLLCFLVLQSEMARAATYRVGDSRGWTFNTVTWPQGKRFRAGDTLAFNYSPGAHNVVAVSKAGYDSCKTPRGAKVYRSGKDQIRLARGQNYFICNYVGHCESGMKIAINAA |
| *Glycine max* | GmENODL17 | Glyma08g19710 | II | MAQGRGILLLLCMLVLYSVSEMAHAKTYMVGGEFGWNYTVNMTTWPNGKSFRTGDILVFYYITYDNVVIVDEAGYNSCRAPKGSITYRSGNDHIALARGPNYFICTNQDHCSLNGMKIAVNAI |
| *Glycine max* | GmSC4 | Glyma08g22680 | I | MNKMTGLALGGVVAAILMVLQYAEAQTSYVVGDGTGWRVPQDASTYQNWASDKNFTVGDTLSFIFQTGLHNVIEVSEESYNSCSSANPIGTTYNTGPANVTLNRGGEHYYICSFGNHCNNGQRLAITVSGSSTAFPPATTTAPPPPSSSAPSSFHTTVLPFLFLSAFVFIAF |
| *Glycine max* | GmENODL18 | Glyma09g01250 | I | MKMGKGIVFIVALVAICLGGKWVEAQVHHVVGADRGWDQTSDLVSWSASRVFRVGDQIWLTYSVAQGLVAELKSREEYEACNVSNPINVYTEGLHTIPLESEGMRYFVSSEPENCKNGLKLHVEVLPKADERITEPSTSTLTDEAVAPTTPSGSPRYAHNTMLKLTVILFLIIGLAY |
| *Glycine max* | GmENODL19 | Glyma09g29566 | II | MEKLRPAWAVKAIIVIVFTSILFRCVCGENHTVGGASGWDLRSNIQAWSSTTTFNIGDDLVFSYTPVHDVVEVNQLGYNTCTIANALATYDNGETVIHLSDAKTRYFVCGRMRHCQQGLKLQVQILAQSNNGTSDDQNQSPGAGTGTVGSPPSPSPPPPPPPPPPPPPPPVSPPPHGDEQPPADEPVPCDCNRAEERHGVVPLITLVIVVALAHAPFFIAFPHLRFHLMR |
| *Glycine max* | unknown | Glyma09g29621 | IX | MGLQAYQCSCLIMFYLCLTNPFIFLYAKGETYIVGDSYGWDDVLDFSNWRDGKEFHVGDVLVFNYERSLHSVLQVDSTAYENCIKDSYTQRFTSGNDSVVLKEGRAWFICGVDDHCENGQKLHITATS |
| *Glycine max* | GmENODL20 | Glyma09g40300 | I | MAPPIAAQLLAFALLVTAVSAAETGYHNHTVGGGTGWSFNSTTNTSATNYSSWASTQTFDLGDYLIFNTNSNQTVVQTYNKTTYLNCTADYSDNGTFVYNGGSRGFGEALTVVVPLTIVGPNYFFSDAGDGVQCQRGLGLPPSLNQPPPPPYQEPPGPDAAQSPPITVAQSPSGSAFANLADLRVVVYGIVLGLLLQFL |
| *Glycine max* | GmENODL21 | Glyma10g31640 | I | MANFSSHYGMLVSLLLTWIQIQAKVFCYQYKVGDLDAWGIPSSENPQVYTKWSKYHNLTIGDSLLFLYPPSQDSMIQVTEESYKSCNIKDPILYMNNGNTLFNITSKGQFFFTSGEPGHCQKNQKLHVAVGEGIMETMDTAPGPSSSLPASAPSYPTVFGNIPVAPSTSTSTQLISTSQLLIIGFVICALFSSLM |
| *Glycine max* | GmENODL22 | Glyma10g33720 | II | MEKLLLVYSLLFSVVIITCSATTYTVGDSSGWDISTNLDAWIADKNFRVGDALVFQYSSGQSVEEVTKENFNTCNTTNVLATHGNGNTTVPLTRAGDRYFVSGNKLYCLGGMKLHAHVQGDDKSLAPTLAPKAVAGSDQNTATLPQSPSSKKNTHLSAGAANPARDALHLVYIAPMAAIYGMMKI |
| *Glycine max* | GmENODL23 | Glyma10g33930 | I | MLQHQNPFVLSFSALFLAFFCHCSATTFTVGDSAGWIIPPYPTYYNNWSHSQFIRVGDSVEFKFDDKFYNLIQVSQKEYEHCTSLEPLRIFNTSPVILPLRERGVMFFICNIPNYCCLGQKIVISVHEGSIEKPPSPSPSPSQVPINISPQPSPNASAPQPHGSSGMSSPPSPTTNTSGGNGGNSPVPSSTQEGKSNAVALVGGKSFTVSLGQLLSVLGALFGFWIM |
| *Glycine max* | GmSC5 | Glyma10g39530 | II | MALVERVVVLFIVMAFVQVSFAAVYKVGDSAGWTTLGTIDYRKWAATKNFQIGDTIIFEYNAKFHNVMRVTHAIYKTCNASSPIATFTTGKDSINITNHGHHFFFCGVPGHCQAGQKVDINVLSISAEAPTPSGSALASPTVQTSTVPAPSPSNATPLISLKGSFGSVGFNMTVFCLVWFMYSFF |
| *Glycine max* | GmPLC2 | Glyma10g42840 | IX | MLSYIFKDMGETRFLKNCTFLMAILTLLSSFLLNHVMASVYTVGDQEEWSSQTNYASWAERYNFSQGDVLVFKYVKGQHNVYEVTEDTFRSCDASSGVLAKYESGEDQVALSEVKRHWFICNIAGHCLGGMRFGIEVKDGNSVTNSTDVAFNPPIEPTPSHNSCTCYYVSERWRVIENFIPLGLLLLLNYYF |
| *Glycine max* | GmSC6 | Glyma11g00700 | I | MAFIEKAVVFLMMTAFQVSNSAVHKVGDSAGWTIIGNIDYKKWAATKNFQVGDTIIFEYNAKFHNVMRVTHGMYKSCNASSPLTRMSTGNDTIKITNYGHHLFLCGVPGHCQAGQKVDINVVKKVSAEAPTPSPISAMASPVPPAPSPNNGAPFIVVKGALIMPLLALALSSYASLGISLLVN |
| *Glycine max* | GmENODL24 | Glyma11g21790 | IX | MTIVKVSYEVIYKVGDSAGWTTLGTIDYRKWAATKNFQIGDTIMLNQYIIMFSTQLSKKKIMFSTPNQKEATTSRLSGADGDTKQR |
| *Glycine max* | GmENODL25 | Glyma11g34500 | II | MLVFIATIFLPSIAMAKKFVVGFDYAAWAADKTFQVGDVLVSSMMLENTVFKVNGTAFQSCTTPPASEALSNGNDRIVLAIPSRKVLLVTAMLAKSLLLQFNPKHCLQLYHHHLHLVSIALILVDGCLLFFIKLHLLV |
| *Glycine max* | GmSC7 | Glyma11g34510 | II | MALSPLGMLLVIATILLPFNIVVAKEFVVGDDHGWTIGFDYAAWAADKTFQVGDLLVFKYAVGKHNVFKVNGTAFQSCTIPPASEALTTGSDRIVLAIPGRKWYICGVVGHCNAGQKLVITVQPQTLPPTPAPAPSPFQHRPHFGRWIPKKLFTIFH |
| *Glycine max* | GmENODL26 | Glyma12g13130 | I | MAFHRFLGLLILMTPIMFVQVVARQFDVGGKDGWVLKPTEDYDHWAQRNRFQVNDTLHFKYNKGSDSVVVVKKEDFDSCNINNPIQKMDGGDSTFQLSNSGLFYFISGNLDNCKNGQKLIVLVMAARQPIPRAALPPQKIPATSLTSPAPTPTDNSGSGRVGVSVGIVLMFTGFVGLV |
| *Glycine max* | GmENODL27 | Glyma12g16340 | I | MATFILRSNKAVHAFGWLCLLLMVHKGASYDFVVGGQKGWSVPNDPSFNPFNQWAEKSRFQIGDSLVFNYQSGQDSVLYVKSEDYASCNIDSPYAKYSDGHTVYKLNQSGPHFFISGNKDNCNKNEKLTVIVLADRNKNTNQTTTASPPSPQTSSSPSAAPTGQDQGQSPTSDTNQTPSPVSEPPPPNAAASAFVSLAGSVGAFMASVLVLSL |
| *Glycine max* | GmUC10 | Glyma12g32270 | I | MGVPELMFRVSFMAVLIKLASATNYIVGGPSGGWDTNSNLQSWASSQIFSVGDSLVFQYPPNHDVVEVTKADYDSCQPTNPIQSYNDGATTIPLTLPGKRYFICGTIGHCSQGMKVEIDTLASATNSVTPAASPEDSTTSPAESPEVIISSSPSPLFQTHLESPTFSPVIPSTEFPASTSPLAQHSSDFSASSTSKGNLQAYVAVVLSFLIMLMSF |
| *Glycine max* | GmENODL28 | Glyma12g34100 | I | MATIILRSNEVVHALGLFCILLLVHKGDAYEFVVGGQKGWSIPSDPNSNPYSQWAQKSRFQVGDSLVFNYPSGQDSVIQVSSQDYASCNTDAYSQKFSDGHTVINLNQSGPHFFISGNKNSCLKNEKLVVIVLADRNNKNTNQTSPPSPNCPSPSPSPSLSTQSLAPSPAPSQQVAPPPLSPVAAPPQQEAPSPPSPATNNPTPAPVSDQPSPPSPPHNAASSILVNFACSVGAFIASVLVFSF |
| *Glycine max* | GmENODL29 | Glyma12g35411 | II | MELEIRACLLLFLFSLFSCSQAYTFYVGGKDGWVLYPSENYNHWAERMRFQVSDTLVFKYKKGSDTVLVVNKDDYEKCNKKNPIKKFEDSESEFQFDRSGPFYFISGKDGNCEKGQKLIIVVLAVREPPPYYPPTPPNTPYVPITPPKAPYVPTPPSNSSKNSLSTLCSNPSKNTFSYSQSSLCSS |
| *Glycine max* | GmENODL30 | Glyma13g05790 | IX | MENWRGARLMVVASVVAIGWLSLVVMGSPVLHKVGGSKGWINHDVNYTEWAAQEHVYVGDWLIFKFDRRYFNVLEVNKTSYENCIDRDFIKNITRGGRDVVQMTEARTYYYLSDGGYCFHGMKVAVQVQEYQDPALAMVAPAPSPVVSGSSVFTCIWIIVANVVLFVNLMVVGIL |
| *Glycine max* | GmENODL31 | Glyma13g05810 | I | MERMKRVLLLLLAFTVLLMLPEASATKFTVGNNQFWNPNINYTEWAKGKHFYLGDWLYFVYDRNQASVLEVNKTDYETCNSDHPLTNWTRGAGRDVVPLNVTKTYYIISGRGFCFSGMKIAVHVEKLPPPPKAAPVKSAAPTLFSQDRILLMPVVFAIGAAWDAFIHFW |
| *Glycine max* | GmENODL32 | Glyma13g10460 | I | MANLSSPGFNLFLVSLLVTLVQIQTKVQCYQYKVGDLDSWGIPISPSSHLYDKWSKYHNLRIGDSLLFLYPPSQDSVIQVTAESYKSCNLKDPILYMNNGNSLFNITSEGDFYFTSGEAGHCQKNQKLHITVGVGGNTNALAPTSLPENATSYPTVFGNIPMAPSSSRSTSSSPHRTSKFSLLIIGFFLCALLSSLM |
| *Glycine max* | unknown | Glyma13g22650 | X | MALSTYPPFPYKCKQKALAFNVTSSFTGRRTRRKNRHKDLVLARIKQGRSHLQEHIAMARNLLLVLFAVATLLHGSAAQTRHMVGDATGWIIPAGGAATYTAWASNKTFTVNDTLVFNFATGQHNVAKVTKSAFDACNGGSAVFTLTSGPATVTLNETGEQYYICSVGSHCSAGQKLAINVNRASSTGPSPAPQPRGSGSPPRASPVPTQAPQASSPTPPPRSAPAPAFGPSSEPATFIVGETAGWIVPGNASFYTAWASGKNFRVGDVLVFNYASNTHNVEEVTKANFDACSSASPIATFTTPPARVTLNKSGQHFFICGIPGHCLGGQKLAINVTGSSTATPPSAAAPPTTPSSPSPAGAVTPPPQNSGAASLGVVGVFATLLSVAATFFY |
| *Glycine max* | GmENODL33 | Glyma13g23800 | I | MVKKMAQGRSSAATMLLLCMLVLYSEMVHAATYVVGDATGWAYNVNNWPNGKSFKAGDILEFKYSPFAHNVIQVDEFGYNTCIPTFNSRLFFSGDDHIQLAKGLNYFICGFPGHCQLHGMRIAVNATA |
| *Glycine max* | GmENODL34 | Glyma13g35100 | II | MELEIRVCLLLFLFGILSGSQAYTFYVGGKDGWVLYPSENYNHWAERMRFQVSDTLVFKYKKDSDTVLVVNNDDYEKCNKKNPIKKFEDGDSEFQFDRSGPFYFISGKDDNCEKGQKLIIVVLAVREPPPYYSPTPPNTPYVPITPPKAPYHPNPPYVPTPPNTPYVPITPPRAPYVPAPPQTPTVPTPPETPYHPPYVPIPPPKTPSPISSPPYVHPKPPSPIHYPPNVPTPPKTPSPVYTPPNVPTQAPSPANHPPYIPTPPQTPSPVYTPPNFPTPPKIPSPVYTPPKAPSPSNQPPYVPTPPKTPSPSYNPPNVPTPPKTPSPIYTPPNVPTPPKTPSPAYSPPNAPSPHNQPPYVPTPPKTPSPSYNPPNVPTPPKTPSPIYTPPNVPTPPKTPSPAYSPPNAPSPHNQPPYVSTPPKTPSPIYSPPNVPTTTPKNTSPWQPTLCPNSLKHSFSYFSTSLHSNPSKNAFPCPSTTLCSNNPISNISTTLHSHTSKHHSFTHFPTXXXXXXXXXXXXXXXXXXXXXXXXXXXXXXXXXXN |
| *Glycine max* | GmENODL35 | Glyma13g36441 | I | MATIMLRSNEVVHALGLFCILLMVHKGDAYEFVVGGQKGLGLFNYPSGQDSVIQVRSQDYAIIKLNQSGPHNFISGNKNSCLKNEKLVVIVLADRNNKNTNQTSPPSPSSPAPSPSLLTQSLAPLPAPSQQQVPPPPSPVAAPPQQEAPSPATSNPTPAPVSDQPSPPHNDASSILFNFAGSVGAFIASVLVFSLSGVYDI |
| *Glycine max* | GmENODL36 | Glyma13g37160 | VII | MRYSNDGFLGLFALTIISIFILGSSTSSYTFRVGGKDGWVINPSEDYIHWPQRNRFHVNDSLYFKYKKGSDSVLVVNKDDYDSCNSNNPIQKMDEGDSLFTLDKPGPFFFISGNLENCQSGQKLIVVVLADTHEHSQSPSQPEAEAPIANWPSGPNSPVASPPKHSGSTSVRGLRVALVSLSVLVLRTWGGFALQQVGLV |
| *Glycine max* | GmUC11 | Glyma13g38150 | I | MGVPEMMFRVSFVAILIKLALATNYIVGGPNGGWDTNSNLQSWASSQIFSVGDSLVFQYPPNHDVVEVTKADYDSCQPTSPIQSYNDGTTTIPLTSLGKRYFICGTIGHCSQGMKVEIDTLASATNSVTPAASPEDSTTSPAESPEVSSASPEESPEDIIPSAPSPLFQAHLESPTFSPVFPSTEFPASASPLAQHSSDLSASSTSKGNLQAYFVVVLSFLIMFITF |
| *Glycine max* | GmENODL37 | Glyma13g43190 | I | MASRLFVLSTCVIIFMAATNTCVEASVQFKVGGSFGWHEPAGTNNTDQLYIQWAERNRFQVGDALVFEYQNDSVLSVEKFDYMNCDASNPITAFDNGKSTFNLDRPGNFYFISGTDDHCKNGQKLLVDVMHPHTVLKSPPPISLPPEGFPPMAPPPSDDQSLEASSASVLLTFMFMSLFVTSVSVMLLAF |
| *Glycine max* | GmENODL38 | Glyma14g04140 | I | MEQGRRRRSSFGPMSSTIPCLLLLLFCLSGSAEAYKNYTVGDSLGWFDNTEKSNVNYQKWADSKEFSLGDFLIFNTDTNHSVVQTYNFTTYKECDYDDAQDKDTTQWSAADPSNTQTHPVTVSVPLVKEGMTYFFSGDYDGDQCKSGQHFKINVTYGQGLPKSLKSPEDAPSPASPVAGDDESAPDTIVPSNFSHPKEESDDDKASDSDKQKSSSSISISMYAQLHIVLLGILLFLF |
| *Glycine max* | GmENODL39 | Glyma14g11760 | I | MAGYSKTSTTTSASLLILFLLFGFSMAKEMLVGGKTDAWRVPASESDSLNQWAEKSRFQVGDYLVWKYDGGKDSVLQVSREDYGNCSISNPIKEYNDGTTKVKLEHPGPFYFISGAKGHCEKGQKLVVVVLTPRGGRFTGIAPSPSPSPSSAFAEFEGPAVAPTSSATALNGGILMMAMGVVAAMWVFPM |
| *Glycine max* | GmUC12 | Glyma14g35530 | I | MASSVALVLGLCLALNMVLPTRAATHTVGDTSGWALGADYSTWASGLKLKVGDSLVFNYGAGHTVDEVKESDYKSCTTGNSLSTDSSGTTTITLKTAGTHYFICASPGHCDGGMKLAVKVKAKKASAPATAPSPAAKDSPSDSDDTKDTPTSTSTNPKTSTSTTPSTETSTTTTSSYTSSATAAGSPIVAMFFASLIPYFVLRLM |
| *Glycine max* | GmENODL40 | Glyma15g02160 | I | MASRLFVLCTCVIIFMAATNTCVVEASVQFKVGGGFGWHEPAGNNNTDQFYIQWAERNRFQVGDALVFEYQNDSVLSVEKLDYMNCDASNPITAFDNGNSTFNLDRPGNFYFISGTDDHCKNGQKILVDVMHPHSILKSPPPISLSPSLPPEGFPPMAPPPSDDQTLEASSASVLLTTIFMSLFVTFVSVMLLAF |
| *Glycine max* | GmENODL41 | Glyma15g12080 | I | MKMGKGMIFIVALVAICLGGKWVEAQVHHVVGADRGWDQTSDLVSWAAGRVFRVGDQIWLTYSVTQGLVAELKSKEEYEACDVSNPIKMYTEGLHTIPLESEGMRYFVSSEPENCKNGLKLHIEVLPKADERIIESSTLTDEAVAPTTPSGSARYGHNTMLMLTVILFVVIGLAY |
| *Glycine max* | GmSC8 | Glyma15g40431 | II | MGFNLIGSSIFAMIFIIGVTEATNYTVGDSFGWSVPSNKSFYTDWASTKKFFVGDNLIFNWNGNHSIKITMKTIDYESCNSSWFDALTYLVTKNGTAMSIHTIDAPIGYRYFYCIVGNHCELGQKFSINVQSHSGSSPAPAPAPSFGILSAFLSSLAVYFFTLTRVLVDLEDNLKI |
| *Glycine max* | GmSC9 | Glyma16g04260 | I | MASAARLTFFAVSMVLLSSVAIATDFTVGDGTGWTLDFNYTAWAQAKLFRVGDTLWFNYDKTKHNVVKVNGTEFQECSFTANNEVLSSGKDSIVLKTEGKKWYVCGVGNHCAAHQMKFVINVEAQGPAPAPTSSAPSLVSSLFGLLFLAIAAIFA |
| *Glycine max* | GmENODL42 | Glyma16g06241 | VII | MAEQHVVGGSQGWDESTDFNSWVSGRTFMVGDQRVFKYSSLHSVVELGSESEYKNCNIGNAVNSMSSGNDVCMKVKITTVSSSGTSSPSSPSLSSAASASASSSSLVFIAALLIASMLN |
| *Glycine max* | GmENODL43 | Glyma16g34140 | II | MEKLRPAWAVKAIIVIVFTSILFRCVCGENHTVGGASGWDLGSNIQAWSSTTTFNVGDDLVFSYTAAHDVMEVNQLDYDTCKIANALATYDNGETVIHLSDAKTRYFVCGRMGHCQQGLKLQVQILAQSNNGTSNDQNQSPGGSPPSTSPPPPPPPPPPQDDEQPPADEPVPCDCSRAEERHGVVPLITLVIVLAFARTHFFIAFPHLRFHNIR |
| *Glycine max* | GmENODL44 | Glyma17g08110 | VII | MLHPIVKWLSFTTCIPYLLIVNWKSQNPLLYCFALHISLTLLSHIQLINMASCFPNASPFLVMLAMCLLISTSEAEKYVVGGSEKSWKFPLSKPDSLSHWANSHRFKIGDTLIFKYEKRTESVHEVNETDYEGCNTVGKYHIVFNGGNTKVMLTKPGFRHFISGNQSHCQMGLKLAVLVISSNKTKKNLLSPSPSPSPPPSSLLSPSPSPLPNNQGVTSSSGAGFIGVMMWLMLLL |
| *Glycine max* | unknown | Glyma17g12150 | VII | MAFNVTSSYTIRRTRRKNQQKRSSPRIQQGRPHLQEHIAMSRNLLLVLFAVATFLHGSEAQAPAPKATSPISPPRSTPAPGPSSGSVTYTVGETAGWIVPGNASFYPAWASAKNFKVGDILVFNYPSNAHNVEEVTKANYDSCSSASPIATFTTPPARVPLSKSGEHYYICGIPGHCLGGQKLSINVTGGSTATAPTTPSSPSPSGAVSPPPQNSAAASLGLVGVSATLLSVAAAFFYYSIY |
| *Glycine max* | unknown | Glyma17g12160 | VII | MHFQSFSLLIHIGAQKEPSHGPPDTQKQQHMMAQLRNVAILVVVVVAAVLLQSTEAKDYEVGGATGWTSFPPGGASFYSKWAANFTFKLNDSLVFNFESGSHSVVELTKANYENCEVDNNIKAFNRGPARVTLNRTGEFYFSCTFSGHCSSGQKLSIKVTDSSSPAPQKAPAEGPSASAPPPQNAPAEGPNSASPPASGSGSNEGAPSSQTEPAPIAPPPHGSATLLASTFSLFLITIAINFLSHF |
| *Glycine max* | GmSC10 | Glyma17g12170 | II | MANVEILVLLVAAALLMPLRHTTEAAEHVVGGSAGWIIPSQGDTSLYTSFAANNTFRLNDILVFNFATGFHNVVTLSKKHYDSCNVSEVMQSFDTAPARIILNRTGEFYFACAFSSHCSLGQKLSIHVTAGSSSLAPLSPPPPASRAAMPLVAVTIPVLFIAVATNVLF |
| *Glycine max* | GmENODL45 | Glyma17g34040 | II | MAGYSKASALWILFLLFGFSVAKEILVGGKTDAWKVSASESDSLNQWAEKSRFQVGDYLVWKYDGGKDSVLQVSKENYVNCSISNPIKEYNDDTTKVQLEHPGPFYFISGAKGHCEKGQKLVVVVLTPRRGTRFTGFAAPSPAPSASAEFEGPAVAPTSTANALNGGILMVAMGMIAMWVFFM |
| *Glycine max* | GmSC11 | Glyma18g03850 | II | MALSPLSMLVVIATIFLPSVAVAKEFVVGDGHGWTIGFDYAAWAADKTFQVGDVLVFNYAVGEHNVFKVNGTAFQSCTIPPASEALSTGNDRIVLAIPGRKWYICGVEDHCSAGQKLVITVQPQTLPPTLPPSPTPAPSPRQHRPHFGRWVPNIFH |
| *Glycine max* | GmENODL46 | Glyma18g45710 | I | MAPPIAAYITAFSLLITVVSAAETGYHNHTVGGAAGWSFNSTTNTTATNYSSWASTQTFDLGDYLIFNTNSNQTVVQTYNKTTYLNCTAYDSDNGTFVYNGGSRGFGEALTVAVPLTIVGPNYFFSDAGDGVQCQHGLAFEIAVLRGLGLPPSLNQPPPPPYQEPPGPDAAQSPPITVAQSPSGGAFATRADVRVVVYGFVTALVLQFQ |
| *Glycine max* | GmENODL47 | Glyma19g03260 | IX | MENWRGARMMVVASAVAMGWLSVVAMGSPVLHKVGGSKGWINQDVNYTEWSAQEHIYVGDWLIFKFDKRYFNVLEVNKTSYENCIDRDFIKNITRGGRDVVQMTEARTYYYLSGGGYCFHGMRVAVQVQQEDQDPALAMAAPPAPSPVAYGSSSPLPSVCTCIWIIIVANVVVFVN |
| *Glycine max* | GmENODL48 | Glyma19g07631 | II | MASSCSGSLLVLPFVISSTLLCFSVASNEFEVGGSKGWIVPPANDTNFFNDWASQNRFQAGDTIRFKYKKDSVMEVGEGDYTHCNATHPTLFSNNGNTVFKLNHSGTFYFISGASGHCEKGQKMIVRVMADESLSQHAKSSGHHVPVSPIGVSQMLYLQFVLTCVASYVV |
| *Glycine max* | GmENODL49 | Glyma19g18774 | I | MGPKNTIFLALVVTLIAKESFAAQHVVGGSQGWDQSTDFKSWTSGQTSKVGDKLVFKYSSLHSVVELGSESASPPGTRYFTCGTLGHCSQGMKVKITIGRLGNAASPASSSRSLSSPSLSSSTSSSSSPTTTTHASSASQCFTSFMFIIALSVTIMISLF |
| *Glycine max* | GmUC13 | Glyma19g25570 | I | MGRKNTIFLALVVTLITKETMAEQHVVGGSQGWDESTDFNSWVSGQTFKVGDQLVFKYSSLHSVVELGSESEYKNCDLGNAVNSMSSGNDVVKLNKPGTRYFACGTMGHCDQGMKVKITTVSGSETSSPASSSSSSSNSSSASQCLVYLVLIVGLLIASLLN |
| *Glycine max* | GmSC12 | Glyma19g29160 | I | MASAARLAFFAVSMVLLSSVAMATDFTVGDGTGWTVDFNYTAWAEGKVFRVGDTLWFNYENTKHNVVKVNGTQFQECSFTSNNEVLSSGKDSITLKAEGKKWYVCGVANHCAARQMKLVINVETAAPAPAPTSSAHSLLSSVFGVLIVAIAPIFA |
| *Glycine max* | GmENODL50 | Glyma20g01290 | IX | MQTTTTTTKSYFTLCTLIPLFCLPTFVRLYYIGAYIMGLKFTHGVVILIVISASMFSVSMANKDWSFGFNYTDWWSRFGNHPQNKTQQQPRQILVGGSEHWHYGFNYTDWAFKTAPFYLNDTLVFKYDAPNATSFPHSVYMFKSFGSFLKCDIKKAKMLANPTQGSGEGFKFVLKKWQPHYFACGERNGFHCNNGTMKFAVMPMFRPFWKWP |
| *Glycine max* | GmENODL51 | Glyma20g11970 | II | MEVKRKIILCLLIAITMGCYRIEGRDPTLHRVGGGRYTWSPKVNFTKWASHEHFYKGDWLYFGFDKRIYNVLEVNKTNYENCIDTGFIENITRGGRDVFQLLEARHYYFICGRGFCSQGMKLLIDVKEPTTTLPPPILPNKALLNRLSNTLMLVVTILAWIFI |
| *Glycine max* | GmENODL52 | Glyma20g16490 | I | MTNLRNPRFNLFLVSLLVTLVQIQTKVRCYQYKVGDLDSWGIPISPSSQLYDKWSKYHYLSIGDSLLFLYPPSQDSVIQVTEESYKSCNLKDPILYMNNGNSLLNITSEGDFYFTSGEAGHCQKNQKLHITVGVGGNTNALAPTSLPLNAPSYPTVFGNIPMAPSTSSSPHLTSKFSLIIIGFFLCYVHCSLA |
| *Glycine max* | unknown | Glyma20g19611 | VII | MARNVGLNMIGYSTIAMVFIIGVTKATEYMVGDNFGWNVPSYESFYMDWARTKRFHVGDKLIFSWSGEHSLGIRKDDIYYENCSTSLEGGLTYIFNGATINYTIVSTPGPVYFICTVDDHCERGQKFRINVSAAPALSFGILSAFLSSLAVYFCFTVTS |
| *Glycine max* | GmPLC3 | Glyma20g24161 | IX | MPFQLSSQTNYASWAERYNFSSGDVLVFKYVKGQHNVYEVTEDTFRSCDASSGVLAKFETGEDQVALSEVKRYWFICNVAGHCLGGMRFGIEVKDGNNNVTNSTDVAFNPPIEPTPSDNSCTSYYVSERWGVIKNLTPLGLLLFNYYF |
| *Glycine max* | unknown | Glyma20g28210 | VII | MTLVERVVVLFIVMTIVKVSYAAVYKVGDSAGWTTLGTIDYRKWAATKNFQIGDTIIFEYNAKFHNVMRVTHAMYKTCNASSPIATFTTGKDSINITNHGHHFFFCGVPGHCQAGQKVDINVLKVSAEAPTPSGSALASPTVQASTVPAPSPSNATPLISLNGSFGLVGLAMGILSLAFSSYV |
| *Glycine max* | GmENODL53 | Glyma20g33670 | I | MLQHQNPFVLSFSAFFLAFLCHCSATTFTVGDSAGWIIPPYPTYYNNWSHSHFIRVGDSVEFKFDDKFYNLIQVSQKEYQHCTSLEPLRIFNSSPVILPLRERGVLFFICNIPNYCCLGQKIVISVHKDSLEKTPSPSPSQVPITISPHPSSPNASAPQPHGSSSGMSSPPSPTTNTSGGNGGNSPGPSSTQEGKSNAVALVGGRSFTVSLGQLLSMLGAFFGLWVM |
| *Glycine max* | GmENODL54 | Glyma20g33870 | I | MEKVLLVYSLLFSFAIMTCSATTYTVGDSSGWDISTNLDTWIADKNFKVGDALVFQYSSSQSVEEVTKENFDTCNTTNVLATYGSGNTTVPLTRAGGRYYVSGNKLYCLGGMKLHVHVEGDDKSLAPTIAPKAVAGTDQNTSTLPQSPSSKKNTHFSAGAANCARDAIQLVYIALVAAVYGMMKI |
| *Glycine max* | GmENODL55 | Glyma20g35960 | II | MAIFSSHHRMLVSLLLTLVQIQAKVFCYQYKVGDLDAWGIPTSANPQVYTKWSKYHNLTIGDSLLFLYPPSQDSVIQVTEESYKRCNIKDPILYMNNGNSLFNITSKGQFFFTSGEPGHCQKNQKLHISVGEGIIETMDTAPGPSSSLPASAPSYPTVFGNIPVAPSTSTSPQLTSTFQLLIIGFMICAHFASLM |
| *Zea mays* | ZmUC1 | AC203159.3_FGT010 | I | MASANSKQVLLLLAAVACLASLASATQWTVGDVGGWRAKFNETGWTDGKTFTVGDTLLFVYPKENHTVVKVGKDAFAACDLSANLQLGNWTSGSDVVPLDQPGMVWFICNKPNHCLNGMNLAINVVDAATPGAPMAPMTPGAPMAPMTPAPSPAASSPPSAAVRCPVGGAVAAAAAVVVAAALAF |
| *Zea mays* | ZmENODL1 | AC209987.4_FGT010 | I | MAARPVALALPLLALVCALAASADATQFRVGGQSGWSVPGAGSEPYNTWAGRLRFQIGDQLLFVYPKETDSVLLVDAAAYNACNTSSYVSRFDDGSTVFTFDRSGAFFFVSGNEASCRANEKLIVVVLADRSGSRTPTPSSAPLPSPPSPPAAAPAPATSPSSSPPSSAAPAATPTSPTSPALAPAPAPTTTPSSPPAPVPTTTPSSPPAPTPTTPAGGGSPPPTASASAPAAEGGSSTTPPQPSTAAPVVAGFVGSLGAFIGFAMLAA |
| *Zea mays* | ZmSC1 | AC210013.4_FGT002 | II | MAAMGMKKGVLMLALGLAMAVTSSAVVYKVGDASGWTILGNVNYTDWTSKQNFRVGDTIEFTYPPGIHNVLEVNKADYHSCTNSTPIATHTSGDDKIVIKSPGHRFFICGVPGHCAAGQKLSIRVLKTRSSDAPSPAPAARSASAASPSPSGSTSTDPSGAGASASPPASSTDSTPDATTTTAPAPDANGAGVSAGHRAVVAAMAL |
| *Zea mays* | ZmUC2 | AC212835.3_FGT002 | I | MAGTTPRRLLLVIVPLVAVVPASAKDYMVGDSSGWKSGVDYAAWAKGKPFAIGDTLSFQYSSAHSVLEVSEADHGACSASNPLRSHQGQSTTIPLTKAGTRYFICGAPGHCASGMKVAITVSGGGGGGSSSADNTATPSGPSVRATNTKPASGGATTTDESDSSAAGSGARLAMGLLFGAAGLAAIMG |
| *Zea mays* | ZmUC3 | GRMZM2G004012 | II | MAEGRGSARAIGAMAFAVACCYCCVAIADAATTYYVGDSNGWSFSSPSWPNGKHFRAGDTLVFRYIPWIHNVVAVDEDGYNGCTTPPGSRTYTSGADSVTLARGDNFFICTRFGHCNLGMKLVVYAA |
| *Zea mays* | ZmUC4 | GRMZM2G004106 | II | MAAQARMGGARGSSGAVVLALVLLCVLLHGEFAESAVYTVGDRGGWSFNTANWPKGKRFRAGDVLVFKYDPKAHNVVPVSAAGYSSCSAPRGVRALTTGNDRVTLKRGVNYFICSFPGHCQAGMKVAVTAA |
| *Zea mays* | ZmUC5 | GRMZM2G004160 | II | MAAQGSGSARGSGAAAALALVLLCVLLHGELAESAVYTVGDRGGWSFNTANWPKGKRFRAGDVLAFRYNAKAHNVVPVSAAGYKSCSAPKGVRALTTGNDRVTLKRGANYFICSFPGHCQAGMKIAVTAA |
| *Zea mays* | ZmENODL2 | GRMZM2G008106 | VII | MMHAAACAAYIQNPGSASVPFQQQHSRARAHQHDTAKLHSTDTLRSSPPARSMARLLPTAAAAAIVAAVLVAGLPGARAASASPAADDAPPYRNHTVGGGDGWFFDANTNASSGNYSDWAAGETFYLGDYLIFKTNDNSSVVQTTNATTYGLCDASDDLAETTSIYGGGGGGGGGLEQNSTIAVALTAEGANYFFSEADGGAQCQQGMRFEIKVEHGRGLPPSLLHQPPAPKGRVLAPPPAGTAFSGTGGVEPGDGAGDNGGAGTSGACRAASGRFLGVVAAAAAALAVLVL |
| *Zea mays* | ZmUC6 | GRMZM2G008819 | I | MASSWGSRLPLAVVLLLVACFSSTAAATSYTVGDGSGWTTGVDYTSWAASKNFKVGDNLVFNYAKGLHTVVEVSAAEYMACTAANPLGSDSSGATTVALRTPGTHYFVCSITGHCGAGMKLAVTVGGSNSPAATPRTSSPTPTTPYTTPATTTPYTTPTCSGGGGTTATPGMTPFTSYPSAAGLGSAALAGFSLVWCVGVRLALL |
| *Zea mays* | ZmENODL3 | GRMZM2G010762 | I | MGASEVWSPVPAWLAIAVGLAAVVSSSEAHVFYAGGRDGWVLDPTESYNHWAGRSRFQVNDTIVFTHEEGVDSVLLVTEQDFDTCNTRNPVRRLQAVGSSGSSERSVFKLDRSGPFFFISSDEERCQKGQKLYIIVMAVRRSTTPAAPAPDAAFPPAASPVPDSAFPPAPSPVWASAPDNAHAPPPTAGASRLDDGAIIGSVLGVIGALVLCTVY |
| *Zea mays* | ZmENODL4 | GRMZM2G011951 | I | MPMAVILHFAVFAVSCALLVASVLSLPPAVFKVGDERGWTVPANGTETYNHWAKRNRFQVGDVLNFKYANDDSVLLVAHDDYKQCGTAIPLSRFTGGDTKFTLDRYGPLYFVSGVAGHCEAGQRMIVRVRAPSALYGAPAMAPAPAMPPAVGGSGSGAPSPRPASSPAAPSIGGSGSASASAAPSPSPLPQASGGASRRVLGVVSSVAVGLVVLVASAITLSVLVV |
| *Zea mays* | ZmSC2 | GRMZM2G013206 | I | MAAIMGVKKGLLVLALGLAMAATSSAVIYKVGDTSGWTILGNVNYTDWTSKKNFRVGDTIEFTYPPGIHNVLEVKKADYDSCTNSTPIATHSSGDDKIVIKSPGHRFFICGVPGHCAAGQKLNVRVLKTRSSDAPSPAPSARSAAGSAAATPSPTTETTGASSASGGSSTPDATTAPAPNANGAGVGAAGHQNVVVVVVAMAVAALASMTMLH |
| *Zea mays* | ZmUC7 | GRMZM2G023847 | I | MAASRSALITLLVLVSSVAAASATTFTVGDSSGWSRSVNYDNWASGKTFTDGDQLVFNFATGNHDVVEVDKSGYDGCSTTNAANTIQNGPATVNLTSGTHYYICGISGHCSGGMKLAVTVGSGSGSGSGSPTPGGGSPSTPTPTTPSAPAPDTPAGASVRLTAGPALAVAATVLVALF |
| *Zea mays* | ZmUC8 | GRMZM2G027198 | I | MATSRLALVSVFAAAVLLGMASAATYNVGEPAGSWDLRTNYGTWVSSKRFHPGDQTVFKYSPQAHDVVEVNKADYDSCSIASPVATHNSGNDAIALASPGTRYFICGFPGHCDAGMKIQINVVPSANSLGPASAPAANSPVSPPPMSAPSSAATKATGFGALAAVMIAAGLMAY |
| *Zea mays* | ZmPLC1 | GRMZM2G039381 | IX | MPTSRESAVFTVGDRGGWSFSTSTWTNGKRFKAGDVLVFKYDSTAHNVVAVNAAGYKGCSAPRSAKVYTSGNDRVTLARGTNYFICSIPGHCQSGMKIAVPRATYIYLSTA |
| *Zea mays* | ZmUC9 | GRMZM2G043300 | II | MAQRQGRGSGAVALAAAAAVLLCVLLHAHVAESAVFTVGDRGGWSFSTGTWTNGKRFKAGDVLVFKYDSTAHNVVVVNAAGYKGCSAPRGAKVYTSGNDRVTLARGTNYFICSIPGHCQSGMKIAVTAA |
| *Zea mays* | ZmUC10 | GRMZM2G045927 | I | MASSLVSRLPVAVLLLLVACSSTAAATSYTVGDGSGWTSGVDYTSWAASKDFKVGDNLVFNYAKGLHTVVEVSAGEYMACTAANPLGSESDSSGATTVALKAPGTHYYVCSIAGHCGAGMKLAVAVTVGGSNSNSPGATPDDPTTPRTSPTPTAPYTTPTTTTPYTTTPTTTPPYTTPTTTPTTTTPYTTPTCSGGGGATATPGMTPFMSFPSAAGLGSAALAGFGLVWCVIVQLALL |
| *Zea mays* | ZmENODL5 | GRMZM2G047208 | I | MAAAAAGSSRLLSALALAGVVSFLLVADPGVVAAEAAAAPPGLEFRVGGPRGWRVPDANTSYGWWAMNNRFHVGDRLYFKYANDDSVLVVNRLAFDACNASAPLAAFAGGATEFRLHRPGFFCFISGEPGHCEEGQRLIVRVMVHPAALAAAPAPGPGVPTMTRPGMPSGCSDGASSSAVDAAIAAAAGVAMATLVGMGLMLH |
| *Zea mays* | ZmUC11 | GRMZM2G053779 | I | MANALPLIVALVAAAGCAALASATSYTVGDSQGWTTTGVDYSSWASRNTFVVGDTLVFNYVSKAHTVTEVSKAGYDACSGANALSDDDTGSTTITLQTPGTHYFICNVPGHCASGMKLAVAVSASPSGTAPSAGALQVPAMASVVAAAAGAAIKLALF |
| *Zea mays* | ZmENODL6 | GRMZM2G055434 | I | MARRIAGVAALNLLSVLMAATCAAGRDFYVGGRAGWAPNPAEPFNAWAERNRFQVNDTLVFRYSKDADAVLLVSQGHYDACNAAQPAQRLDGGDSRFVFDHSGPYYFISPDAARCRAGERLVVVVLAVRGDGDGDGTPSSSPPPVATPAPSALPTPPPPPHHVPGKNASSPSQGPAPSPALAPAPAPDANGTSSSPPSPSSAVAAFRGGFLLACLLSVTAGAAIILV |
| *Zea mays* | ZmUC12 | GRMZM2G055549 | I | MASSNSKQALLLAAVACLASLASATQWTVGDEGGWRARLNETAWTDGKTFTVGDTLLFVYPKEKHTVVKVGKNAFVACDLSANLQLGNWTSGSDVVTLDQPGMAWFICNKPTHCLNGMKLAIDVVGGTSGPAPMPFPGVPAVAPSPLVRFPFSDLAGPVPDLAALAPAPAPAASSPPSAAVRCPVGGAVAAAAAVVVAAALAL |
| *Zea mays* | ZmENODL7 | GRMZM2G057352 | I | MAASNSSALLPVLALAGVVFLLVAPAVVAEAAAPQGLEFHVGGPRGWRVPDANTSYGWWTMKNRFRVGDHLYFKYTNDSVLLVDRTAFDACNTTEPLATFDDGGTKFVLDRPGFFCFISGEPGHCEEGQRLIVRVMVQPAIVATPGPASGPATSAQPDHGGGHSSGAATAVAAAAVAAALAVFASLV |
| *Zea mays* | ZmENODL8 | GRMZM2G058622 | I | MNKIASAGALLAVVAVVAALAATTASAKDYTVGGSDRWDTYVDYGKWTAGKTFMVGDTITFEYMPYHNVLEVTAADYASCNAGSPISTHSGGSTAFKLTATGTRYFICGIPRHCLNGTMHVTITTVPYDSATAAASGPAQAPLQSSSSPPAADAYAPGPAAGHKVALGAAGKSPAGAPSSAPRYQQPAAAVAGLAVAALVALVA |
| *Zea mays* | ZmENODL9 | GRMZM2G061084 | II | MAPRTALLIATAAMAVIAAAALLPATASATTYMVGDESGWDVGPDYDAWASGKKFKVGDTLEFLYSEGSHNVVVVDAQSYEACAVPSNAPTLTSGDDSVELGQAGRWLFICGVEGHCDAGMKLAVDVHG |
| *Zea mays* | ZmENODL10 | GRMZM2G065498 | VI | MGTLGAAAMATLLTAVVLLTVHVPLATVATDHVVGGSMWSIPLRDDLYMAWSNNRTFYAGDNLVFRFQIGFYDVVQVSRREYEDCTTDDPYNNFRVPPAVVPLDYKGVRYYVCSVGNYCKLGLKFHVTIQQG |
| *Zea mays* | ZmENODL11 | GRMZM2G077769 | I | MRMRARAASASASAAVVVLLLLLLLVGVCAGAVYKVGDLDAWGVPPPSKPDVYKRWAKSIHFALGDSIWFLYPPSQDSVLQLAPAAFASCDLSRPVARLADGNSLFNLTAPGRAYYASGAPGHCRRGQKLWVDVPLPNGTYLQPSATDLAALAPTPAADPPAGFASAAAAAPQGGNASPAPRAAAAAGSVVALSFALQILLL |
| *Zea mays* | ZmUC13 | GRMZM2G082940 | I | MAAITAGLLVLLAAAVAAPAHATDYTVGDSSGWSSGVDYATWASGKTFAAGDNLVFQYSAMHTVVEVSSADYGACSASNSIQSYSDQNTKIALTAPGTRYFICGTPGHCGNGMKLAVTVAAGTATTTPASSPPAADSPPETATPSGSTPTATTSPSAPTTKPTSSSTGAACGGEARLAMGVLAWAAGLAGLALMG |
| *Zea mays* | ZmENODL12 | GRMZM2G085392 | I | MAHGRMMMGCVFLACLLVAASVPSTASAFVFKAGGTGEWRVPAAAAGSNVSAYNAWAQRNRFRVGDAIAFTYQPGKDSVLLVDERSYDACDASSPTDTFADGSTVFTFNRSGPFYFISGNKGNCDRGEKLVVVVMAERAAIGTGGTGLAPSPSSSSSPNGPFSAFSPPPPPFGIDISPSPTAAYPPPSAAPPKMAGLAGAAAFAIIGALFYALV |
| *Zea mays* | ZmENODL13 | GRMZM2G085504 | I | MAASNSSALLPVLALAGVVFFLVAPAVVAEATAPQGLEFHVGGPRGWRVPDANTSYGWWAMNNRFRVGDHLYFKYANDSVLLVDRTAFDACNTTEPLATFADGATKFVLDRPGFFCFISGKPGHCEEGQRLIVRVMVQPAIVATPGPASAPATSAPQPGHGGGHSSGAATAVAAAAGVAAVAALAVFVSLV |
| *Zea mays* | ZmENODL14 | GRMZM2G092338 | I | MANNLLRPLALLLAVLGCALCPRADATAFEVGGDDGWVVPPASDGGRYNQWASKNRFLVGDVVHFKYSEDSVLVVTEADYDSCRASHPVFFSNNGDTEVTLDRPGPVYFISGETGHCERGQRMVVRVAGQGAPPPAPPSPPAPTGGSAAPGTTSGAIAALAMALLAIAIGV |
| *Zea mays* | ZmUC14 | GRMZM2G097851 | I | MAAIMAAGLVVLALLAAVAAPAHATDYVVGDSAGWASGVDYATWASGKTFAAGDNLVFQYSAMHTVAEVSSADYSACSASNSIQSYSDQNTKVALTAPGTRYFICGAPGHCGNGMKLAVTVAAAAATTTPETSTSTPAATTTPTTTNADTPNADTPPETSTSTPAATTPPTTTKSTSTSSTSSTGAACGGEARLGMGILAAAAGLAGLALMG |
| *Zea mays* | ZmENODL15 | GRMZM2G101872 | I | MASSSCALLGLACFVLLAAAAGATQYKVGGDNGWAVPDATAESFNTWAEKTSFQIGDSLLFVYPKDKDSVLLVEPADYNACNTSSYDKQFDDGSTSVALDRAGAFFFISGVEANCRANEKLIVMVAGAGAPAPAPDRQGSGSSSSPSTSAPVPSKGGGAPVPPSSPKAPAAKNSTAKGAPPAAGTDRNGAGAGLAVAGLVASVAGCVAHAMLAL |
| *Zea mays* | ZmENODL16 | GRMZM2G101916 | I | MAKPSSYGLALLACAAVAAAVAGGTQFTVGGANGWSVPAAGAEPFNAWAERTRFQIGDALVFVYPKDQDAVLLVEPAGYNACNTSSYVRKFDDGDTVVALDSAGPLFFISGVEANCRADEKLIVMVLAARSNGTGAPAPSTAPPPPAPASSAPPTPASPAPKGAPPTTAASPPPPSASAPATTPATPPPAASAAPPAPRASSTPPPPSAPAGAPPAPAASSPAPSAHGATDNSTGTPSSPPAGSSKDKNGAAFTVATGLASSFGTCILGYAMLAL |
| *Zea mays* | ZmENODL17 | GRMZM2G102693 | IX | MAMAVALHLGPASAEYYLVGDSAGWTLNYTIGWPENKTFKVDDFLVFRYPRGEYTVTEVDSQTFRECYRQGNAVHEWTSGNDTVRLDSPGRRWFFSSLDDHCDMGLKLFVDVVGSAPPAQHPLPPAQAPAPVVINNETWV |
| *Zea mays* | ZmUC15 | GRMZM2G105682 | I | MAMAKASILATFAAVAALAAVQLAAAADHPVGGDGSWDASGTTGYDAWSAKQTFKQGDTLSFKFAPSHDVTEVSKAGYDACSGSNPVKSYTGGSASVKLSAPGKRYFICSVPGHCAAGMKLEVTVAAAAVTAAPAPAKTTKPRHQRSVSPTPAPAAAPAAPSTDEMPTVSSPTAAPAPKSSDASTLGAKAVVGLAVATMLTFLAM |
| *Zea mays* | ZmSC3 | GRMZM2G107562 | I | MGGGGAALLLLVMWAGVASAAVYEVGGTIGWTVMGNPDYAAWASSKQIVIGDTVVFTYNKQFHNVLAVSKADYKNCIATKPTATWSTGNDSVVLNTTGHHYFLCGYPGHCAAGQKVDIRVASSAAPSAAPSPTPSGSKPSGGATAAPSPHPNAAPKALSASSVAAAVATSLLSLAAAVLA |
| *Zea mays* | ZmUC16 | GRMZM2G116632 | I | MGKMKAVACVACWAALSLINVVMAVDYVVGNPAGGWDGRTDYQSWAAAETFAPGDTLTFKYNSYHSVMEVTKSAFEACTTTDPILYDNSGSTTVALTMPGTRYFICGAPGHCLGGMKMQVQVADRPAPTTPSSPPPPPAHAKQKRHATAPSPTPMPWAPAPTPWSPAPAPAAAPPRRAGHKKKHKKRYCPPETVHAPARAPTPTVQAVEADFPFAAFAPMTAPPPPPPTSGGLAWRRATCGEATAALAALVWFMLL |
| *Zea mays* | ZmSC4 | GRMZM2G121236 | I | MERRRSRHALLLLSAVMASLVAGSTAGIYHIVGAGKGWRMPPNRTYYEDWAHTRQISIGDKLMFLYRSGVHNIVEVPTRELFDACSMRNITSRYQCGPTIIELTDPGERFYFCGVGEHCEAGQKLAINVLLVPPPPPDTDDDSSGAARLLGRAGAGLAAACLCLVSALLTMAV |
| *Zea mays* | ZmUC17 | GRMZM2G122302 | I | MASGGASSLALAALLLVSCASAAVATKYTVGDASGWTTTGDYATWASGKKFKVGDSLEFKYAGGAHTVDEVSAADYAACSSSNALSTDSAGATTVTLKTAGKHYFICGVAGHCSSGMKLVVDVAKAVAAPAPAPAAAAAPAPAADTAPDAPDTTPSTPTSPSSSGGRTPKSPDTTVLSPPDKKSTSSGATGLSAAAWAGLGLAGLVAVHLGAF |
| *Zea mays* | ZmENODL18 | GRMZM2G123407 | I | MVLSGGLVVPWLAAVAALAITAAPLGADAYKNYTVGDDKGWYDGLSLPGVDYQAWADGKNFSLGDFLIFNTDKNHSVVQTRNVTLYESCDYNDSGPDDTVEWAAAPPEFSKDAVTVAVPLLDEGRAYFFSGNYDGEQCESGQRFAVDVAHGQGLPPDLRPPVADAPAPSSAGPADGAAALDFSHPKNVTTPSATDDDDEQLGGGSASSSLRSALPVTATLLITLLFAFAV |
| *Zea mays* | ZmENODL19 | GRMZM2G124667 | I | MAMSYTTLHYFAVSCALLVASVSSLPPPAVFQVGDERGWTVPANGTETYNHWAKRNRFQVGDVLDFKYGANDSVLLVAHDDYKQCSTETPLGRFTGGDTKFALDRYGPVYFVSGVAGHCEAGQRMIVRVIRPGASAPRGAPVAAPATPPTASGSGRSGAPSPATSPAGSGSGSSSTSPSPSPSPSPLPQASGASRRVLVSSVAVGLVLLVACAITLSSVVA |
| *Zea mays* | ZmENODL20 | GRMZM2G128531 | II | MATAARLLLVVVLLSAAIPIPSFATSFVVGDKRHRWAPNVNYTDWADRHQFHVGDWLEFRYERDRFDVVQVNETAYAACDASSPILSYSRGHNFVFRLNHTGRFYFICSRGYCWSGMKVSVLVQPPPPPPSLPPASHSHASSASARVRPAAGLWCAALSTLLGWAVLTPPLSFRVWSSGRPGAHTSLNSKAVC |
| *Zea mays* | ZmUC18 | GRMZM2G136879 | I | MAMQQLLAFASLLLLWPARRASAAEYVVGDVGYGWDSGSGVNYAAWARAHAFAVGDVLVFQYVSTQHNVYEVTEEVYRSCDTAGGDGDGDGVRAKYTSGYDRVVLAEARGYWFICDVPGHCQGGMRVAVNVSAGAAGGGGGSGGPPMTVNPPPPDGSAASTTTAKGRLGWAWAAACLALGVLALMNTSY |
| *Zea mays* | ZmENODL21 | GRMZM2G139193 | I | MAQGRMSCVFLACLVAVASVSASTASAFVFKAGGTGEWRVPAAAGSGNGSSYNAWAQRNRFRVGDAIAFTYQPGNDSVLLVDKRSYDACDTGSPTDTFADGSTVFTFTRSGPFYFISGNKDNCDRGEKLIVVVMAERAAVGNATEPGAGLAPSPSGPFSSFSPPPPFGIDISPTAPYPPPNAAAPPKVAAGVAGAATFAIGALFYALV |
| *Zea mays* | ZmSC5 | GRMZM2G145552 | I | MERRRSRHALLLLSAVMASLVTGSTAGIYHIVGAGKGWRMPPNRTYYEDWARTRQISIGDKLMFLYRSGVHNIVEVPTRELFDACSMRNITSRYQSGPTIIELTDPGERFYFCGVGEHCEAGQKLAINVLLVPPPPPDTDDDCSSAARLPGHAGAGLAAACMCLVSALLTMAV |
| *Zea mays* | ZmENODL22 | GRMZM2G146015 | I | MMGRTSVAAAAAALLVLAACAALPARTAANKISINWKPNVNYTDWLKQHSPFYKDDWLVFYYTAGQADVVQVDEVGYNKCDSTNAIYNYSKGRSFAFQLNETKTYYFICSYGYCFGGMRLAIKAEKLPPPSPPPSASDRSAAAIAAFARSHAPVIYAAVAVLAALLRMV |
| *Zea mays* | ZmENODL23 | GRMZM2G148624 | I | MAGAAFRAVVFSLLGAATFFVGSSSASWHAQVFVVGGEPRGWRKPTAPNEESYNHWAARNRFHVGDFLHFKYEKNDSVLVVTRGDYQLCAADKPTLRFEGGDTRFHLNHSGYCYFISGAPGHCDAGQRMTLRAMVPQQQDGGNNPAAPARAPAAMSPGGEDDEGGTFEPPGARSSTPGSDAGSRPPPHVAAGADGNKTSAAGSMHDASSPPSLRGHRVLGIALAALLMFLPA |
| *Zea mays* | ZmUC19 | GRMZM2G148884 | I | MVMAAAAAVLLALAAVATEVAAAGTTYTVGAPDGLWDMQTDYAQWVKSKTFRPGDTITFTYSPELHDVVEVTRAGYDACSSANNISAFRTGNDAVPLTAVGTRYFLCGLTGHCGNGMKIRVDVVAAAASPSAPGPAAAAAAPPTTSSPCIVGVVAASLLLLLLHAVVSY |
| *Zea mays* | ZmENODL24 | GRMZM2G167578 | I | MANSSNYGLGLACFFAIAAAVAGGTQFMVGGANGWSVPTAGAEPFNTWAERTRFQIGDSLVFVYPKDQDSVLLVEPADYNACDTSSYVRKFDDGDTVVTLDRSGPLFFISGVEANCRANEKLIVMVLAARSNGNGTGGGAQAPSTAPQPASPAASPPPASSTPPPPSSPAPKGASPPPASAPTTTPGTPPPPAPSASSPAPASSTPPPPSAPQAPPPPPASSASSPAPALTTPPPPSATANAPQAPPPPSASSPSPSAHGATASSTGTPSSPPAGAEVKNGAALTVATGLASSFGACILGYAMLAL |
| *Zea mays* | ZmENODL25 | GRMZM2G173103 | I | MLDMGIPTTVMFRVVVIAVFTLSTAAAAGNATASPSSVTNSTASSTKNTTAPLLPPFGTNHAVGDGAGWIFDWKANASAANYSAWAANRTFFLGDYLSFRTDTSNTVVHTTNATAYRFCSAGVAARGGGGGWKPEEAFLVVMLTAEGTNYFFSDALDGEHCRKGMRFQVGVAHGRGLPSVPPSYYEPLSGAPAGTRARHDGAVAVWGVAMVAAFAALAFS |
| *Zea mays* | ZmENODL26 | GRMZM2G174048 | II | MEARRSSAALVSVAAAAAAVALLLVLAAEPSRAERFVVGDAARWTWGYNYTDWVIRKGPFFQNDTLVFMYDPPNATVHAHSVYLMRTAADYQSCNLKAAKLVASVTQGAGSGFEFVLRKRKQHYFVCGERGGIHCTMGQMKFVVKPKSSACRDD |
| *Zea mays* | ZmUC20 | GRMZM2G177934 | II | MAQVRGSARGTGSGSSAAALALVLLCALLRGEFAESAVYTVGDRGGWSFNTASWPNGKRFRAGDVLVFRYDARAHNVVPVSAAGYSSCSAPEGARALATGNDRVTLRRGANYFICSFPGHCQAGMKVAVTAA |
| *Zea mays* | ZmUC21 | GRMZM2G322279 | I | MAYRQVLLLAVAVAVAVACLAPLASAKQWVVGDEGGWRAKFNETGWANGKTFLVGDSLLFVYPKESHTVVKVGKDAFAACDLGANLQLGNWTGGNDVVQLDKPGKAWFICNKPNHCLNGMKLAVDVAVAVAPAPPPSAAPLTGYTVGGAVVAVACAVFAAALAL |
| *Zea mays* | ZmUC22 | GRMZM2G339943 | II | MMMARGRGGAIGGGGVIALVLLVGLFVATSAPPVAEAAASYMVGDYGGWKFNVDRWAKGRTFRAGDVLVFSYNRAVHDVAVVNAAAYRSCAVPNKGARVLRSGRDKVRLGRGTHYFACTVRGHCQAGMKLAVRAV |
| *Zea mays* | ZmUC23 | GRMZM2G352678 | II | MAQGRGSATRGLALGSLLAAAFLLLLGVADAATHRVDWSFNADSWSKGKSFRAGDVLEFNYDPSVHNVVAVDAGGYNGCRPSGTSYGSGSDRITLGPGTSYFICSLNGHCGMGMKMVVNAS |
| *Zea mays* | ZmUC24 | GRMZM2G414317 | II | MAKNSYVVYVLVLLVAGYTAALASATTTTFIVGDDQGWMTGVDYVAWVKGKTFAIGDKLVFNYPSEEHTVTEVSRTDYFACAGGNALSNDRSGSTNITLTGPGTRYFLCNIPGHCTIGMRLAVTVAGGGSPPGATPAGGAAGATARPAMGSFIVKTTAWLAVIKLTLS |
| *Zea mays* | ZmUC25 | GRMZM2G463441 | I | MASKQVLLLLAAVACLASLASATPWTVGDAGGWRAKFNETGWANGKTFVVGDTLLFVYPKENHTVVKVGKDAFAACDLSANLQLGNWTSGSDVVTLDKPGKVWFICNKPNHCLNGMKLAIDVAESAAALAPAPASSSAPVSYTVGAAAAAAVVAAAAVLAF |
| *Zea mays* | ZmUC26 | GRMZM2G485559 | I | MSWLLVAAVLAGFALGPSAGTDHIVGANHGWNPNINYSLWSGNQTFYVGDLISFRYQKGTHNVFEVNETGYDNCTMAGVAGNWTSGKDFIPLPAARRYYFVCGNGFCLQGMKVAITVHPLPHNATSKGSSSHADTGAQEEAAAAALGTRSAAWLATLAVAAAAVAAFC |
| *Zea mays* | ZmPLC2 | GRMZM5G866053 | IX | MAARGRGSAGSSSRAPSVAGTALLCVAVAVLLLAATPAAVAGTTYLVGDAAGWTLKVDYGRWVAGKTFHAGDILVFKYNTTWHDVAWVSKGGYRNCIVSPKGRAPVYHTGYDAVTLPRGTHYFICAMPGHCSAGMKLAVTVY |
| *Zea mays* | ZmPLC3 | GRMZM5G881965 | IX | MPWLPAQCRTESKHSMAMAQARVVALALCALLLVSGVARRAEAVSYNVGNSAGWDLSADLPSWADGKTFNVGDVLVFQYSSYHTLDEVDQAGFNNCSAANALLSRSDGNTTVPLTAPGDRYFICGSQLHCLGGMKLHVLVSQPAGGAPAKATPQSTPQTGSGAALGPSTDDAGLAGIPWLVLGGSHRATVGSMLLTWLFVATALLLV |

^a^: “XXX”: signal peptide; “XXX”: PCLD, plastocyanin-like domain; “X”: N-glycosylation site; “XXX”: putative AG site; “XXX”: Putative arabinosylation site; “XXX”: GPI-anchor signal.

Note: The N-glycosylation sites were predicted only in the protein containing N-secretion signals.
